# Unveiling the correlation between high-entropy alloy element systems and electrocatalytic activity

**DOI:** 10.1093/nsr/nwag161

**Published:** 2026-03-14

**Authors:** Xiangyi Shan, Furong Cai, Yuanhua Tu, Di Zhang, Yiyang Pan, Lunbo Chen, Jinhui Liang, Han Gao, Jianan Xu, Hao Li, Jing Li, Erkang Wang, Min Zhou

**Affiliations:** State Key Laboratory of Electroanalytical Chemistry, Changchun Institute of Applied Chemistry, Chinese Academy of Sciences, Changchun 130022, China; School of Applied Chemistry and Engineering, University of Science and Technology of China, Hefei 230026, China; State Key Laboratory of Electroanalytical Chemistry, Changchun Institute of Applied Chemistry, Chinese Academy of Sciences, Changchun 130022, China; Guangdong Provincial Key Laboratory of Fuel Cell Technology, School of Chemistry and Chemical Engineering, South China University of Technology, Guangzhou 510641, China; Advanced Institute for Materials Research (WPI-AIMR), Tohoku University, Sendai 980-8577, Japan; State Key Laboratory of Electroanalytical Chemistry, Changchun Institute of Applied Chemistry, Chinese Academy of Sciences, Changchun 130022, China; School of Applied Chemistry and Engineering, University of Science and Technology of China, Hefei 230026, China; State Key Laboratory of Electroanalytical Chemistry, Changchun Institute of Applied Chemistry, Chinese Academy of Sciences, Changchun 130022, China; School of Applied Chemistry and Engineering, University of Science and Technology of China, Hefei 230026, China; Guangdong Provincial Key Laboratory of Fuel Cell Technology, School of Chemistry and Chemical Engineering, South China University of Technology, Guangzhou 510641, China; State Key Laboratory of Electroanalytical Chemistry, Changchun Institute of Applied Chemistry, Chinese Academy of Sciences, Changchun 130022, China; State Key Laboratory of Electroanalytical Chemistry, Changchun Institute of Applied Chemistry, Chinese Academy of Sciences, Changchun 130022, China; Advanced Institute for Materials Research (WPI-AIMR), Tohoku University, Sendai 980-8577, Japan; State Key Laboratory of Electroanalytical Chemistry, Changchun Institute of Applied Chemistry, Chinese Academy of Sciences, Changchun 130022, China; School of Applied Chemistry and Engineering, University of Science and Technology of China, Hefei 230026, China; State Key Laboratory of Electroanalytical Chemistry, Changchun Institute of Applied Chemistry, Chinese Academy of Sciences, Changchun 130022, China; School of Applied Chemistry and Engineering, University of Science and Technology of China, Hefei 230026, China; State Key Laboratory of Electroanalytical Chemistry, Changchun Institute of Applied Chemistry, Chinese Academy of Sciences, Changchun 130022, China; School of Applied Chemistry and Engineering, University of Science and Technology of China, Hefei 230026, China

**Keywords:** high-entropy alloys, oxygen reduction reaction, large language models, high-throughput experimentation, elemental synergistic effects

## Abstract

The complex element combinations and synergistic effects of high-entropy alloys (HEAs) present significant challenges for efficient exploration and optimal design. Here, we propose a collaborative framework that integrates large language models (LLMs) with a high-throughput platform, aiming to reveal the correlation between HEA element systems and oxygen reduction reaction (ORR) activity. By domain-specific fine-tuning of the LLM, we developed ChatHEA as an assistant for the enumeration of HEA combinations, which enabled rapid synthesis via a high-throughput platform and facilitated the construction of a standardized ORR activity dataset through batch performance evaluation. Subsequently, ChatHEA performs multidimensional analysis and pattern recognition on the dataset to uncover the intrinsic relationships between HEA element systems and ORR activity, followed by detailed validation of the superior combinations. Density functional theory (DFT) and advanced pH-dependent microkinetic modeling further elucidate and confirm the facilitating effect of elemental synergism on reaction activity. This collaborative framework combining LLMs, high-throughput experimentation, and advanced modeling offers a new pathway for the efficient development of catalytic materials and mechanistic understanding.

## Teaser

By integrating large language models with a high-throughput experimental platform, an efficient collaborative framework is developed to systematically uncover correlations between high-entropy alloy systems and oxygen reduction reaction activity.

## INTRODUCTION

High-entropy alloys (HEAs), as an emerging class of multi-element alloy systems [1,2], have attracted extensive attention in the field of energy catalysis due to their rich element combinations, exceptional structural stability, and outstanding catalytic performance [3–6]. The diversity of element combinations not only endows HEAs with tunable electrochemical properties but also optimizes their microstructures and electronic features, thereby enhancing catalytic reaction efficiency [7,8]. Consequently, the rapid screening of high-performance HEA combinations and compositions using high-throughput experimentation [5,9,10] and theoretical calculations [11,12] has become a central focus of current research. However, due to the complex synergistic interactions and non-linear characteristics among multi-elements in HEA systems, systematically unveiling the intrinsic correlation between element systems and catalytic activity remains a significant challenge [2,7,13].

In recent years, machine learning (ML) has emerged as an important tool for accelerating the discovery and optimization of HEA electrocatalysts [13,14]. Different categories of algorithms exhibit distinct advantages at their respective levels: graph neural networks (GNNs) and their derivatives can learn structure–property mappings directly from atomic configurations, enabling the prediction of adsorption energies and catalytic activities [11,15,16]; Bayesian optimization combined with Gaussian processes allows efficient exploration of high-dimensional compositional spaces with minimal experimental cost to identify optimal catalysts [17–19]; meanwhile, methods such as random forest, gradient boosting and support vector regression have been widely employed to establish quantitative structure–activity relationships from high-throughput experimentation or theoretical datasets [10,20,21]. However, these approaches generally rely on structured input formats, and their predictive capabilities are largely confined to the ‘known data-performance prediction’ model [22]. In the context of HEA research, where data scarcity and knowledge fragmentation remain prominent challenges, conventional ML algorithms can efficiently optimize compositions within specific datasets but struggle to extract and integrate unstructured prior knowledge embedded in the literature, nor can they transfer knowledge across stages or assist in experimental pathway planning [23].

In contrast, large language models (LLMs), endowed with powerful natural language understanding and generation capabilities [24,25], offer a new pathway to overcome the aforementioned limitations. Unlike most ML models that rely on structured data and serve single-purpose functions, LLMs can automatically extract and integrate domain knowledge from massive volumes of unstructured literature [26], thereby providing strong support for the construction of prior knowledge bases that can guide experimental design [23,27–29]. Moreover, LLMs possess intrinsic advantages in cross-task coordination and decision-making assistance, empowering the entire research workflow—from materials design and experimental planning to data analysis and mechanistic interpretation [30–32]. Therefore, in addressing the challenges of complex elemental compositions and fragmented knowledge systems in HEA electrocatalysis research, leveraging LLMs to establish a domain knowledge-enhanced collaborative framework holds great promise for breaking down data silos and task fragmentation, while providing intelligent support for deciphering the intricate features of multi-element material systems.

Here, we propose a collaborative framework integrating LLMs with a high-throughput platform to reveal the correlation between HEA element systems and oxygen reduction reaction (ORR) activity. By fine-tuning the LLM with a large-scale literature corpus, we developed an artificial intelligence (AI)-based assistant named ChatHEA tailored for HEA electrocatalysis research, which is capable of constraint-based enumeration of HEA combinations, experimental planning and data analysis. Leveraging the high-throughput platform and rotating ring-disk electrode (RRDE), we systematically synthesized and evaluated a series of HEA catalysts organized under ChatHEA-assisted enumeration, thereby constructing a standardized ORR activity dataset. Subsequently, ChatHEA performed automated data processing and feature analysis, successfully revealing the correlation between HEA element systems and ORR activity, and identifying several promising HEA combinations, which were further validated in detail. In addition, density functional theory (DFT) calculations with advanced pH-dependent microkinetic modeling were employed to confirm the synergistic effects of specific element systems in promoting ORR activity. This collaborative framework effectively reduces the research barriers associated with multi-element complex material systems, offering a novel approach for the systematic exploration and mechanistic understanding of HEA electrocatalysts.

## RESULTS AND DISCUSSION

### LLM-driven collaborative framework

**Figure 1.**
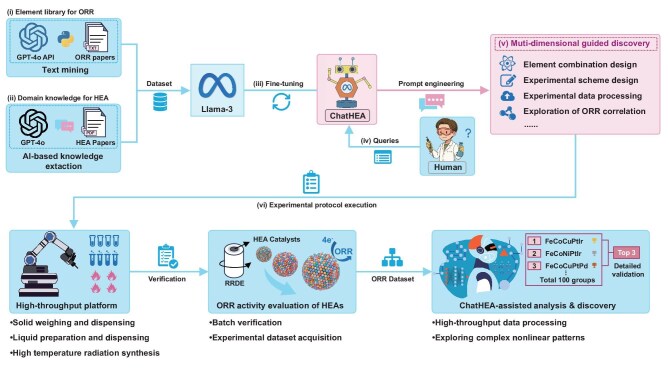
Collaborative framework of LLMs and the high-throughput experimental platform. The embedded GPT and Llama logos indicate that the process is driven by the corresponding models, owned by OpenAI and Meta, respectively. The double-snake Python icon denotes the use of Python and is owned by the Python Software Foundation.

This study developed a collaborative framework integrating LLMs with a high-throughput platform (Fig. 1), comprising six key steps. First, customized Python scripts called the GPT-4o application programming interface (API) were used, with prompt-engineering strategies to mine 17 000 ORR-related abstracts [5,10,26] (Figs S1 and S2) and extract an element library for ORR research (Fig. S3). Building on this, GPT-4o was further employed to conduct AI-based domain knowledge extraction on 200 items of carefully selected HEA literature, and the extracted content was subsequently reviewed, filtered and structured by domain experts to construct a multidimensional knowledge dataset covering the HEA electrocatalysis research field. Based on this large-scale dataset, the Llama-3-8B model was fine-tuned using low-rank adaptation (LoRA), leading to ChatHEA (Fig. S4), an AI assistant tailored for HEA electrocatalysis. Unlike ML algorithms that primarily aim to learn composition–performance mappings from structured data [33,34], the core capability of ChatHEA lies in its ability to comprehend and utilize domain knowledge for reasoning and generation. In this study, ChatHEA does not function as a substitutive ‘predictive black box,’ but rather serves as an intelligent central hub that drives the entire workflow of combinatorial enumeration, experimental scheme recommendation, and data-assisted analysis.

Through targeted fine-tuning, the model exhibited significantly enhanced task comprehension and instruction–response stability in HEA-ORR research scenarios compared to the pre-tuned version (Figs S5–S7), with outputs more closely aligned with specific experimental requirements. It should be noted that such directed optimization aims to improve domain-specific performance rather than expand the model’s general capabilities. In this study, ChatHEA was positioned as a research assistant operating under explicit scientific objectives and expert-defined constraints—its core function is not to replace human decision-making but to systematically enhance research planning and execution efficiency and consistency by integrating large-scale prior knowledge, high-throughput platform parameters, and data analysis workflows. Based on the practical requirements proposed by the experimental team (Fig. S8), and under the constraints of literature-derived prior knowledge and chemical intuition, ChatHEA assisted in enumerating 100 quinary HEA combinations (Table S1) designed for correlation discovery and characterized by systematic combinatorial commonality, enabling a comprehensive exploration of the relationships between elemental systems and ORR activity. At this stage, ChatHEA demonstrated distinct advantages. Unlike approaches that rely on rule-based scripts or trial and error guided by expert intuition, it efficiently integrated literature priors, physicochemical properties of precursors, and multiple chemical constraints, achieving systematic and reproducible enumeration through natural language interaction tailored to a clear objective—constructing a highly comparable sample library to uncover correlation. This process provided HEA combination schemes that are both chemically rational and comparatively valuable, serving as robust starting points for subsequent high-throughput experimental investigations. Furthermore, ChatHEA generated task lists based on prompt-engineering strategies and, leveraging the functional parameters of the high-throughput platform, performed task-level optimization across synthesis, activity evaluation, and data analysis workflows (Fig. S9), thereby reducing the researchers’ operational workload. Collectively, these outputs highlight ChatHEA’s role as the intelligent core of the collaborative framework—translating fine-tuned domain knowledge into executable research blueprints, and providing a structured foundation for high-throughput experimentation and data analytics. Entering the experimental phase, the high-throughput platform rapidly synthesized the 100 HEA combinations and assessed their ORR activities via RRDE measurements, thereby establishing a standardized activity dataset. Finally, through ChatHEA-assisted data analysis, the intrinsic correlation between HEA element systems and ORR activity was systematically uncovered, leading to the identification of three high-performance HEA combinations characterized by distinct elemental synergistic features.

### High-throughput synthesis and characterization

**Figure 2.**
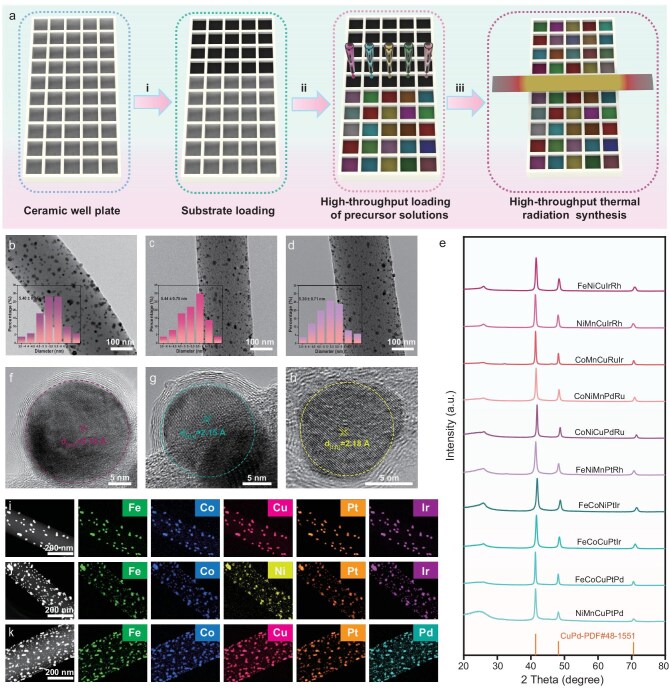
High-throughput synthesis and characterization of HEA catalysts. (a) Schematic workflow for the high-throughput thermal radiation synthesis of HEA catalysts. (b–d) TEM images of FeCoCuPtIr/FeCoNiPtIr/FeCoCuPtPd@CA-CNFs (insets: size distribution histograms of HEA nanoparticles). (e) XRD patterns of HEAs@CA-CNFs. (f–h) HRTEM images of FeCoCuPtIr/FeCoNiPtIr/FeCoCuPtPd@CA-CNFs and (i–k) corresponding HAADF-STEM images and EDS mappings.

Figure 2a illustrates the high-throughput synthesis workflow for 100 HEA catalysts. Based on the experimental scheme generated by ChatHEA, the synthesis process was carried out on a high-throughput platform using an arrayed ceramic well plate loaded with a CO_2_-activated carbon nanofiber (CA-CNF) substrate. A high-throughput solid–liquid dispensing workstation was employed to precisely formulate and uniformly load HEA precursor solutions (Figs S10–S12). Subsequently, a high-temperature heat source was scanned line by line across the sample surface and applied in repeated pulse-mode radiation heating (Fig. S13), enabling contactless high-throughput synthesis of HEA catalysts. Due to the difficulty of real-time temperature monitoring in this non-contact process, a COMSOL model [35] was developed to simulate the transient surface temperature distribution during heating (Figs S14 and S15). Simulation results indicated that the method satisfies the thermal conditions required for HEA formation (Table S2), and no significant thermal interference was observed between adjacent samples during sequential heating (Fig. S16).

Multiple physical characterizations confirmed the successful synthesis of HEAs@CA-CNFs. Scanning electron microscopy (SEM) images revealed that HEA nanoparticles with different element combinations exhibited comparable loading densities and similar particle size distributions (Fig. S17), which was further corroborated by transmission electron microscopy (TEM) images (Fig. 2b–d), ensuring the reliability and consistency of the ORR activity dataset [36]. X-ray diffraction (XRD) patterns indicate that the HEAs with various element systems exhibit face-centered cubic (FCC) structures (Fig. 2e, Fig. S18), and X-ray photoelectron spectroscopy (XPS) analysis further confirms the formation of single-phase alloys (Fig. S19). High-resolution TEM (HRTEM) images (Fig. 2f–h) showed characteristic lattice spacings of 2.16/2.15/2.18 Å, corresponding to the (111) planes identified in the XRD patterns. High-angle annular dark-field scanning transmission electron microscopy (HAADF-STEM) combined with energy-dispersive X-ray spectroscopy (EDS) mappings demonstrated the uniform distribution of elements within the nanoparticles (Fig. 2i–k). Inductively coupled plasma optical emission spectrometry (ICP-OES) confirmed that the element compositions of the HEAs@CA-CNFs were consistent with the intended precursor feeding ratios (Fig. S20). Additionally, to validate the scalability of this approach, a similar sample was successfully synthesized on a carbon fiber paper (CFP) substrate (Fig. S21, HEAs@CFP).

### ORR activity assessment and correlation analysis

**Figure 3.**
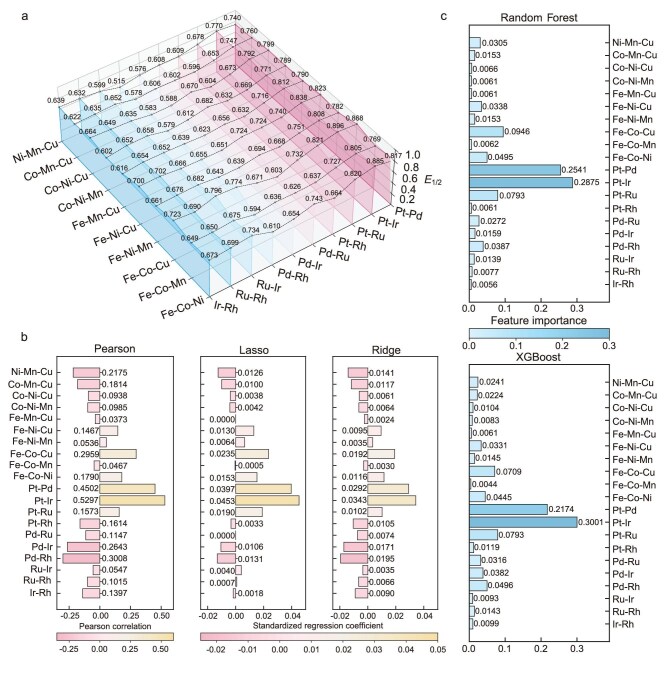
ORR activity dataset and correlation/feature importance analysis of 100 HEA combinations. (a) Dataset of *E*_½_ for 100 HEA combinations. (b) Correlation analysis between NNM/NM element systems and *E*_½_ based on Pearson correlation, Lasso and Ridge regression models. (c) Feature importance analysis between NNM/NM element systems and *E*_½_ based on Random Forest and XGBoost models.

To construct a high-quality ORR activity dataset, RRDE was employed to evaluate the ORR performance of 100 HEA catalysts, following high-throughput synthesis that significantly improved sample preparation efficiency. The entire evaluation process was completed within 5 working days. Multiple sets of parallel samples exhibited highly consistent results (Fig. S22), further validating the reliability of the adopted high-throughput synthesis and assessment methodology. Subsequently, all experimental data were automatically preprocessed and structurally integrated by ChatHEA (Figs S23 and S24), forming a standardized ORR activity dataset that provided a solid foundation for subsequent correlation analysis. Figure 3a presents the distribution of half-wave potentials (*E*_½_) corresponding to the 100 HEA combinations. Further analysis based on the generation–collection mode of RRDE confirmed that all catalysts followed a 4e^−^ transfer process (Fig. S25), thereby offering a robust basis for comparative activity evaluation and pattern discovery across different HEA element systems.

After constructing a standardized ORR activity dataset, we employed the ChatHEA assistant to perform a systematic modeling analysis of the correlation between HEA element systems and *E*_½_. ChatHEA first recommended using the Pearson correlation coefficient as a preliminary evaluation metric, supplemented by Lasso and Ridge regression models as linear reference algorithms (Figs S26–S29). The Pearson correlation coefficient offers strong interpretability, while Lasso and Ridge models introduce regularization terms to enhance model stability and enable feature selection. As shown in Fig. 3b, three non-noble metal (NNM) systems—Fe–Co–Cu, Fe–Co–Ni and Fe–Ni–Cu—along with two noble metal (NM) systems—Pt–Ir and Pt–Pd—exhibited a pronounced positive correlation with *E*_½_, maintaining high contribution weights across multiple linear algorithms. Notably, when the analysis dimension was reduced to single-element encoding (Fig. S30), only Pt and Fe displayed strong positive correlation, while Co, Ni and Cu showed near-zero correlation. This contrast indicates that the catalytic activity of HEAs is not dominated by the linear contribution of individual elements, but rather arises from complex multi-elemental synergistic coupling—where the isolated contributions of single elements are weakened but become significantly amplified through combinatorial interactions within the alloy system. To verify the robustness of the linear-model conclusions, and further examine non-linear relationships among multidimensional features, we introduced two non-linear models—Random Forest and XGBoost—for supplementary analysis (Fig. 3c). Unlike linear regression, these models assess the relative contribution of each element system to variations in *E*_½_ through feature importance, without distinguishing between positive and negative directions. Despite their different modeling logic, the results of both non-linear models were highly consistent with the linear models in the ranking of major element systems, jointly identifying Fe–Co–Cu, Fe–Co–Ni, Fe–Ni–Cu, Pt–Ir and Pt–Pd as key contributing systems. This strong consistency across algorithmic frameworks demonstrates the robustness and universality of the conclusions, providing a reliable foundation and directional guidance for the subsequent selection and experimental validation of high-activity HEA combinations.

### ORR performance validation

**Figure 4.**
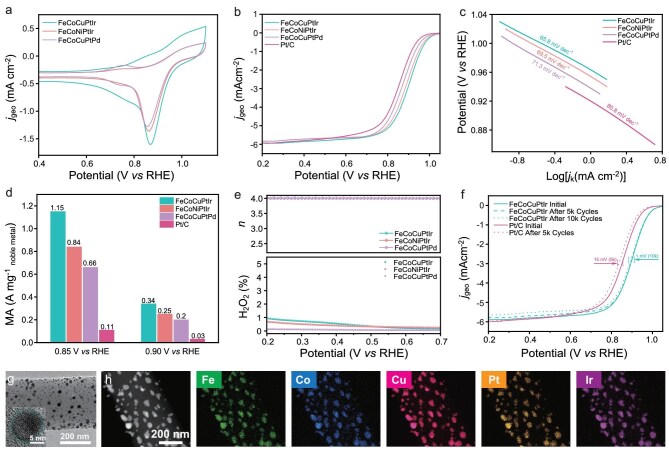
ORR evaluation of high-performance HEA combinations. (a) CV curves of three HEA catalysts. (b) LSV curves and (c) Tafel plots of three HEA catalysts and Pt/C at 1600 r/min. (d) MA comparison of three HEA catalysts and Pt/C. (e) Electron transfer numbers and H_2_O_2_ yields of three HEA catalysts. (f) Comparison of ADT for FeCoCuPtIr and Pt/C. (g) TEM image of FeCoCuPtIr@CA-CNFs after ADT (inset: HRTEM image of a single particle), and (h) HAADF-STEM image with corresponding EDS mappings.

To further validate the three high-performance HEA combinations identified by ChatHEA, their ORR performance was systematically evaluated. The cyclic voltammetry (CV) curves in Fig. 4a show that FeCoCuPtIr exhibits the highest peak potential and peak intensity, reflecting its superior ORR activity. Comparative analysis revealed that all three HEA catalysts outperformed commercial Pt/C (0.852 V, 80.8 mV dec^−1^) in both *E*_½_ and Tafel slope (Fig. 4b and c). Notably, FeCoCuPtIr achieved an *E*_½_ of 0.894 V and a Tafel slope of 65.8 mV dec^−1^, which are comparable to those of advanced NM-based alloy catalysts reported in the literature (Table S3). In addition, the performance of Pt/C in this study was consistent with literature benchmarks (Table S4), confirming the reliability of the testing system. At 0.85 V vs. reversible hydrogen electrode (RHE), the mass activities (MAs) of three HEA catalysts were 1.15, 0.84 and 0.66 A mg^−1^ (Fig. 4d), corresponding to 10.5, 7.6 and 6.0 times that of Pt/C (0.11 A mg^−1^), respectively, showing a clear and consistent activity gradient. Analysis based on the RRDE generation–collection mode and Koutecký–Levich fitting (Fig. 4e, Fig. S31) confirmed that all three catalysts follow a 4e^−^ transfer process. After 5000 cycles of accelerated degradation testing (ADT), the linear sweep voltammetry (LSV) curves of FeCoCuPtIr showed negligible change, whereas Pt/C exhibited a 15 mV drop in *E*_½_, highlighting the excellent stability of the HEA catalyst (Fig. 4f). Post-ADT morphology and XRD analysis revealed that FeCoCuPtIr retained its particle structure without evident aggregation (Fig. 4g) or phase separation (Fig. 4h, Fig. S32), which is attributed to the structural stability conferred by the high-entropy effect. Overall, FeCoCuPtIr exhibits clear advantages in both ORR activity and stability, effectively validating the feasibility and practical value of the LLMs and high-throughput experimental collaborative framework proposed in this study for the efficient discovery of HEA catalysts.

Given the outstanding ORR performance of FeCoCuPtIr in the RRDE system, we further conducted device-level validation in a proton exchange membrane fuel cell (PEMFC) to evaluate its performance under realistic operating conditions (Fig. S33a). The PEMFC testing allows comprehensive assessment of catalyst behavior under multi-coupled conditions, including gas diffusion, electrolyte transport and electrode interfacial stability [37]. Following the U.S. Department of Energy (DOE) fuel cell testing protocol, H_2_–air fuel cells were assembled using FeCoCuPtIr or commercial 20 wt% Pt/C as the cathode and 40 wt% Pt/C as the anode. Under operation at 80°C and a 150 kPa_abs_ backpressure, the FeCoCuPtIr-based fuel cell achieved a peak power density of 0.789 W cm^−2^, surpassing that of commercial Pt/C (0.724 W cm^−2^), and exhibited a consistently higher current density across the entire polarization curve (Fig. S33b), highlighting its superior high-loading operational capability. Furthermore, based on the H_2_–air polarization curve, FeCoCuPtIr achieved an MA of 0.806 A mg_noble metal_^−1^ at 0.9 V*_iR_*_-free_, which is 1.83 times higher than the DOE 2025 activity target (0.44 A mg_noble metal_^−1^) [38]. These results clearly demonstrate the remarkable kinetic advantage and practical application potential of the FeCoCuPtIr system under high-potential operating conditions.

### Mechanistic origin of enhanced ORR activity

**Figure 5.**
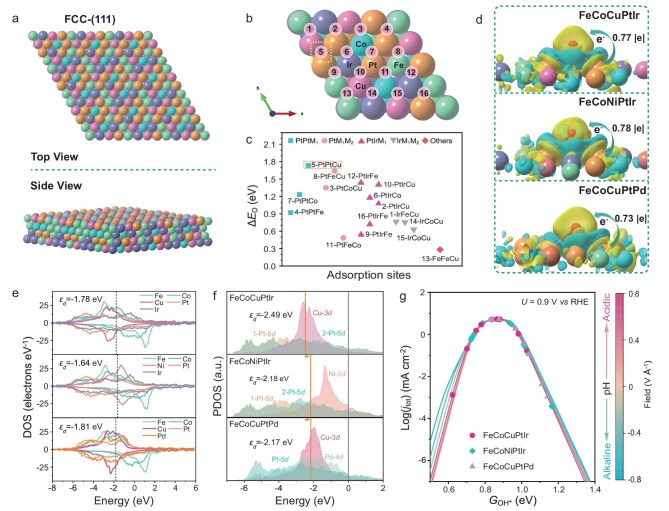
Mechanistic analysis of ORR on high-performance HEA combinations. (a) Top and side views of the FeCoCuPtIr–(111) surface model. (b) Hollow-FCC adsorption sites on the FeCoCuPtIr–(111) surface and (c) the corresponding Δ*E*_O_ values. (d) Differential charge density distribution of O adsorption on the three HEA surfaces. (e) DOS plots of three HEA combinations. (f) PDOS at representative active sites on three HEA surfaces. (g) pH-dependent microkinetic volcano model for the overall ORR at 0.9 V vs. RHE at varying pH and fields.

The three high-activity HEA combinations identified by the ChatHEA assistant—FeCoCuPtIr, FeCoNiPtIr and FeCoCuPtPd—demonstrate a consistent preference toward Fe–Co–Cu and Fe–Co–Ni NNM systems, as well as Pt–Ir and Pt–Pd NM systems. Based on their compositions and phase structures, and following the computational schemes generated by ChatHEA (Fig. S34), three corresponding models were constructed (Fig. 5a, Fig. S35), followed by subsequent computational analyses [39,40]. It is important to emphasize that the adsorption sites on HEA surfaces are extremely diverse, making exhaustive site screening practically infeasible [12,41]. Therefore, inspired by the work of Nørskov *et al*. [42,43], we evaluated the oxygen adsorption energy (Δ*E*_O_) across multiple representative sites based on the ORR volcano relationship (Fig. 5b and c, Fig. S36) and selected active sites with moderate binding strength (∼1.8 eV, near the volcano peak) on the HEA-(111) surfaces [6]. Differential charge density analysis incorporating quantitative charge-transfer values (Δ*q*) (Fig. 5d, Fig. S37) revealed a moderate redistribution of interfacial electrons during O* adsorption. The appropriate Δ*q* indicates that electron transfer is neither excessively strong (which would over-stabilize O*) nor too weak (which would hinder activation), a trend consistent with the calculated Δ*E*_O_ results. The density of states (DOS) analysis (Fig. 5e) further showed that the incorporation of Fe, Co, Ni and Cu optimizes the electronic structure of the HEA surface by inducing an overall downward shift of the *d*-band center (*ε_d_*), thereby weakening the O*–surface interaction and reducing the adsorption strength. Specifically, the electronic states of Fe, Co and Cu tend to shift away from the Fermi level (*E*_F_), resulting in a negative shift of *ε_d_* in neighboring Pt atoms. This effect further attenuates O* adsorption, leading to moderate stabilization of intermediates and facilitating desorption, which collectively lower the reaction barrier and enhance ORR kinetics. Moreover, projected DOS (PDOS) analysis (Fig. 5f) confirmed that the *ε_d_* values of different active sites progressively shift away from *E*_F_, consistent with the overall DOS results and in full agreement with the *ε_d_* theory in predicting O* adsorption strength.

To comprehensively capture the ensemble-effect contributions of HEA surface active sites, a multi-local coordination environment sampling and statistical evaluation framework was adopted to analyze the adsorption energy distributions of the key adsorption descriptor (*G*_OH*_) across multiple local configurations of the three HEA systems (Fig. S38). Taking the optimal apex (∼0.85 eV) of the pH-dependent ORR microkinetic volcano model [44–46] (Fig. 5g) as a reference, the distribution centers of *G*_OH*_ for FeCoCuPtIr, FeCoNiPtIr and FeCoCuPtPd are 0.81, 0.74 and 0.96 eV, respectively. Among them, FeCoCuPtIr exhibits a distribution center closest to the optimal range, indicating that, in a statistical sense, it possesses a higher proportion of ‘near-optimal’ adsorption sites, thereby demonstrating superior intrinsic ORR activity at the ensemble level. Furthermore, mapping the sampled local-site *G*_OH*_ values onto the microkinetic volcano plot reveals that FeCoCuPtIr shows a denser population of active sites near the volcano apex, corresponding to a stronger theoretical ORR kinetic response. In contrast, the low-activity sample NiMnCuPdRh, modeled using the same computational framework (Fig. S39), exhibits a site distribution that deviates significantly from the optimal region, with most sites located at the volcano periphery (Fig. S40). Overall, the differences in adsorption energy distributions closely mirror the experimentally observed activity trends, providing a statistically grounded mechanistic explanation for the macroscopic performance variations among HEA catalysts and validating the effectiveness of the theoretical screening framework developed in this study.

## CONCLUSION

In summary, taking HEA catalysts as representative examples of complex multi-element systems, this study proposes a collaborative framework integrating LLMs with a high-throughput experimental platform. Under the constraints of literature-derived prior knowledge and chemical intuition, this framework achieved the AI-assisted enumeration, high-throughput synthesis, and ORR activity evaluation of 100 HEA catalysts, thereby constructing an activity–correlation dataset oriented toward the ORR. Through ChatHEA-assisted automated data processing and multidimensional feature analysis, the synergistic enhancement contributions of element systems such as Fe–Co–Cu and Fe–Co–Ni with Pt–Ir and Pt–Pd were systematically revealed, and three high-performance HEA combinations (FeCoCuPtIr, FeCoNiPtIr and FeCoCuPtPd) were successfully identified and experimentally validated. Further DFT calculations and pH-dependent microkinetic modeling elucidated the underlying mechanisms of elemental synergy and explained the electronic structure modulation that promotes ORR kinetics. Importantly, this study not only provides fundamental insights and mechanistic understanding for the optimization and discovery of HEA catalysts but also establishes a generalizable methodological framework that offers new perspectives and technical pathways for the development of AI-driven automated laboratories, providing valuable implications for the deep integration of data-driven science and electrocatalysis research.

It is also important to recognize that, although this study has preliminarily demonstrated the potential of LLMs and high-throughput collaboration in exploring HEA electrocatalysts, the current framework still has room for improvement in both predictive capability and active guidance mechanisms. For instance, the framework has not yet implemented intelligent prioritization of candidate materials or active learning strategies to systematically reduce experimental workload. Moreover, the sample scale remains constrained by multiple factors, including synthesis throughput, characterization efficiency and screening rate [32,47], which inherently limit the in-depth exploration of broader compositional spaces. Looking forward, further progress can be achieved by developing integrated high-throughput synthesis-characterization platforms [28], incorporating multimodal AI models [27], introducing Bayesian optimization and reinforcement learning strategies [30], and establishing cross-scale computational–experimental collaboration mechanisms [13]. In parallel, promoting the standardization and sharing of materials databases [48] will enable AI-guided, predictive and efficient exploration across larger compositional spaces, gradually forming a truly self-driving ‘rational design–experimental validation’ research loop [15,49,50]. Furthermore, extending such intelligent frameworks to device-level performance evaluation and practical application scenarios will help accelerate the translation of HEA catalysts from laboratory research to engineering implementation, fostering a closed-loop, autonomous and systematic paradigm for AI-empowered materials innovation.

## MATERIALS AND METHODS

All chemical reagents were purchased from Shanghai Aladdin Biochemical Technology Co., Ltd. Detailed descriptions of LLM fine-tuning, synthesis methods, material characterization, electrochemical tests, COMSOL simulations and DFT calculation methods can be found in Section S1 of the Supplementary data.

## Supplementary Material

nwag161_Supplemental_File

## Data Availability

The model code and data processing code presented in this paper can be accessed at https://github.com/SuperResolutionElectrochemistry/Correlation_analysis_and_data_processing.

## References

[1] Yao Y, Huang Z, Xie P et al. Carbothermal shock synthesis of high-entropy-alloy nanoparticles. Science 2018; 359: 1489–94. 10.1126/science.aan5412 29599236

[2] Ren J-T, Chen L, Wang H-Y et al. High-entropy alloys in electrocatalysis: from fundamentals to applications. Chem Soc Rev 2023; 52: 8319–73. 10.1039/D3CS00557G 37920962

[3] Xiong H, Dong Y, Hu C et al. Highly efficient and selective light-driven dry reforming of methane by a carbon exchange mechanism. J Am Chem Soc 2024; 146: 9465–75. 10.1021/jacs.4c02427 38507822

[4] Xiong H, Ji X, Mao K et al. Light-driven reverse water gas shift reaction with 1000-h stability on high-entropy alloy catalysts. Adv Mater 2024; 36: 2409689. 10.1002/adma.202409689 39279322

[5] Pan Y, Shan X, Cai F et al. Accelerating the discovery of oxygen reduction electrocatalysts: high-throughput screening of element combinations in Pt-based high-entropy alloys. Angew Chem Int Ed 2024; 63: e202407116. 10.1002/anie.202407116 38934207

[6] Feng G, Ning F, Pan Y et al. Engineering structurally ordered high-entropy intermetallic nanoparticles with high-activity facets for oxygen reduction in practical fuel cells. J Am Chem Soc 2023; 145: 11140–50. 10.1021/jacs.3c00868 37161344

[7] Hsu W-L, Tsai C-W, Yeh A-C et al. Clarifying the four core effects of high-entropy materials. Nat Rev Chem 2024; 8: 471–85. 10.1038/s41570-024-00602-5 38698142

[8] Liang J, Cao G, Zeng M et al. Controllable synthesis of high-entropy alloys. Chem Soc Rev 2024; 53: 6021–41. 10.1039/D4CS00034J 38738520

[9] Yao Y, Huang Z, Li T et al. High-throughput, combinatorial synthesis of multimetallic nanoclusters. Proc Natl Acad Sci USA 2020; 117: 6316–22. 10.1073/pnas.1903721117 32156723 PMC7104385

[10] Shan X, Pan Y, Cai F et al. Accelerating the discovery of efficient high-entropy alloy electrocatalysts: high-throughput experimentation and data-driven strategies. Nano Lett 2024; 24: 11632–40. 10.1021/acs.nanolett.4c03208 39225654

[11] Zhang J, Wang C, Huang S et al. Design high-entropy electrocatalyst via interpretable deep graph attention learning. Joule 2023; 7: 1832–51. 10.1016/j.joule.2023.06.003

[12] Batchelor TAA, Pedersen JK, Winther SH et al. High-entropy alloys as a discovery platform for electrocatalysis. Joule 2019; 3: 834–45. 10.1016/j.joule.2018.12.015

[13] Yao Y, Dong Q, Brozena A et al. High-entropy nanoparticles: synthesis-structure-property relationships and data-driven discovery. Science 2022; 376: eabn3103. 10.1126/science.abn3103 35389801

[14] Zheng X, Wang T, Lu Y et al. Navigating complexity to design nanostructured high-entropy alloy catalysts. Adv Energy Mater 2025; 16: e04545. 10.1002/aenm.202504545

[15] Chang Y, Benlolo I, Bai Y et al. High-entropy alloy electrocatalysts screened using machine learning informed by quantum-inspired similarity analysis. Matter 2024; 7: 4099–113. 10.1016/j.matt.2024.10.001

[16] Zhang J, Su W, Li Y et al. Boosting screening of nonequiatomic high-entropy electrocatalysts by inverse design via active graph learning. ACS Catal 2026; 16: 323–32. 10.1021/acscatal.5c05945

[17] Zhu Q, Zhang F, Huang Y et al. An all-round AI-Chemist with a scientific mind. Natl Sci Rev 2022; 9: nwac190. 10.1093/nsr/nwac190 36415316 PMC9674120

[18] Pedersen JK, Clausen CM, Krysiak OA et al. Bayesian optimization of high-entropy alloy compositions for electrocatalytic oxygen reduction. Angew Chem Int Ed 2021; 60: 24144–52. 10.1002/anie.202108116 PMC8596574 34506069

[19] Xu W, Diesen E, He T et al. Discovering high entropy alloy electrocatalysts in vast composition spaces with multiobjective optimization. J Am Chem Soc 2024; 146: 7698–707. 10.1021/jacs.3c14486 38466356 PMC10958507

[20] Wang Z, Chen X, Lin T et al. Machine learning-guided design of l12-type Pt-based high-entropy intermetallic compound for electrocatalytic hydrogen evolution. Adv Mater 2025; 38: e10424. 10.1002/adma.202510424 41090465

[21] Nam HN, Nandan R, Fu L et al. Modeling-making-modulating high-entropy alloy with activated water-dissociation centers for superior electrocatalysis. J Am Chem Soc 2025; 147: 33545–58. 10.1021/jacs.5c08012 40899870

[22] Deng H, Yang L-M. Energy catalysis through high-entropy materials by experiment, computation, and artificial intelligence. Matter 2026; 9: 102487. 10.1016/j.matt.2025.102487

[23] Gao T, Huang H, Liu Y. Machine learning-driven nanoscale synthesis for electrocatalytic performance: from data-driven methodologies to closed-loop optimization. Adv Mater 2025; e08263. 10.1002/adma.202508263 41294096 PMC13549291

[24] Zheng Z, Rong Z, Rampal N et al. A GPT-4 reticular chemist for guiding MOF discovery. Angew Chem Int Ed 2023; 62: e202311983. 10.1002/anie.202311983 37798813

[25] Zheng Z, Alawadhi AH, Chheda S et al. Shaping the water-harvesting behavior of metal–organic frameworks aided by fine-tuned GPT models. J Am Chem Soc 2023; 145: 28284–95. 10.1021/jacs.3c12086 38090755

[26] Jin Z, Liu K, Pan Z et al. Thermally stabilized hydrogenation dynamics in single-atom alloys enables selective CO_2_ electroreduction. J Am Chem Soc 2026; 148: 622–31. 10.1021/jacs.5c15278 41307500

[27] Song T, Luo M, Zhang X et al. A multiagent-driven robotic AI chemist enabling autonomous chemical research on demand. J Am Chem Soc 2025; 147: 12534–45. 10.1021/jacs.4c17738 40056128

[28] Boiko DA, MacKnight R, Kline B et al. Autonomous chemical research with large language models. Nature 2023; 624: 570–8. 10.1038/s41586-023-06792-0 38123806 PMC10733136

[29] Fu Z, Huang P, Wang X et al. Artificial intelligence-assisted ultrafast high-throughput screening of high-entropy hydrogen evolution reaction catalysts. Adv Energy Mater 2025; 15: 2500744. 10.1002/aenm.202500744

[30] Zhang Z, Ren Z, Hsu C-W et al. A multimodal robotic platform for multi-element electrocatalyst discovery. Nature 2025; 647: 390–6. 10.1038/s41586-025-09640-5 40987343

[31] Szymanski NJ, Rendy B, Fei Y et al. An autonomous laboratory for the accelerated synthesis of novel materials. Nature 2023; 624: 86–91. 10.1038/s41586-023-06734-w 38030721 PMC10700133

[32] Cooper AI, Courtney P, Darvish K et al. Accelerating discovery in natural science laboratories with AI and robotics: perspectives and challenges. Sci Robot 2025; 10: eadv7932. 10.1126/scirobotics.adv7932 40991717

[33] Fan X, Chen L, Huang D et al. From single metals to high-entropy alloys: how machine learning accelerates the development of metal electrocatalysts. Adv Funct Mater 2024; 34: 2401887. 10.1002/adfm.202401887

[34] Hart GLW, Mueller T, Toher C et al. Machine learning for alloys. Nat Rev Mater 2021; 6: 730–55. 10.1038/s41578-021-00340-w

[35] Zheng X, Gao X, Vilá RA et al. Hydrogen-substituted graphdiyne-assisted ultrafast sparking synthesis of metastable nanomaterials. Nat Nanotechnol 2023; 18: 153–9. 10.1038/s41565-022-01272-4 36585516

[36] Nesselberger M, Roefzaad M, Fayçal Hamou R et al. The effect of particle proximity on the oxygen reduction rate of size-selected platinum clusters. Nat Mater 2013; 12: 919–24. 10.1038/nmat3712 23872730

[37] Lazaridis T, Stühmeier BM, Gasteiger HA et al. Capabilities and limitations of rotating disk electrodes versus membrane electrode assemblies in the investigation of electrocatalysts. Nat Catal 2022; 5: 363–73. 10.1038/s41929-022-00776-5

[38] Xiao F, Wang Q, Xu G-L et al. Atomically dispersed Pt and Fe sites and Pt–Fe nanoparticles for durable proton exchange membrane fuel cells. Nat Catal 2022; 5: 503–12. 10.1038/s41929-022-00796-1

[39] Chen T, Zhang X, Wang H et al. Antisite defect unleashes catalytic potential in high-entropy intermetallics for oxygen reduction reaction. Nat Commun 2025; 16: 3308. 10.1038/s41467-025-58679-5 40195379 PMC11977229

[40] Zhao X, Cheng H, Chen X et al. Multiple metal–nitrogen bonds synergistically boosting the activity and durability of high-entropy alloy electrocatalysts. J Am Chem Soc 2024; 146: 3010–22. 10.1021/jacs.3c08177 38278519 PMC10859931

[41] Lu Z, Chen ZW, Singh CV. Neural network-assisted development of high-entropy alloy catalysts: decoupling ligand and coordination effects. Matter 2020; 3: 1318–33. 10.1016/j.matt.2020.07.029

[42] Nørskov JK, Rossmeisl J, Logadottir A et al. Origin of the overpotential for oxygen reduction at a fuel-cell cathode. J Phys Chem B 2004; 108: 17886–92. 10.1021/jp047349j 39682080

[43] Kulkarni A, Siahrostami S, Patel A et al. Understanding catalytic activity trends in the oxygen reduction reaction. Chem Rev 2018; 118: 2302–12. 10.1021/acs.chemrev.7b00488 29405702

[44] Kelly SR, Kirk C, Chan K et al. Electric field effects in oxygen reduction kinetics: rationalizing pH dependence at the Pt(111), Au(111), and Au(100) electrodes. J Phys Chem C 2020; 124: 14581–91. 10.1021/acs.jpcc.0c02127

[45] Zhang D, She F, Chen J et al. Why do weak-binding M–N–C single-atom catalysts possess anomalously high oxygen reduction activity? J Am Chem Soc 2025; 147: 6076–86. 10.1021/jacs.4c16733 39924878 PMC11848820

[46] Ye S, Wang Y, Liu H et al. Decoding pH-dependent electrocatalysis through electric field models and microkinetic volcanoes. J Mater Chem A 2025; 13: 37821–32. 10.1039/D5TA06105A

[47] Liu X, Liang J, Wang Z et al. Building catalyst exploration highways by integrating high-throughput and machine learning technologies. Adv Energy Mater 2026; 16: e05497. 10.1002/aenm.202505497

[48] Gregoire JM, Zhou L, Haber JA. Combinatorial synthesis for AI-driven materials discovery. Nat Synth 2023; 2: 493–504. 10.1038/s44160-023-00251-4

[49] Tabor DP, Roch LM, Saikin SK et al. Accelerating the discovery of materials for clean energy in the era of smart automation. Nat Rev Mater 2018; 3: 5–20. 10.1038/s41578-018-0005-z

[50] Schilling-Wilhelmi M, Ríos-García M, Shabih S et al. From text to insight: large language models for chemical data extraction. Chem Soc Rev 2025; 54: 1125–50. 10.1039/D4CS00913D 39703015

