# Supplementary material for: Unveiling the correlation between high-entropy alloy element systems and electrocatalytic activity
Source: Natl Sci Rev. 2026 Mar 14;13(11):nwag161. doi: 10.1093/nsr/nwag161 (PMC13289745; doi:10.1093/nsr/nwag161)
Supplement: nwag161_Supplemental_File [file nwag161_supplemental_file.pdf]

Supporting Information

Unveiling the Correlation Between High-Entropy Alloy Element Systems and Electrocatalytic Activity

Xiangyi Shan[1,2], Furong Cai[1], Yuanhua Tu[3], Di Zhang[4], Yiyang Pan[1,2], Lunbo Chen[1,2], Jinhui Liang[3], Han Gao[1], Jianan Xu[1], Hao Li[4]*, Jing Li[1,2], Erkang Wang[1,2]* and Min Zhou[1,2]*

[1] State Key Laboratory of Electroanalytical Chemistry, Changchun Institute of Applied Chemistry, Chinese Academy of Sciences, Changchun 130022, China.

[2] School of Applied Chemistry and Engineering, University of Science and Technology of China, Hefei 230026, China.

[3] Guangdong Provincial Key Laboratory of Fuel Cell Technology, School of Chemistry and Chemical Engineering, South China University of Technology, Guangzhou 510641, China.

[4] Advanced Institute for Materials Research (WPI-AIMR), Tohoku University, Sendai, Japan, 980-8577.

*Corresponding authors.
*E-mail address:

Hao Li, li.hao.b8@tohoku.ac.jp

Erkang Wang, ekwang@ciac.ac.cn

Min Zhou, mzhou1982@ciac.ac.cn

Table of Contents

Section S1. Experimental Section S3
S1.1 Chemicals and Materials. S3
S1.2 LLMs and Data Availability S3
S1.2.1 Acquisition of Literature Datasets for ChatHEA Development S4
S1.2.2 Prompt Engineering and Data Extraction Methods. S4
S1.2.3 Extraction of the ORR Element Library via ChatGPT API-Based Text Mining ... S6
S1.2.4 Development of the ChatHEA Assistant Based on LoRA Fine-Tuning S7
S1.2.5 Python Code S8
S1.2.6 Limitations and Future Directions of LLMs in Experimental Planning S8
S1.3 High-Throughput Platform S9
S1.4 High-Throughput Synthesis of HEA Catalysts S9
S1.4.1 Preparation of CA-CNFs S9
S1.4.2 High-Throughput Precursor Solution Preparation and Loading S9
S1.4.3 High-Throughput Thermal Radiation Synthesis of HEAs@CA-CNFs S10
S1.5 COMSOL Simulation S10
S1.6 Physicochemical Characterization S11
S1.7 Electrochemical Evaluation S11
S1.8 PEMFC Testing S12
S1.9 Correlation Analysis S13
S1.10 Computational Methods S14
Section S2. Supplementary Figures and Tables S17
References: S62

Section S1. Experimental Section

S1.1 Chemicals and Materials.

Iron(III) chloride hexahydrate ($FeCl_3·6H_2O$, 99%), cobalt(II) chloride hexahydrate ($CoCl_2·6H_2O$, AR), nickel(II) chloride hexahydrate ($NiCl_2·6H_2O$, AR), copper(II) chloride dihydrate ($CuCl_2·2H_2O$, AR), manganese(II) chloride tetrahydrate ($MnCl_2·4H_2O$, 99%), chloroplatinic acid hexahydrate ($H_2PtCl_6·6H_2O$, AR), palladium(II) chloride ($PdCl_2$, Pd 59-60%), rhodium(III) chloride hydrate ($RhCl_3·xH_2O$, 99.95%), ruthenium(III) chloride hydrate ($RuCl_3·xH_2O$, 99.95%), iridium(IV) chloride hydrate ($IrCl_4·xH_2O$, 99.9%), Nafion 117 solution (~5 wt.%), isopropanol (AR, ≥99.7%), and N,N-dimethylformamide (DMF, AR, 99.5%) were all purchased from Aladdin. Commercial 20 wt.% Pt/C catalyst (HISPEC 3000) was obtained from Johnson Matthey. Perchloric acid ($HClO_4$, 99.999%) was purchased from Sigma-Aldrich. Deionized water (Millipore Milli-Q, 18.25 MΩ) was used for all aqueous solution preparations. All chemicals were used as received without further purification.

S1.2 LLMs and Data Availability

In this study, we employed two representative advanced large language models (LLMs): ChatGPT-4o and Meta-Llama-3-8B. ChatGPT-4o, released and continuously maintained by OpenAI in 2024, is an upgraded version of the GPT-4 series, featuring multimodal processing capabilities and outstanding semantic parsing performance. We utilized customized Python scripts to call the (application programming interface) API version of ChatGPT-4o to perform automated mining of 17,000 oxygen reduction reaction (ORR)-related literature abstracts, thereby efficiently constructing a systematic ORR element library. In addition, for in-depth data mining of 200 high-quality high-entropy alloy (HEA) electrocatalysis papers, we directly employed the ChatGPT-4o backbone model as the core, leveraging its stronger interdisciplinary comprehension and knowledge integration capabilities to establish a multidimensional knowledge dataset covering the HEA research field. In comparison, Meta-Llama-3-8B, one of the third-generation Llama series models released by Meta AI in 2024, contains approximately 8 billion parameters. Although smaller in scale than ChatGPT-4o, its open-source nature and flexibility allowed us to fine-tune the model with the low-rank adaptation (LoRA) method, enabling it to better absorb domain-specific knowledge in HEA electrocatalysis and providing customized advantages in the construction of the ChatHEA assistant. Overall, the ChatGPT-4o API version was mainly responsible for large-scale automated knowledge mining, the ChatGPT-4o backbone model performed deep semantic understanding and knowledge integration, and

Meta-Llama-3-8B enhanced domain adaptability through LoRA fine-tuning. Together, these three components jointly supported the development of the AI research assistant ChatHEA.

Details on the specific task execution with the ChatGPT models and the development workflow of the ChatHEA assistant are provided in the subsequent Supporting Information section. Furthermore, the programs used for LLM-based abstract text mining, the spot-checking results of the ORR element library, and the source codes generated by the ChatHEA assistant have been publicly released in a Github repository, accessible via the following link.

*Code of this project could be access from GitHub:

https://github.com/SuperResolutionElectrochemistry/Correlation_analysis_and_data_processing

S1.2.1 Acquisition of Literature Datasets for ChatHEA Development

To efficiently construct an element library suitable for ORR research, we first batch-exported 17,000 research article abstracts related to electrocatalytic ORR, published between 2015 and May 2024, from the publicly available Clarivate© Web of Science database. This large-scale abstract dataset provided the model with a broad research context and statistical foundation, thereby establishing a solid data source for subsequent element system screening and frequency analysis.

On this basis, we further collected 200 PDF research papers on HEA electrocatalysis, published between 2018 and 2024, spanning 25 different journals and authorized by six publishing groups, including Springer Nature, the American Association for the Advancement of Science (AAAS), Elsevier, the Royal Society of Chemistry (RSC), the American Chemical Society (ACS), and Wiley. This high-quality literature dataset not only contained detailed information on HEA synthesis methods, physical characterization, and electrochemical performance, but also provided crucial support at the deep semantic level for ChatHEA's domain-specific fine-tuning and knowledge extraction.

S1.2.2 Prompt Engineering and Data Extraction Methods.

In constructing the corpus dataset for fine-tuning the Meta-Llama-3-8B model, we designed and implemented a systematic prompt engineering strategy to address the complexity and predominantly unstructured nature of the literature content, thereby ensuring the efficient extraction and standardized organization of key information. This strategy first explicitly defined the extraction targets within the prompts, covering multiple core dimensions such as HEA elemental composition, synthesis methods and conditions,

physical characterization results, electrochemical performance metrics, and theoretical calculation conclusions, while specifying a unified output format to minimize ambiguities and omissions in the model's generated outputs.

Second, domain-specific keywords and constraints were incorporated into the prompts, including commonly used catalytic terminology, standard electrochemical parameters, and synthesis-related terminology, which significantly improved the accuracy and consistency of information extraction. To further mitigate potential "hallucinations" and errors under complex contexts, we introduced multiple verification mechanisms into the prompt design, such as defining candidate answer ranges, adding contextual constraints, and requiring the model to provide explanations for internal consistency.

Building upon this foundation, we developed a structured knowledge extraction workflow designed for dual-layer literature datasets (17,000 abstracts and 200 full-text PDFs), enabling multi-dimensional information integration from shallow statistical features to deep semantic representations. For the abstract-level data, the prompt engineering process followed a three-step pipeline of "element recognition–semantic filtering–standardized conversion." Specifically, all chemical element entities were automatically identified using a named entity recognition module; non-metallic elements and non-catalysis-related descriptions were excluded through a combination of regular expression matching and contextual constraints; and all recognized entities were converted into standardized chemical symbols to construct a normalized element frequency database exported as a fixed-format CSV file. For the full-text-level data, a "dimension-driven + template-constrained" prompt system was designed, corresponding to five core dimensions: elemental composition, synthesis conditions, characterization results, electrochemical performance, and theoretical calculation conclusions. Each template was defined with fixed-format JSON output fields and controlled terminology rules to ensure compatibility and comparability of extracted information across different studies during structured storage and subsequent data analysis. To enhance the reproducibility and reliability of the extraction process, all prompt templates underwent no fewer than three iterative optimization rounds, each validated through few-shot comparative experiments to evaluate the model's accuracy and consistency. The final optimized template system achieved an average extraction accuracy exceeding 95% across different literature types, as verified by expert inspection, thereby ensuring structural consistency across heterogeneous datasets.

In addition, to address semantic ambiguities arising from non-standard expressions in certain papers (e.g., "Fe/Ni-based catalysts" or "Pt–Ru alloying"), a rule-based postprocessing script was implemented to perform symbol normalization, unit conversion,

and terminology unification (for example, standardizing "half-wave potential" and "$E_{1/2}$" as equivalent terms), ensuring consistency and scientific rigor throughout the entire data-processing pipeline.

S1.2.3 Extraction of the ORR Element Library via ChatGPT API-Based Text Mining

For the construction of the ORR element library, we built upon our previous work [1-2] and employed Python scripts to call the GPT-4o API developed by OpenAI, performing research domain classification and metal element extraction from 17,000 literature abstracts. To minimize hallucinations and errors generated by the LLM during processing [3-4], we adopted role-based preset instructions and iterative optimization of prompt engineering to improve the accuracy and stability of text extraction.

To quantitatively evaluate the performance of the LLM in extracting metallic elements, we randomly selected 300 abstracts based on literature identifiers for manual validation. This validation size is considered highly representative in natural language processing tasks [5-6]. To ensure the scientific rigor and independence of the evaluation, the validation was independently carried out by three doctoral researchers with extensive experience in HEA electrocatalyst design and energy catalysis. We employed a strict "document-level + complete extraction" criterion for accuracy assessment, requiring the LLM to correctly identify all metallic elements mentioned in the literature, thereby avoiding overestimation of model performance due to partial extraction. In addition, to avoid confusion across evaluation dimensions, other information such as reaction systems and research domains was excluded from this assessment.

The validation results showed that the overall accuracy of metallic element extraction by the LLM was 97%, with 9 abstracts flagged as "errors," including 7 false positives and 2 false negatives. The false positives mainly arose from misclassification of non-metallic elements in doped catalysts (e.g., N, P, S, Se) as metallic elements. Since only metallic elements were retained in the subsequent frequency statistics, these misclassifications did not affect the final distribution trends. The false negatives were mainly due to the use of non-standardized expressions (e.g., full names, abbreviations, or semantic variants) to describe metallic elements, which prevented the model from recognizing them accurately. Although multiple iterations of prompt optimization were implemented, completely eliminating hallucinations in LLM outputs remains a challenge [7-9]. It is worth noting that given the already high accuracy (97%) and considering that our subsequent analysis focused on "high-frequency elements," these minor errors did not substantively affect the frequency distribution results. Therefore, we conclude that the current strategy achieves a reasonable balance among accuracy, feasibility, and cost control. The constructed ORR

element library thus provides a valuable reference and robust support for subsequent HEA combinatorial constrained enumeration.

S1.2.4 Development of the ChatHEA Assistant Based on LoRA Fine-Tuning

To develop ChatHEA, a research assistant tailored for HEA electrocatalysis, we fine-tuned the Meta-Llama-3-8B-Instruct model using the LoRA method within the Llama-Factory framework. This process aimed to enhance the model's comprehensive understanding and planning capabilities in the HEA electrocatalysis domain. First, a large-scale instruction dataset was constructed, covering over 20,000 rigorously curated samples related to HEA catalysis and ORR research. The dataset encompassed full-chain knowledge including elemental statistical data, experimental methodologies, and high-throughput instrumentation workflows. All data were formatted according to the Llama-3 instruction template and truncated to 4096 tokens to ensure consistent input structure and improve training stability.

In terms of fine-tuning strategy and parameter configuration, we adopted the LoRA-based efficient fine-tuning approach, updating only a small number of low-rank adapter parameters. This allowed effective domain knowledge injection while greatly reducing computational costs. The key LoRA parameters were set as r=8, α=16, and dropout=0.05, striking a balance between representational capacity and parameter efficiency. The base model weights were frozen to avoid catastrophic forgetting. To achieve progressive domain adaptation from broad knowledge fusion to core capability enhancement, a two-stage fine-tuning strategy was implemented.

In Stage 1, the model was trained for 1 epoch on the complete instruction corpus covering various HEA-ORR tasks to establish a comprehensive understanding of domain knowledge structures and reasoning paradigms. The AdamW optimizer was used (β_1=0.9, β_2=0.999, weight decay=0.1) with an initial learning rate of 1e-4. A cosine annealing schedule with 50-step linear warmup was applied to ensure smooth convergence during early training. The per-device batch size was set to 1, and gradient accumulation over 16 steps provided an effectively larger batch size. In addition, bf16 mixed-precision training and gradient checkpointing were enabled to optimize memory utilization and maintain training stability.

In Stage 2, to further improve the model's reliability in core tasks such as HEA combinatorial constrained enumeration, fine-tuning was performed on a more strictly filtered subset of the corpus. The weights from Stage 1 were loaded, and the learning rate was reduced to 8e-6, while other hyperparameters remained unchanged. Training continued for 900 steps. This low-learning-rate fine-adjustment phase aimed to reinforce

adherence to professional design criteria and experimental feasibility while preventing over-specialization that could harm general reasoning ability.

To ensure both training stability and generalization, an independent validation set was employed in both stages to monitor model performance. The optimal model was automatically selected based on validation loss, and early stopping together with gradient clipping were applied to prevent overfitting and numerical divergence. Beyond validation-set evaluation, the fine-tuning performance was also assessed through expert evaluations on real research tasks. Domain experts compared the model's responses before and after fine-tuning across representative tasks such as combinatorial enumeration and experimental planning. The fine-tuned ChatHEA showed substantial improvements in relevance, accuracy, and practicality, demonstrating that LoRA-fine-tuned Llama-3 effectively enhanced both usability and domain expertise within HEA–ORR research scenarios.

S1.2.5 Python Code

The codes used in this study for batch data processing and analysis, statistical algorithms, machine learning (ML) models, and automated plotting were generated by the ChatHEA research assistant through multiple rounds of interactive dialogues based on the specific requirements of the experimental researchers. All codes were subsequently reviewed, tested, and refined by professional programmers. The relevant codes are available in the public repository referenced in Section S1.2 of the Supporting Information.

S1.2.6 Limitations and Future Directions of LLMs in Experimental Planning

It should be noted that the application of LLMs in experimental planning remains in a stage of rapid development and continuous refinement. Since their knowledge primarily derives from training corpora, the recommendations generated by LLMs may be influenced by the timeliness, coverage, and prevailing research trends of the underlying data, which limits their capacity to stimulate highly innovative or disruptive ideas. At the same time, LLMs still differ from human experts in their understanding of complex physicochemical mechanisms, and their outputs involving experimental safety and risk quantification require careful verification based on professional judgment. In addition, efficiently integrating multi-modal experimental data (such as spectroscopic and imaging information) and dynamic resource constraints presents new challenges for current text-based LLMs. Therefore, this study positions LLMs as efficient auxiliary tools, emphasizing their practicality in large-scale experimental planning and knowledge integration, while underscoring the researcher's leading role in critical decision-making. Looking ahead, with the deeper integration of

domain knowledge bases, computational models, and explainability-enhancement techniques, LLMs are expected to demonstrate broader potential in intelligent scientific research assistance, providing stronger support for the exploration and optimization of complex material systems.

S1.3 High-Throughput Platform

In this study, a suite of automated high-throughput workstations (CAS Applied Chemistry Science & Technology Co. Ltd., Changchun, https://www.mintech-instrument.com/) was employed, including a high-throughput solid dispensing workstation (HTP-SD), a high-throughput liquid dispensing workstation (HTP-LD), and a high-throughput advanced materials synthesis workstation (ESHOCK-2024A, Particle Precision Instruments Co. Ltd., Jilin Province, http://www.hightemsyn.com/). Together, these systems constituted the integrated high-throughput platform used throughout this work. Based on the functional capabilities of each workstation, experimental workflows were optimized with the assistance of ChatHEA. Human researcher was only responsible for tasks such as sample transfer and consumable replenishment, with no need for additional complex operations or significant time investment. This arrangement allowed researchers to focus more effectively on core scientific tasks.

S1.4 High-Throughput Synthesis of HEA Catalysts

S1.4.1 Preparation of CA-CNFs

To obtain a high-performance support suitable for loading HEA catalysts, CO_2-activated carbon nanofibers (CA-CNFs) were prepared via carbonization and CO_2 activation of polyacrylonitrile (PAN) fibers. Specifically, PAN was first dissolved in DMF (8 wt.%) and stirred overnight at room temperature to ensure a homogeneous solution. Electrospinning was then conducted under 35% humidity using the following parameters: a spinning voltage of 10.0 kV, a spinning distance of 15 cm, a flow rate of 0.02 mm min^{-1}, and a collector rotation speed of 80 rpm, with continuous operation for over 12 hours. The collected fibers were subsequently stabilized in a muffle furnace at 533 K for 5 hours. Carbonization was performed in an argon atmosphere at 1073 K for 2 hours using a tubular furnace. Finally, to introduce surface defects and increase the loading capacity of HEA nanoparticles, the CNFs were activated in a CO_2 atmosphere at 1023 K for 3 hours, yielding the final CA-CNFs support.

S1.4.2 High-Throughput Precursor Solution Preparation and Loading

To significantly improve experimental throughput, equal masses of CA-CNFs were first placed into ceramic well plates. A high-throughput solid dispensing workstation was used

to automatically weigh the metal precursor salts, while a high-throughput liquid dispensing workstation was employed to prepare and load the precursor solutions. All precursor salts were dissolved in ethanol to a concentration of 0.05 mol L^{-1}. The dispensing program was designed according to the 100 HEA combinations generated by ChatHEA (Table S1). All combinations were prepared and loaded at equimolar atomic ratios, with a fixed metal loading of 10 wt.%.

S1.4.3 High-Throughput Thermal Radiation Synthesis of HEAs@CA-CNFs

High-throughput thermal radiation synthesis of HEAs@CA-CNFs was performed under an argon atmosphere using a high-throughput advanced materials synthesis workstation. The ceramic orifice plate containing metal-loaded CA-CNFs were placed beneath a carbon paper heater, and the power supply was adjusted to maintain a heating temperature of ~2100 K. The workstation was operated in line-by-line heating mode using the following parameters: heating time of 2.0 s, cooling time of 5 s, and five heating cycles. Upon completion of the synthesis program, the resulting catalysts were transferred to an argon-filled glovebox to prevent potential oxidation during storage.

S1.5 COMSOL Simulation

COMSOL Multiphysics v6.0 was employed to simulate the transient temperature evolution on the sample surface inside ceramic wells [10]. The simulation was carried out by coupling the "heat transfer in solids" and "surface-to-surface radiation" modules for time-dependent analysis. The primary heat transfer mechanisms considered in this model were radiation from the carbon paper heater and solid-to-solid conduction via physical contact. Heat transfer via gas convection was assumed to be negligible and was therefore excluded. The model consisted of a carbon paper heater, a ceramic orifice plate, and the precursor-loaded CA-CNFs samples housed within the wells. The initial surface temperature of the sample was set to 298 K, and the heating duration was set to 2.0 s, with temperature outputs recorded at 0.1 s intervals. The average temperatures of both the carbon paper heater and the sample surface were obtained by integration of the computed results. Radiative heating was applied via a constant heat source (1000 W) originating from the carbon paper heater. The radiation heat transfer from the heater to the sample surface and its surroundings was described by Equation S1, while the solid-to-solid conduction was modeled using Equation S2.

$$-\boldsymbol{n}\cdot(-k\nabla T) = h(T_{inf} - T) + \frac{\varepsilon}{1-\varepsilon}(J_0 - \sigma T^4) \qquad \text{(S1)}$$

$$\rho C_p \frac{\partial T}{\partial t} + \nabla\cdot(-k\nabla T) = Q \qquad \text{(S2)}$$

Here, $\boldsymbol{n}$ represents the surface normal vector, k denotes the thermal conductivity, T_{inf} denotes the temperature of the convective cooling gas, T is the initial temperature of the sample surface (298 K), h is the convective heat transfer coefficient, ε is the emissivity of the heater surface, J_0 is the expression for surface radiative heat flux, σ is the Stefan-Boltzmann constant, ρ is the density, C_p is the heat capacity, and Q is the volumetric heat source.

S1.6 Physicochemical Characterization

The crystalline structures of the catalysts were characterized by X-ray diffraction (XRD), using a Bruker D8 ADVANCE diffractometer equipped with a Cu Kα radiation source (λ = 1.54 Å). The morphology and element composition of the catalysts were analyzed by scanning electron microscopy (SEM) and energy-dispersive X-ray spectroscopy (EDS) using a Zeiss Gemini Sigma 300 system. SEM images were acquired at an accelerating voltage of 10.0 kV, while EDS analysis was performed at 15.0 kV. Transmission electron microscopy (TEM) and dark-field EDS mappings were carried out using a Thermo Fisher Scientific Talos F200X microscope equipped with a Super-X EDS detector, operated at an accelerating voltage of 200.0 kV. The surface chemical composition and elemental valence states of the catalysts were analyzed by X-ray photoelectron spectroscopy (XPS) on a Thermo Fisher Scientific K-Alpha system. During XPS measurements, the vacuum was maintained at 5×10^{-10} Pa. The excitation source was Al Kα radiation (hν = 1486.68 eV), with a working voltage of 15.0 kV and filament current of 10 mA. All binding energies were calibrated using the C1s peak at 284.80 eV as a reference. The element compositions of the HEA catalysts were determined by inductively coupled plasma optical emission spectroscopy (ICP-OES) using a Thermo Fisher Scientific iCAP6300 instrument. Samples were digested using aqua regia and diluted with 2% HCl prior to analysis.

S1.7 Electrochemical Evaluation

The ORR performance of 100 HEA catalysts was evaluated in batch using a CHI 900 bipotentiostat equipped with a rotating ring-disk electrode (RRDE, Pine Inc.). All experiments were conducted in 0.1 M $HClO_4$ electrolyte, with a glassy carbon disk RRDE as the working electrode, a graphite rod as the counter electrode, and a saturated calomel electrode (SCE) as the reference. The working electrode was prepared as follows: 5 mg of catalyst was dispersed in 1 mL of mixed solution composed of 950 μL isopropanol and 50 μL Nafion 117 solution (5 wt.%). The preparation of all 100 catalyst inks was automated using the high-throughput liquid dispensing workstation, followed by 1 hour of ultrasonication to ensure homogeneous dispersion. The catalyst ink was then uniformly

drop-cast onto the polished surface of the glassy carbon disk of the RRDE and allowed to dry naturally to form a uniform catalyst film. The total catalyst loading was fixed at 300 μg cm^{-2} for all samples, including the commercial Pt/C control. Prior to electrochemical activity measurements, all electrodes underwent cyclic voltammetry (CV) pretreatment with 40–50 cycles in the potential of 0-1.2 V (*vs* RHE) at a scan rate of 0.5 V s^{-1}, in order to remove any surface contaminants. Accelerated degradation testing (ADT) was conducted by cycling between +0.6 and 1.0 V (*vs* RHE) for 5,000 and 10,000 cycles at a scan rate of 100 mV s^{-1}.

The electron transfer number (n) and H_2O_2 yield of the 100 samples were calculated using the following equations:

$$H_2O_2\% = 200 \times \frac{I_R/N}{I_D + \frac{I_R}{N}} \quad \text{(S3)}$$

$$n = 4 \times \frac{I_D}{I_D + \frac{I_R}{N}} \quad \text{(S4)}$$

where I_R and I_D represent the ring and disk currents, respectively, and N is the collection efficiency, set at 0.37.

For the top three performing samples, the electron transfer n was further determined using the koutecky-levich (K-L) equation:

$$\frac{1}{j} = \frac{1}{j_k} + \frac{1}{B\omega^{1/2}} \quad \text{(S5)}$$

$$B = 0.2nFD_0^{2/3}v^{-1/6}C_0 \quad \text{(S6)}$$

where j_k is the kinetic current density, ω is the rotation rate (r min^{-1}), D_o is the diffusion coefficient of O_2 (1.9×10^{-5} cm^3 s^{-1}), F is the faraday constant (96485 C mol^{-1}), v is the kinematic viscosity of 0.1 M $HClO_4$ electrolyte (0.01 cm^2 s^{-1}), and C_o is the saturated concentration of O_2 at room temperature (1.2×10^{-6} mol cm^{-3}).

S1.8 PEMFC Testing

The membrane electrode assembly (MEA) of the proton exchange membrane fuel cell (PEMFC) was prepared using the catalyst-coated membrane (CCM) method. The detailed procedure is as follows: First, the cathode catalyst FeCoCuPtIr and the commercial Pt/C catalyst (Johnson Matthey, HISPEC3000, 20 wt.%) were mixed at an ionomer-to-carbon ratio of 0.6. Isopropanol was then added, and the mixture was ultrasonicated for 1 h to obtain a homogeneous catalyst ink with a concentration of 1.5-2 mg·mL^{-1}. The resulting dispersion was directly sprayed onto the surface of a Nafion membrane (Gore, ~15 μm) to

form the cathode catalytic layer. For the anode, commercial Pt/C (Johnson Matthey, HISPEC4000, 40 wt.%) was used as the catalyst. The catalyst ink was prepared using the same procedure and sprayed onto the opposite side of the membrane to form the anode catalytic layer. The resulting MEA had an active area of 5 cm^2, with a noble metal loading of 0.1 $mg\cdot cm^{-2}$ on the anode, and 0.05 $mg\cdot cm^{-2}$ (FeCoCuPtIr) or 0.1 $mg\cdot cm^{-2}$ (Pt/C, 20 wt.%) on the cathode. During MEA assembly, gas diffusion layers (GDL, SGL Carbon, thickness ~235 μm) with a microporous layer were used and hot-pressed together with the gasket and CCM to form the complete MEA. PEMFC performance tests were carried out at 80 °C under a back pressure of 150 kPaabs using a PEMFC test system (RG12100, Dalian Ruige Technology Co., Ltd.). During the test, H_2 and air were supplied to the anode and cathode, respectively, at flow rates of 1.2 $L\cdot min^{-1}$ and 0.6 $L\cdot min^{-1}$, with a relative humidity (RH) of 100%.

S1.9 Correlation Analysis

Correlation coefficients are important statistical metrics used to quantify the strength and direction of relationships between different variables. By calculating correlation coefficients, one can assess the extent to which changes in one variable are associated with changes in another. To investigate the influence of noble and non-noble metal element systems in HEA combinations on the half-wave potential ($E_{1/2}$), a series of correlation analysis algorithms were developed with the assistance of the ChatHEA assistant. These tools enabled both quantitative analysis and visual representation of the correlations between HEA element systems and ORR activity.

The pearson correlation coefficient is a commonly used statistical measure that evaluates the linear relationship between two continuous variables. Its value ranges from -1 to +1, where +1 indicates a perfect positive correlation, -1 indicates a perfect negative correlation, and 0 indicates no correlation. The coefficient is calculated using the following formula:

$$r = \frac{\sum_{i=1}^{n}(x_i - \bar{x})(y_i - \bar{y})}{\sqrt{\sum_{i=1}^{n}(x_i - \bar{x})^2 \sum_{i=1}^{n}(y_i - \bar{y})^2} \qquad \text{(S7)}$$

where r denotes the pearson correlation coefficient, n is the number of samples, x_i and y_i represent the i-th observations of variables X and Y, respectively, and $\bar{x}$ and $\bar{y}$ are the sample means of X and Y. The formula is given as:

$$\bar{x} = \frac{\sum_{i=1}^{n} x_i}{n} \qquad \text{(S8)}$$

$$\bar{y} = \frac{\sum_{i=1}^{n} y_i}{n} \qquad \text{(S9)}$$

In the correlation analysis, considering that a single algorithm may not be sufficient to reliably assess correlation results, we further introduced two ML models with the assistance of the ChatHEA assistant for validation and comparison. In the field of ML, regression models serve as important tools for analyzing relationships between variables and assessing feature importance. We selected Lasso regression and Ridge regression, both of which are based on linear regression frameworks and incorporate regularization terms to control model complexity. Specifically, Lasso regression employs L1 regularization to constrain the regression coefficients, while Ridge regression uses L2 regularization for the same purpose.

To further validate the robustness of the correlations identified by linear algorithms and to capture potential nonlinear synergistic effects, two nonlinear models—Random Forest and XGBoost—were subsequently introduced. Random Forest is an ensemble learning method that constructs a large number of decision trees during training and averages their outputs, thereby enhancing model generalization and noise resistance. Its built-in feature importance metric provides a non-parametric measure of each feature's contribution to the prediction target. XGBoost, another powerful ensemble technique based on the gradient boosting algorithm, builds decision trees sequentially, with each new tree correcting the residuals of the previous ones to improve overall prediction accuracy. The feature importance in XGBoost is quantified by the average gain associated with all split points using each feature, revealing which features are most critical for accurate predictions. By integrating the results from both linear and nonlinear models, we achieved a more comprehensive and reliable validation of the correlations and potential nonlinear synergies between elemental systems and catalytic activity.

S1.10 Computational Methods

To reduce the technical barrier for experimental researchers performing density functional theory (DFT) calculations, an initial computational workflow was developed with the assistance of the ChatHEA assistant. This workflow includes structure modeling and optimization, adsorption site selection, and free energy calculations. It serves as a practical guideline for non-specialist users, significantly improving computational efficiency and reducing resource consumption. In terms of modeling strategy, this study adopts a widely accepted mainstream approach in the current field of HEA electrocatalysis[11-15], in which an idealized FCC-(111) clean surface is employed as the base model, and high-entropy characteristics are introduced through random atomic substitution to construct an HEA surface structure with atomic-level disorder[16-18]. To more accurately approximate the electrochemical interface, implicit solvation treatment and a dipole correction along the

surface normal were incorporated into the slab calculations to eliminate artificial electric fields arising from surface asymmetry under periodic boundary conditions. Although this strategy does not explicitly include solvent molecules, electrolyte ions, or pre-adsorbed species, it has been extensively validated in numerous high-impact studies, demonstrating its effectiveness in capturing the statistical structural features and adsorption behavior distributions of HEA surfaces[1, 12-13, 16-17, 19]. Given the inherently high atomic disorder and diverse active sites characteristic of HEA systems, introducing additional pre-adsorbed intermediates such as O* and OH* would lead to complex coupled effects, including adsorption configuration uncertainty, cooperative or competitive adsorption interactions, and coverage-dependent behaviors, thereby markedly increasing model complexity and causing the computational cost to grow exponentially[1, 12-13, 17]. Therefore, based on a thorough survey of literature practices and modeling consensus, this study adopts this broadly accepted modeling scheme in electrocatalysis. By evaluating the adsorption performance of multiple representative active sites, the model enables the elucidation of the structure–activity relationships of HEA surfaces during the ORR process, providing trend-level guidance for experimental design and mechanistic investigation.

All first-principles calculations were performed using the Vienna Ab initio Simulation Package (VASP)[20], with the revised Perdew-Burke-Ernzerhof (RPBE) exchange–correlation functional[21]. The plane-wave cutoff energy was set to 520 eV, and all calculations were conducted with spin polarization enabled (ISPIN=2). To ensure the stability of the magnetic ground state, high-spin initial magnetic moments were assigned to 3d magnetic elements (Mn: 5.0 μ_B, Fe: 4.0 μ_B, Co: 3.0 μ_B, Ni: 2.0 μ_B), while 1.0 μ_B was assigned to Cu and noble metals (Pt, Ir, Pd, Rh) to describe induced magnetism. All magnetic moments were allowed to relax self-consistently with the electronic structure during geometry optimization, ensuring full convergence of both ionic and electronic degrees of freedom. Considering that HEA surfaces exhibit metallic character and weak electronic correlations, and that the focus of this study lies in comparing relative adsorption energies and trends among different systems rather than quantitatively describing strongly correlated electronic behavior, the DFT+U correction was not applied[1, 12, 14, 19, 22-23]. The HEA bulk models were constructed based on the experimentally obtained XRD patterns and elemental molar ratios, followed by random atomic substitution and full structural optimization. The optimized bulk structures were then cleaved along the (111) surface to construct surface slab models consisting of four atomic layers with 16 atoms in total. During optimization, the bottom two layers were fixed, while the top two layers were fully relaxed. All surface models adopted symmetric periodic slab configurations, with a

vacuum layer larger than 15 Å introduced to prevent interlayer interactions. The convergence thresholds were set to 10^{-5} eV for total energy changes and 0.02 eV $Å^{-1}$ for atomic forces. Brillouin zone sampling was performed using a Γ-centered Monkhorst-Pack grid (3 × 3 × 1) [24]. Representative active sites were selected based on the ORR volcano relationship, identified through evaluation of oxygen adsorption energies (ΔE_O), and subsequently used for reaction energy calculations[25]. The relative energies of key reaction intermediates (O_2*, OOH*, O*, and OH*) were determined by identifying the most stable adsorption configurations near each selected active site[12].

The reaction free energy was calculated using the following equation:

$$\Delta G = \Delta E + \Delta ZPE - T\Delta S \qquad \text{(S10)}$$

where ΔE is the reaction energy, defined as the total energy difference between the reactants and products adsorbed on the catalyst surface. ΔZPE is the zero-point energy correction to the Gibbs free energy, T is the temperature (K), and ΔS is the entropy change associated with each reaction step.

Under an applied potential U, the change in free energy is calculated as:

$$\Delta G(U) = \Delta G + eU \qquad \text{(S11)}$$

where U is the applied potential, and e is the elementary charge.

We have developed accurate microkinetic models that effectively capture the pH-dependent ORR catalytic behavior on the RHE scale, demonstrating strong agreement with both high-throughput experimental results and scaling relationships. All relevant computational structures and experimental data are available on the Digital Catalysis Platform (DigCat): www.digcat.org.

Section S2. Supplementary Figures and Tables

ORR-Literatue Text Mining Prompt Engineering : Sequence and Content

Role	Content
System	You are an expert in electrocatalysis literature analysis.
User	Electrocatalyst materials are functional materials that lower energy barriers in electrochemical reactions, accelerate charge transfer, and enhance energy conversion efficiency. They are widely used in fuel cells, water splitting, and CO_2 reduction.
User	The oxygen reduction reaction (ORR) is an electrocatalytic process in which oxygen (O_2) is reduced to water (H_2O) or hydrogen peroxide (H_2O_2) through multi-electron transfer at the catalyst surface.
User	Research article (Research): A primary study presenting original experimental or theoretical results, including methods, data, and analysis.<br>Review article (Review): A secondary study that summarizes, analyzes, and discusses existing research progress, without presenting substantial new experimental data.
User	This is an Abstract from an article about electrocatalyst, analysis this abstract with title, fill the chart, all section should be fill, if you are uncertain about specify items, fill NULL.
User	Please check your answer, read the abstract and title again and reply again.
User	Your reply must be strictly standardized as the following questions:<br>Article research on electrocatalyst materials : Yes or No<br>Article research on ORR : Yes or No<br>Article Type: Research or Review<br>Metal Elements: 1, 2, 3, etc.
User	To be noted, if you are unsure, please reply 'NULL', and the elements should be symbolized with English abbreviation only, for example, if the elements are Iron, Cobalt, Nickel, please reply 'Fe, Co, Ni'

Fig. S1. Prompt engineering strategy based on python scripts calling the GPT-4o API for efficiently mining ORR-related literature and accurately constructing the ORR element library. Notably, the customized prompts markedly enhanced text-mining accuracy and effectively mitigated potential GPT hallucinations.

HEAs-Literatue Text Mining (Sample Case)

Title:
In-situ synthesis of oxygen-enriched carbonaceous layer wrapped with PtNiCo nanoparticles as versatile electrocatalyst

Abstract:
Developing high-performance Pt-based electrocatalysts via the functionalization of carbon carriers is an enormous challenge for their versatile electrocatalytic applications. Herein, carbonaceous (CA) supporter enriched with oxygen-species and anchored by PtNiCo nanoparticles is in-situ synthesized by a liquid-phase calcination (LPC) approach. As a result, PtNiCo/CA obtained in polyethylene glycol presents excellent current density in methanol oxidation reaction (MOR) (435 mA mg^{-1} Pt), higher half-wave potential (0.92 V) in oxygen reduction reaction (ORR), and lower overpotential (377 mV) at the current density of 100 mA cm^{-2} in oxygen evolution reaction (OER). Furthermore, the prepared electrocatalyst shows excellent durability in MOR, ORR, and OER processes. Characterizations including in-situ infrared spectroscopy indicated that the formed M-O species and oxygen-enriched carbonaceous support is conducive to the protonation and deprotonation behaviors, thus enhancing the activity and stability of Pt active sites. Our synthetic strategy shows a potential approach for preparing high-active and long-durable Pt-based versatile electrocatalysts.

ChatGPT-4o:
Article research on electrocatalyst materials: Yes
Article research on ORR: Yes
Article Type: Research
Elements: Pt, Ni, Co

Fig. S2. Example of ORR-related literature text mining using the GPT-4o API, with results demonstrating high accuracy.

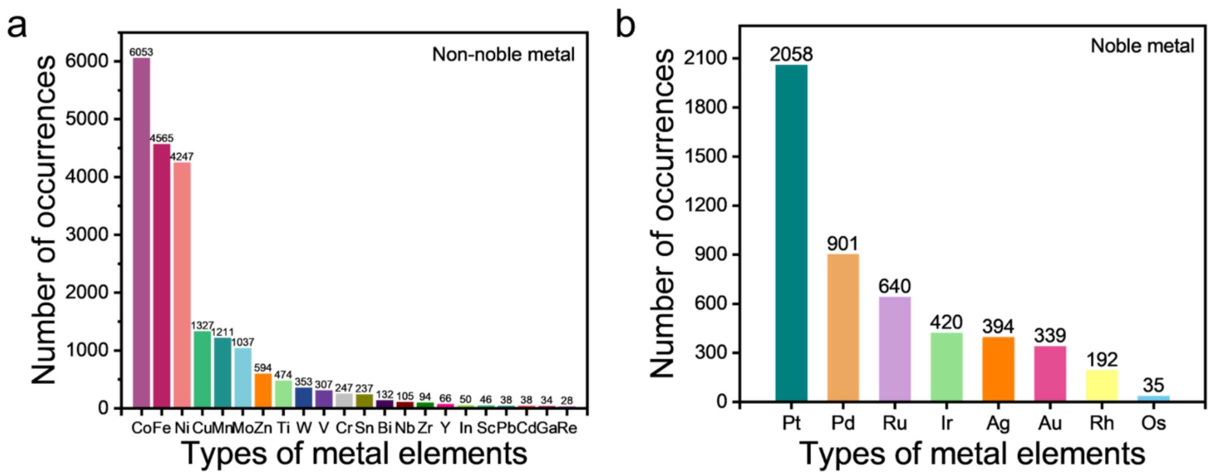

Fig. S3. Statistical distribution of the ORR element library extracted via GPT-4o API text mining: (a) non-noble metals; (b) noble metals.

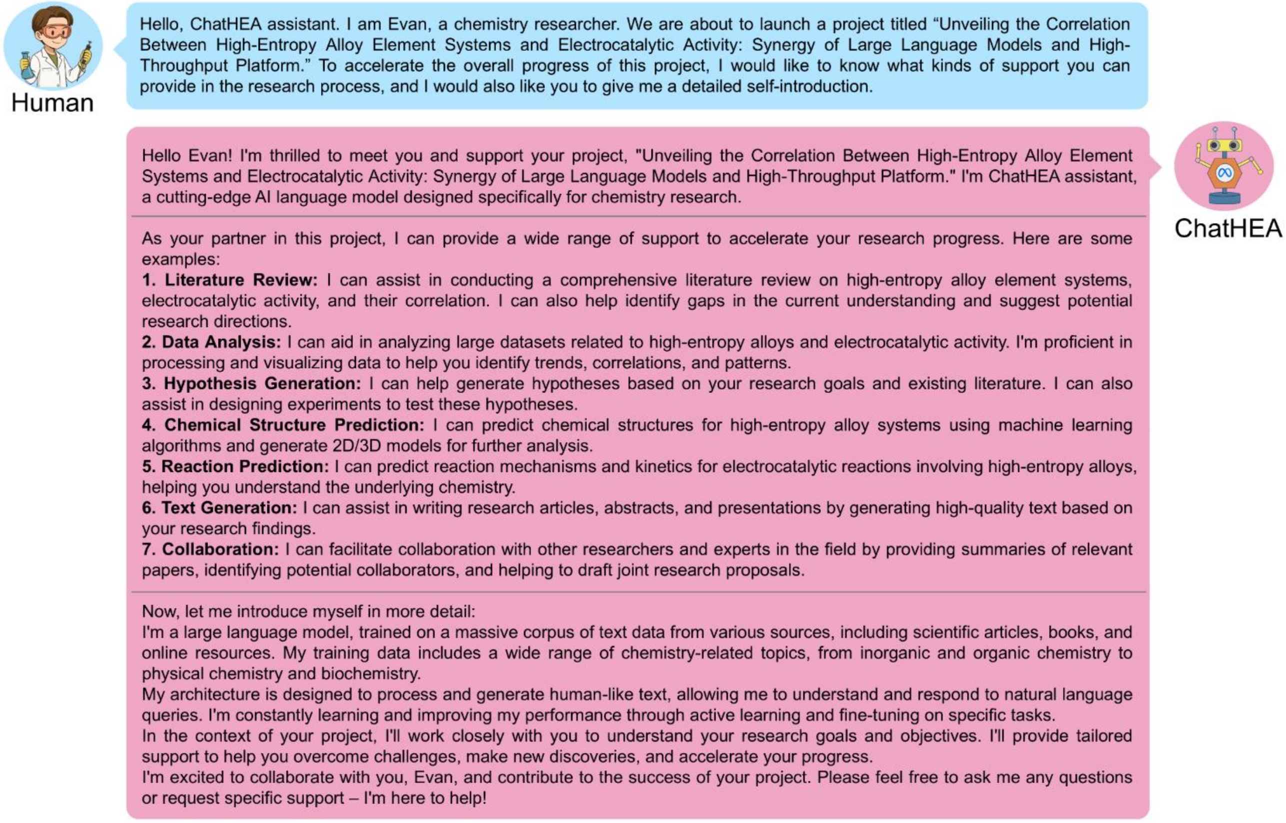

Fig. S4. ChatHEA, an AI assistant for HEA electrocatalysis, was developed by fine-tuning the Meta-Llama-3-8B-Instruct model using the LoRA method within the Llama-Factory framework. It demonstrates comprehensive understanding and in-depth analytical capability in the HEA electrocatalysis domain, enabling human researcher to tackle complex experimental challenges.

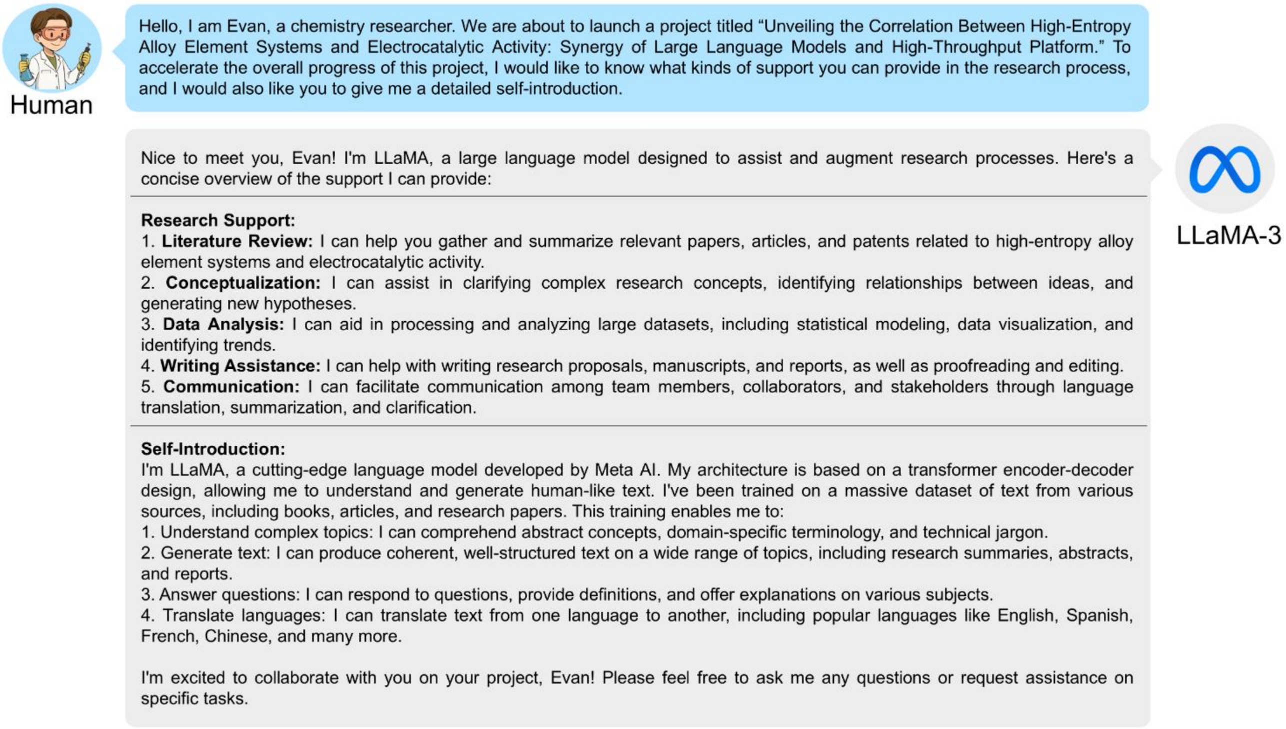

Fig. S5. Prior to domain knowledge fine-tuning, the interactive dialogues between human researchers and the LLaMA-3-8B model demonstrated relatively inadequate capabilities in identity definition and expertise comprehension when compared with the responses generated after LoRA fine-tuning (Fig. S4).

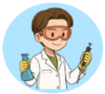
Human

We are now entering the first stage of the project research: the design of quinary high-entropy alloy (HEA) combinations. This step is crucial for uncovering the correlation between element systems and ORR activity. To ensure scientific rigor and practical feasibility, please follow the principles below when recommending the composition schemes:
Please note the following when making recommendations:
1. HEA Composition Rules: Each HEA should consist of five metallic elements, with all elements designed in equiatomic ratios (20 at.%). The selection of HEA elements should fully consider the frequency ranking results obtained during training and be generated through a systematic combinatorial approach.
2. Similarity Requirement: The 100 HEA combinations should include highly similar element systems (e.g., three non-noble metals combined with two noble metals). By fixing certain element pairing patterns (for example, using a combinatorial method: selecting *M* elements from *N* non-noble candidates and *Y* elements from *X* noble candidates, then randomly combining them), the resulting 100 HEA combinations will exhibit systematic variations across the systems. This is essential for subsequent correlation analysis.
3. Chemical Feasibility: Consider the solubility and stability of metal chlorides in ethanol solution, as all precursors are prepared in ethanol phase. Exclude combinations with poor solubility or incompatibility (e.g., AgCl is insoluble in ethanol; Au is incompatible with certain metals during alloy formation).
4. Result Presentation: Please systematically organize the output according to element categories (non-noble *vs.* noble metals) to ensure clarity and facilitate further analysis.
Based on the above standards, please generate 100 HEA combinations and briefly explain the rationale for selecting the core element pool.

LLaMA-3

I'd be happy to help you with that, Evan!
Core Element Pool Selection:
Based on the frequency ranking results obtained during training, I've selected a pool of 15 non-noble metals and 5 noble metals that meet the similarity requirement and chemical feasibility criteria.
The non-noble metal pool includes:
1.Fe, 2.Co, 3.Ni, 4.Cu, 5.Zn, 6.Sn, 7.Pb, 8.Bi, 9.In, 10.Ga, 11.Ge, 12.Sb, 13.As, 14.Se, 15.Te
The noble metal pool includes:
1.Ag, 2.Au, 3.Pt, 4.Pd, 5.Rh

HEA Combinations:
Using the combinatorial approach, I've generated 100 HEA combinations that meet the similarity requirement. Here are the combinations, organized according to element categories:
Non-Noble Metals (NNM) + Noble Metals (NM) Combinations:

- - -BEGIN HEA LIST- - -

Combination #	Metal 1	Metal 2	Metal 3	Metal 4	Metal 5
1	Fe	Co	Ni	Ag	Au
2	Co	Ni	Cu	Zn	Ag
3	Ni	Cu	Zn	Pb	Bi
4	Cu	Zn	Pb	Bi	In
5	Zn	Pb	Bi	In	Ga
6	Pb	Bi	In	Ga	Ge
7	Bi	In	Ga	Ge	Sb
8	In	Ga	Ge	Sb	As
9	Ga	Ge	Sb	As	Se
10	Ge	Sb	As	Se	Te

⋮ **Total of 100 HEA Combinations**

- - -END HEA LIST- - -

Rationale:
The core element pool selection was based on the frequency ranking results, which indicate that these elements are commonly found in high-entropy alloys and exhibit a range of properties that could influence electrocatalytic activity. The combinations were designed to meet the similarity requirement by pairing non-noble metals with noble metals, which is expected to result in systematic variations across the systems. The chemical feasibility criterion was applied to exclude combinations with poor solubility or incompatibility.
Please let me know if you'd like me to elaborate on any aspect of the selection process or if you have any further requests!

Fig. S6. Prior to domain knowledge fine-tuning, the set of 100 HEA combinations enumerated through interactions between human researchers and the LLaMA-3-8B model—when compared to the responses after LoRA fine-tuning (Fig. S8)—did not account for potentially highly active elements in the ORR, nor did they consider issues such as precursor properties of metallic elements and metallic compatibility. This oversight would pose significant challenges for subsequent experimental execution.

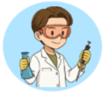

Human

We have completed the design of HEA combinations related to ORR activity correlations. The next step is to clarify the functions of the available laboratory instruments and, based on these, formulate an integrated experimental workflow covering synthesis, preparation, and testing. The specific equipment and descriptions are as follows:

1. High-Throughput Solid/Liquid Dispensing Workstations

The solid dispensing workstation enables automated and precise weighing of metal precursor salts ($FeCl_3 \cdot 6H_2O$, $CoCl_2 \cdot 6H_2O$, $NiCl_2 \cdot 6H_2O$, $CuCl_2 \cdot 6H_2O$, $MnCl_2 \cdot 4H_2O$, $H_2PtCl_6 \cdot 6H_2O$, $PdCl_2$, $RhCl_3 \cdot xH_2O$, $RuCl_3 \cdot xH_2O$, $IrCl_4 \cdot xH_2O$), ensuring accurate compositional ratios.

The liquid dispensing workstation is used for automated pipetting and solution preparation. Ethanol is chosen as the solvent to prepare precursor solutions with a concentration of 0.05 M and a volume of 10 mL.

The liquid workstation can also dispense the prepared quinary mixed precursor solutions in set volumes into well plates preloaded with CO_2-activated carbon nanofibers (CA-CNFs) supports.

2. Vacuum Heating and Drying Workstation

Capable of heating from room temperature to 500 K under vacuum, this system is used for drying samples after precursor loading onto supports, thereby removing residual solvents.

3. High-Throughput Material Preparation Workstation

Equipped with controlled high-temperature radiant heating, with a temperature range of 300–3000 K, and operable under vacuum or Ar atmosphere.

All precursor-loaded samples are placed in ceramic well plates, with each plate accommodating 50 samples. Based on prior experience, temperatures ≥1500 K are required to achieve thermal reduction and alloying of HEAs.

4. Rotating Disk Electrode (RDE) Testing Workstation

Used for standardized evaluation of HEA catalyst ORR activity.

The testing conditions are LSV measurements conducted in 0.1 M $HClO_4$ electrolyte at 1600 rpm, in order to obtain the half-wave potentials ($E_{1/2}$) of 100 HEA catalysts, which will form the dataset for subsequent correlation analysis.

Task: Based on the above instrument capabilities, please design a complete experimental workflow covering synthesis–preparation–testing for the systematic study of 100 HEA catalysts, and provide recommended experimental parameters. The experiments will be carried out by me, and the resulting data will be fed back to you to support subsequent data analysis and optimization.

LLaMA-3

Exciting to move on to the next stage, Evan!
Comprehensive Workflow:
Based on the laboratory instruments, I've designed a comprehensive workflow for the synthesis-preparation-testing of 100 sets of HEA catalysts. The workflow is divided into three main stages: Synthesis, Preparation, and Testing.

Stage 1: Synthesis

1. **High-throughput solid dispensing workstation:**
    * Weigh 0.2 g of each solid precursor salt (HEA composition) using the workstation.
    * Repeat for each of the 100 HEA compositions.

2. **High-throughput materials production workstation:**
    * Place the weighed precursor salts in the workstation's batch synthesis chamber.
    * Set the temperature range: 800°C - 1200°C, with a 50°C increment.
    * Synthesize each HEA composition for 30 minutes.
    * Repeat for each of the 100 HEA compositions.

Stage 2: Preparation

1. **High-throughput liquid dispensing workstation:**
    * Prepare 100 mL of ethanol solution (95%) in a separate container.
    * Add 0.1 g of each synthesized HEA composition to the ethanol solution.
    * Stir the mixture for 30 minutes to ensure homogeneity.
    * Repeat for each of the 100 HEA compositions.

Stage 3: Testing

1. Rotating disk electrode testing workstation:
    * Prepare the rotating disk electrode (RDE) with a platinum disk (1 cm^2).
    * Set the testing conditions:
        + Temperature: 25°C
        + pH: 1.0
        + Rotation speed: 1600 rpm
        + Scan rate: 10 mV/s
    * Test each HEA composition's ORR activity using the RDE workstation.
    * Record the polarization curves and calculate the electrochemical performance metrics (e.g., peak current density, onset potential).

Recommended Experimental Parameters:
* Temperature range: 800°C - 1200°C, with a 50°C increment.
* Solution preparation ratio: 0.1 g HEA composition per 100 mL ethanol solution.
* Testing conditions:
    + Temperature: 25°C
    + pH: 1.0
    + Rotation speed: 1600 rpm
    + Scan rate: 10 mV/s

Experimental Plan:
You will synthesize and prepare each of the 100 HEA compositions using the workflow outlined above. The resulting catalysts will be tested using the RDE workstation, and the experimental results will be fed back to me to support subsequent analysis and optimization.
Please let me know if you have any questions or concerns about the workflow or experimental parameters. I'm here to help!

Fig. S7. Prior to domain knowledge fine-tuning, the experimental procedure for exploring 100 HEA combinations, planned through interactions between human researchers and the LLaMA-3-8B model, shows a lack of executable feasibility and contains multiple errors when compared to the responses after LoRA fine-tuning (Fig. S9).

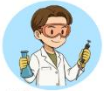

ChatHEA, we are now entering the first stage of the project research: the design of quinary high-entropy alloy (HEA) combinations. This step is crucial for uncovering the correlation between element systems and ORR activity. To ensure scientific rigor and practical feasibility, please follow the principles below when recommending the composition schemes:

Please note the following when making recommendations:

1. HEA Composition Rules: Each HEA should consist of five metallic elements, with all elements designed in equiatomic ratios (20 at.%). The selection of HEA elements should fully consider the frequency ranking results obtained during training and be generated through a systematic combinatorial approach.

2. Similarity Requirement: The 100 HEA combinations should include highly similar element systems (e.g., three non-noble metals combined with two noble metals). By fixing certain element pairing patterns (for example, using a combinatorial method: selecting *M* elements from *N* non-noble candidates and *Y* elements from *X* noble candidates, then randomly combining them), the resulting 100 HEA combinations will exhibit systematic variations across the systems. This is essential for subsequent correlation analysis.

3. Chemical Feasibility: Consider the solubility and stability of metal chlorides in ethanol solution, as all precursors are prepared in ethanol phase. Exclude combinations with poor solubility or incompatibility (e.g., AgCl is insoluble in ethanol; Au is incompatible with certain metals during alloy formation).

4. Result Presentation: Please systematically organize the output according to element categories (non-noble *vs.* noble metals) to ensure clarity and facilitate further analysis.

Based on the above standards, please generate 100 HEA combinations and briefly explain the rationale for selecting the core element pool.

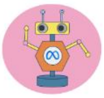

Rationale: We restrict the pools to Non-Noble={Fe, Co, Ni, Cu, Mn} and Noble={Pt, Pd, Ru, Ir, Rh} to balance activity, cost, and stability for ORR in acidic media. Ag is excluded due to AgCl insolubility in ethanol; Au is excluded due to alloying incompatibilities. Each alloy is equiatomic (20 at.%) with 3 Non-Noble + 2 Noble to control precious-metal fraction (~40 at.%) while enabling synergistic pairs (e.g., Co–Ni, Fe–Ni, Fe–Cu) and robust solid-solution formation. Chloride precursors are selected for ethanol-phase processing and compatibility with our automated synthesis pipeline.

- - -BEGIN HEA LIST- - -

Combination #	Metal 1	Metal 2	Metal 3	Metal 4	Metal 5
1	Co	Cu	Mn	Ir	Ru
2	Co	Fe	Mn	Ir	Rh
3	Co	Fe	Mn	Ir	Ru
4	Co	Mn	Ni	Pt	Ru
5	Co	Cu	Fe	Ir	Rh
6	Cu	Fe	Mn	Ir	Pt
7	Co	Mn	Ni	Ir	Pd
8	Cu	Fe	Ni	Ir	Rh
9	Co	Mn	Ni	Pt	Rh
10	Cu	Fe	Mn	Pd	Pt

⋮ **Total of 100 HEA Combinations**

- - -END HEA LIST- - -

Fig. S8. Guided by refined prompt engineering, ChatHEA first defined the candidate element pool for enumerating 100 HEA combinations. By considering precursor physicochemical properties, noble-metal proportions, and element synergy strategies, it generated the final list of HEA combinations. These combinations exhibit high similarity in element systems, which is crucial for subsequent correlation analysis. The complete list of element combinations is provided in Table S1, with sequence numbers rearranged according to element system patterns.

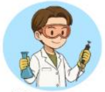

Human

ChatHEA, we have completed the design of HEA combinations related to ORR activity correlations. The next step is to clarify the functions of the available laboratory instruments and, based on these, formulate an integrated experimental workflow covering synthesis, preparation, and testing. The specific equipment and descriptions are as follows:

1. High-Throughput Solid/Liquid Dispensing Workstations

The solid dispensing workstation enables automated and precise weighing of metal precursor salts ($FeCl_3 \cdot 6H_2O$, $CoCl_2 \cdot 6H_2O$, $NiCl_2 \cdot 6H_2O$, $CuCl_2 \cdot 6H_2O$, $MnCl_2 \cdot 4H_2O$, $H_2PtCl_6 \cdot 6H_2O$, $PdCl_2$, $RhCl_3 \cdot xH_2O$, $RuCl_3 \cdot xH_2O$, $IrCl_4 \cdot xH_2O$), ensuring accurate compositional ratios.

The liquid dispensing workstation is used for automated pipetting and solution preparation. Ethanol is chosen as the solvent to prepare precursor solutions with a concentration of 0.05 M and a volume of 10 mL.

The liquid workstation can also dispense the prepared quinary mixed precursor solutions in set volumes into well plates preloaded with CO_2-activated carbon nanofibers (CA-CNFs) supports.

2. Vacuum Heating and Drying Workstation

Capable of heating from room temperature to 500 K under vacuum, this system is used for drying samples after precursor loading onto supports, thereby removing residual solvents.

3. High-Throughput Material Preparation Workstation

Equipped with controlled high-temperature radiant heating, with a temperature range of 300–3000 K, and operable under vacuum or Ar atmosphere.

All precursor-loaded samples are placed in ceramic well plates, with each plate accommodating 50 samples. Based on prior experience, temperatures ≥1500 K are required to achieve thermal reduction and alloying of HEAs.

4. Rotating Disk Electrode (RDE) Testing Workstation

Used for standardized evaluation of HEA catalyst ORR activity.

The testing conditions are LSV measurements conducted in 0.1 M $HClO_4$ electrolyte at 1600 rpm, in order to obtain the half-wave potentials ($E_{1/2}$) of 100 HEA catalysts, which will form the dataset for subsequent correlation analysis.

Task: Based on the above instrument capabilities, please design a complete experimental workflow covering synthesis–preparation–testing for the systematic study of 100 HEA catalysts, and provide recommended experimental parameters. The experiments will be carried out by me, and the resulting data will be fed back to you to support subsequent data analysis and optimization.

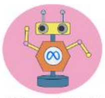

ChatHEA

Based on the available laboratory instruments, I propose the following integrated experimental workflow for the systematic study of 100 HEA catalysts:

Step 1: Synthesis and Preparation

1.1. Solid Dispensing Workstation:

Weigh metal precursor salts ($FeCl_3 \cdot 6H_2O$, $CoCl_2 \cdot 6H_2O$, $NiCl_2 \cdot 6H_2O$, $CuCl_2 \cdot 6H_2O$, $MnCl_2 \cdot 4H_2O$, $H_2PtCl_6 \cdot 6H_2O$, $PdCl_2$, $RhCl_3 \cdot xH_2O$, $RuCl_3 \cdot xH_2O$, $IrCl_4 \cdot xH_2O$) using the solid dispensing workstation to ensure accurate compositional ratios.

Prepare quinary mixed precursor solutions with a concentration of 0.05 M in ethanol (10 mL each) using the liquid dispensing workstation.

1.2. High-Throughput Material Preparation Workstation:

Load the prepared quinary mixed precursor solutions onto CO_2-activated carbon nanofibers (CA-CNFs) supports in ceramic well plates (50 samples per plate).

Dry the precursor-loaded samples in the vacuum heating and drying workstation at 500 K for 2 hours to remove residual solvents.

1.3. High-Throughput Material Preparation Workstation (again):

Place the dried precursor-loaded samples in ceramic well plates and heat them in the high-temperature radiant heating system under vacuum or Ar atmosphere to achieve thermal reduction and alloying of HEAs at temperatures ≥1500 K.

Step 2: Testing

2.1. Rotating Disk Electrode (RDE) Testing Workstation:

Place the prepared HEA catalysts in the RDE testing workstation.

Conduct LSV measurements in 0.1 M $HClO_4$ electrolyte at 1600 rpm to obtain the half-wave potentials ($E_{1/2}$) for each of the 100 HEA catalysts.

Recommended Experimental Parameters:
Precursor concentration: 0.05 M in ethanol
Precursor solution volume: 10 mL
Drying temperature: 500 K
Thermal reduction and alloying temperature: ≥1500 K
Testing conditions: LSV measurements in 0.1 M $HClO_4$ electrolyte at 1600 rpm

Experimental Workflow:

1. Synthesize and prepare the HEA catalysts using the solid and liquid dispensing workstations, followed by drying and thermal reduction/alloying using the vacuum heating and drying workstation and high-temperature radiant heating system, respectively.
2. Load the prepared HEA catalysts into the RDE testing workstation and conduct LSV measurements to evaluate their ORR activity.
3. Collect the half-wave potentials ($E_{1/2}$) for each of the 100 HEA catalysts and feed the resulting data back to support subsequent correlation analysis and optimization.

Please note that the experimental workflow is designed to be high-throughput and efficient, allowing for the systematic study of 100 HEA catalysts. However, it is essential to monitor the quality and consistency of the prepared HEA catalysts and testing results to ensure reliable data for correlation analysis and optimization.

Fig. S9. Leveraging information from the high-throughput experimental platform and various workstations, ChatHEA generated a preliminary experimental workflow for exploring 100 HEA combinations. This workflow encompasses multiple tasks, including HEA catalyst synthesis, electrochemical ORR testing, and data processing. By following the ChatHEA-designed workflow, researcher can substantially reduce the time required for experimental planning.

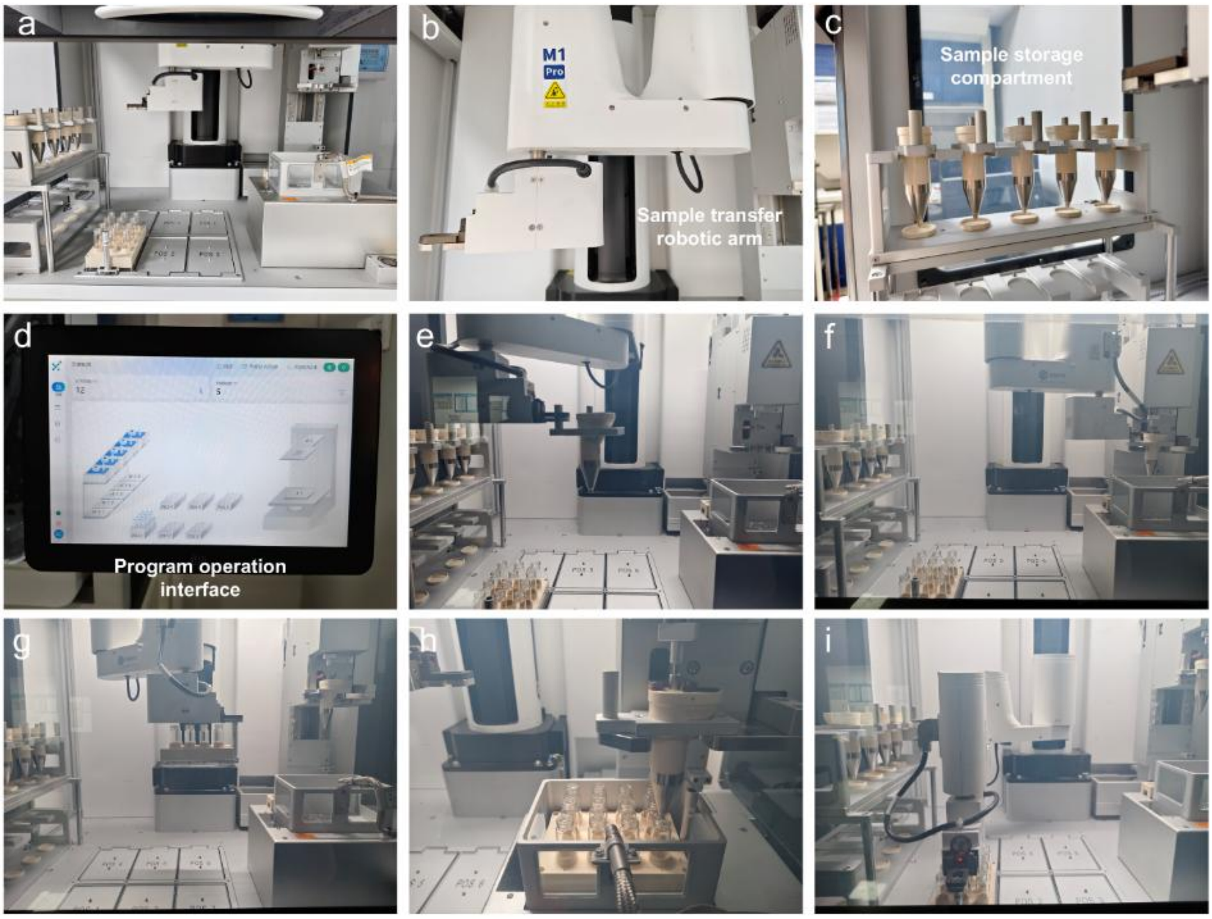

Fig. S10. High-throughput automated dispensing process for solid precursor samples. (a) Digital photograph of the high-throughput solid dispensing workstation; (b) digital photograph of the sample transfer robotic arm; (c) precursor powder storage module; (d) user interface of the solid dispensing program; (e–i) sequential steps of a single automated precursor dispensing cycle.

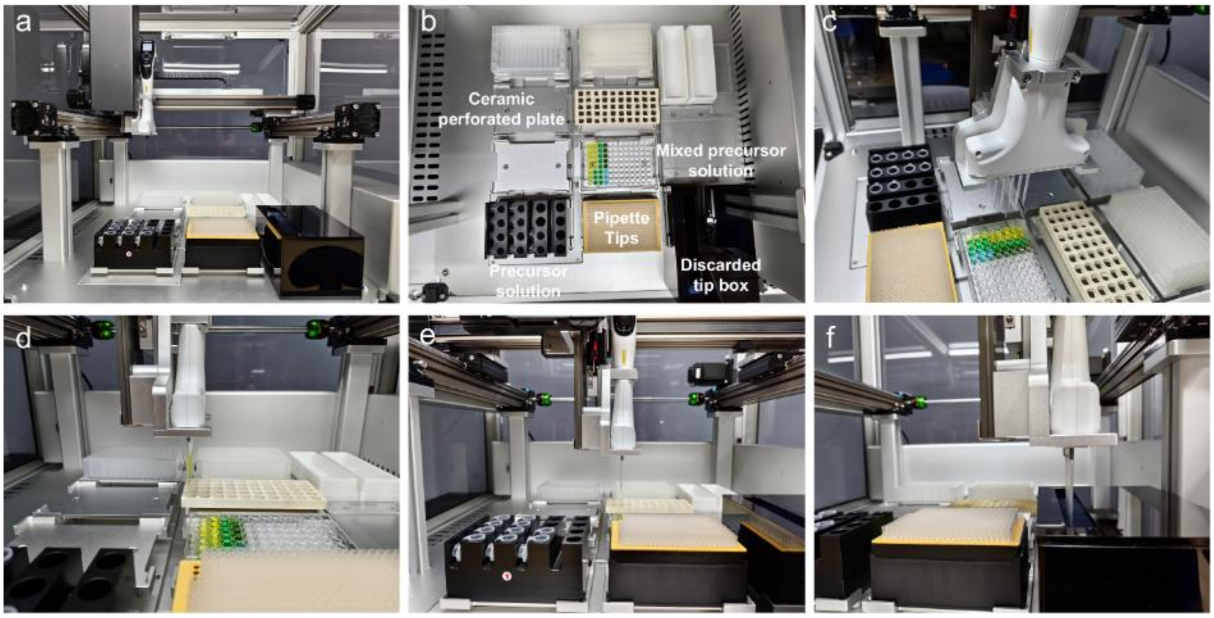

Fig. S11. High-throughput automated preparation and dispensing of mixed precursor solutions. (a) Digital photograph of the high-throughput liquid dispensing workstation; (b) view of the workstation's operational area; (c-e) automated dispensing process of mixed precursor solutions; (f) automated disposal of used pipette tips upon task completion.

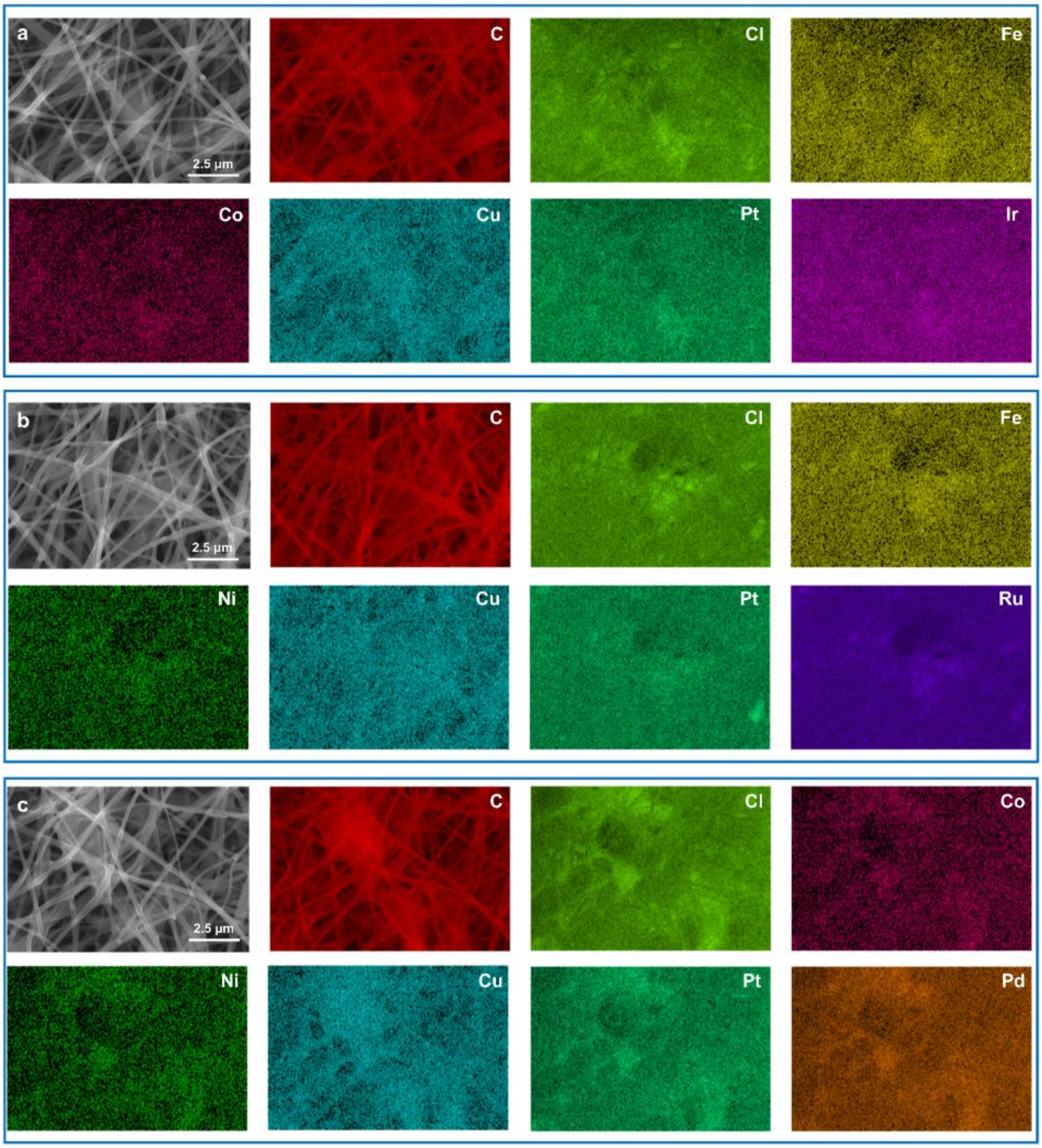

Fig. S12. SEM images and corresponding EDS mappings of quinary metal precursors (a) FeCoCuPtIr, (b) FeNiCuPtRu, and (c) CoNiCuPtPd loaded on CA-CNFs. The results show uniform precursor distributions across the support, providing a solid foundation for the subsequent thermal-radiation synthesis of uniformly dispersed, high-density HEA nanoparticles.

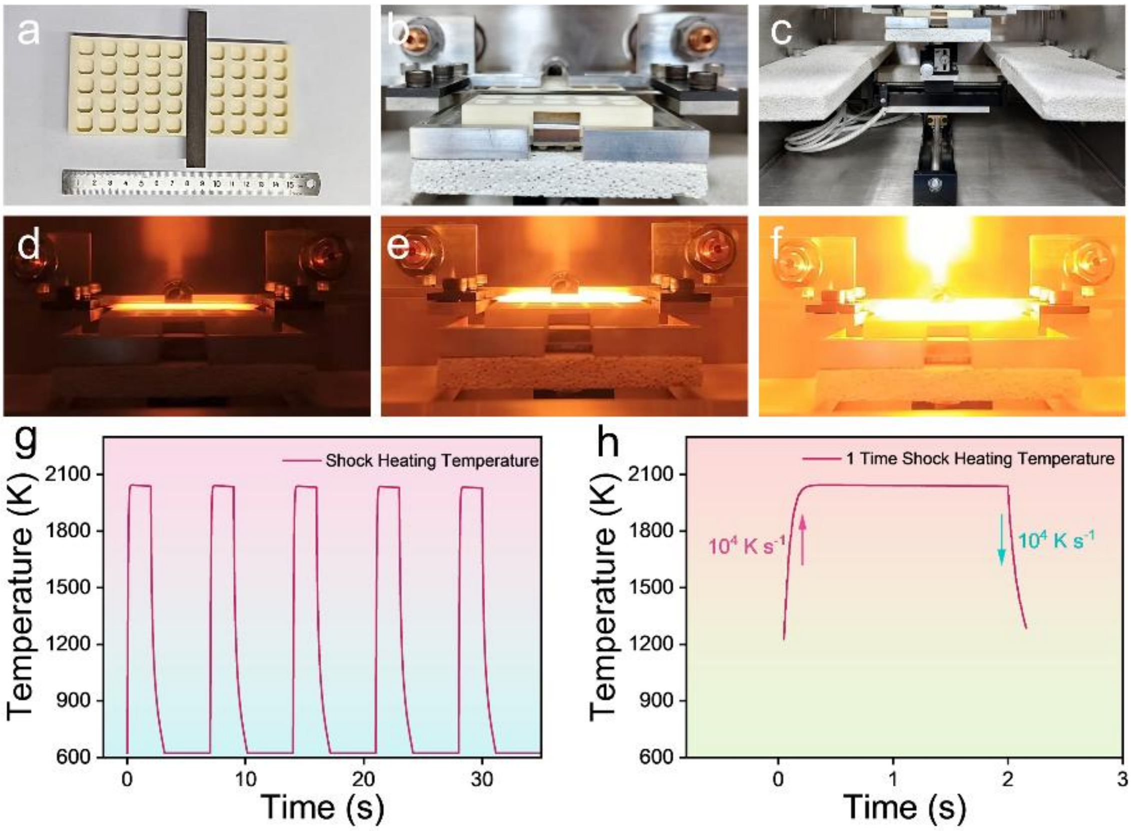

Fig. S13. High-throughput high-temperature radiation heating synthesis process. (a) Digital photograph of the ceramic orifice plate loaded with samples and the heating unit; (b) digital photograph of the radiation heating device; (c) displacement drive system of the ceramic plate; (d-f) digital images of the high-throughput radiation heating process; (g) temperature-time profile of the radiation heating cycles (~2100 K for 5 cycles); (h) temperature profile of a single radiation heating event, highlighting the rapid heating and cooling behavior.

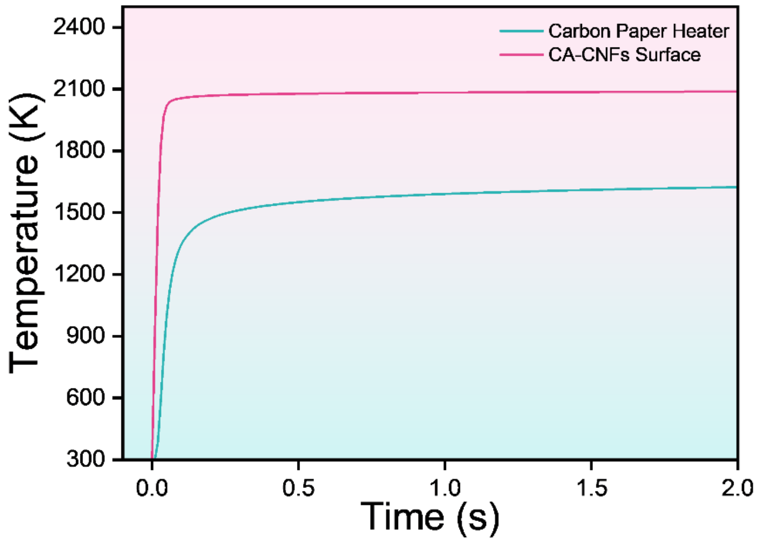

Fig. S14. Temperature evolution curves of the carbon paper heater and the sample surface inside the ceramic wells, obtained via COMSOL simulation.

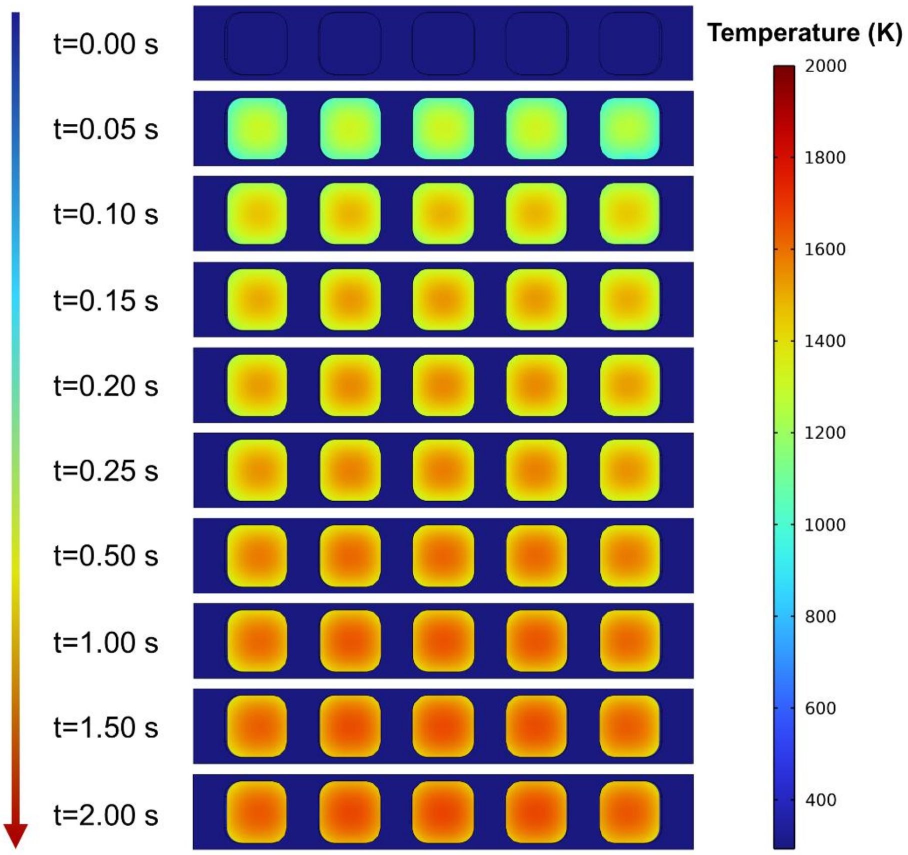

Fig. S15. Transient temperature evolution heatmaps of the sample surface inside the ceramic wells, obtained via COMSOL simulation.

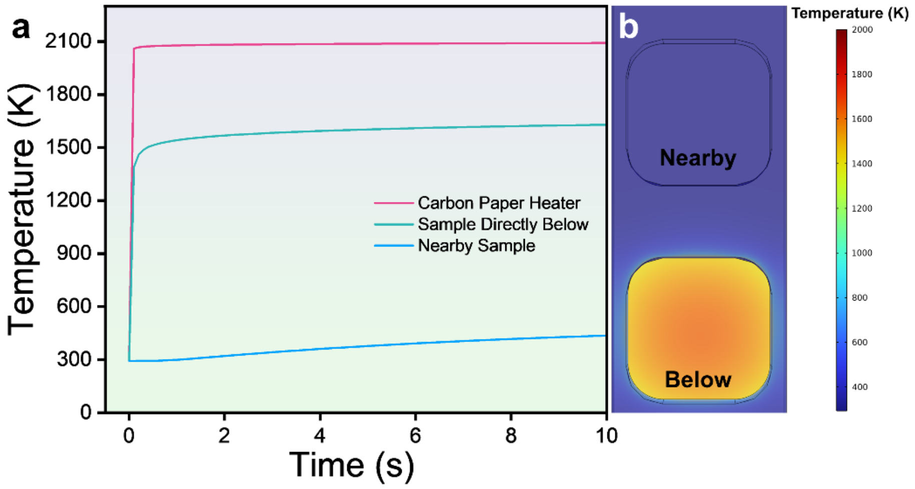

Fig. S16. COMSOL simulation results for adjacent sample surfaces inside the ceramic wells: (a) transient temperature evolution curves and (b) heatmap after 10 s of radiation heating. The extended radiation simulation confirms that sequential row-by-row heating causes minimal thermal interference to neighboring precursor samples, even after 10 s of exposure, the surface temperature in adjacent wells remains as low as ~430 K.

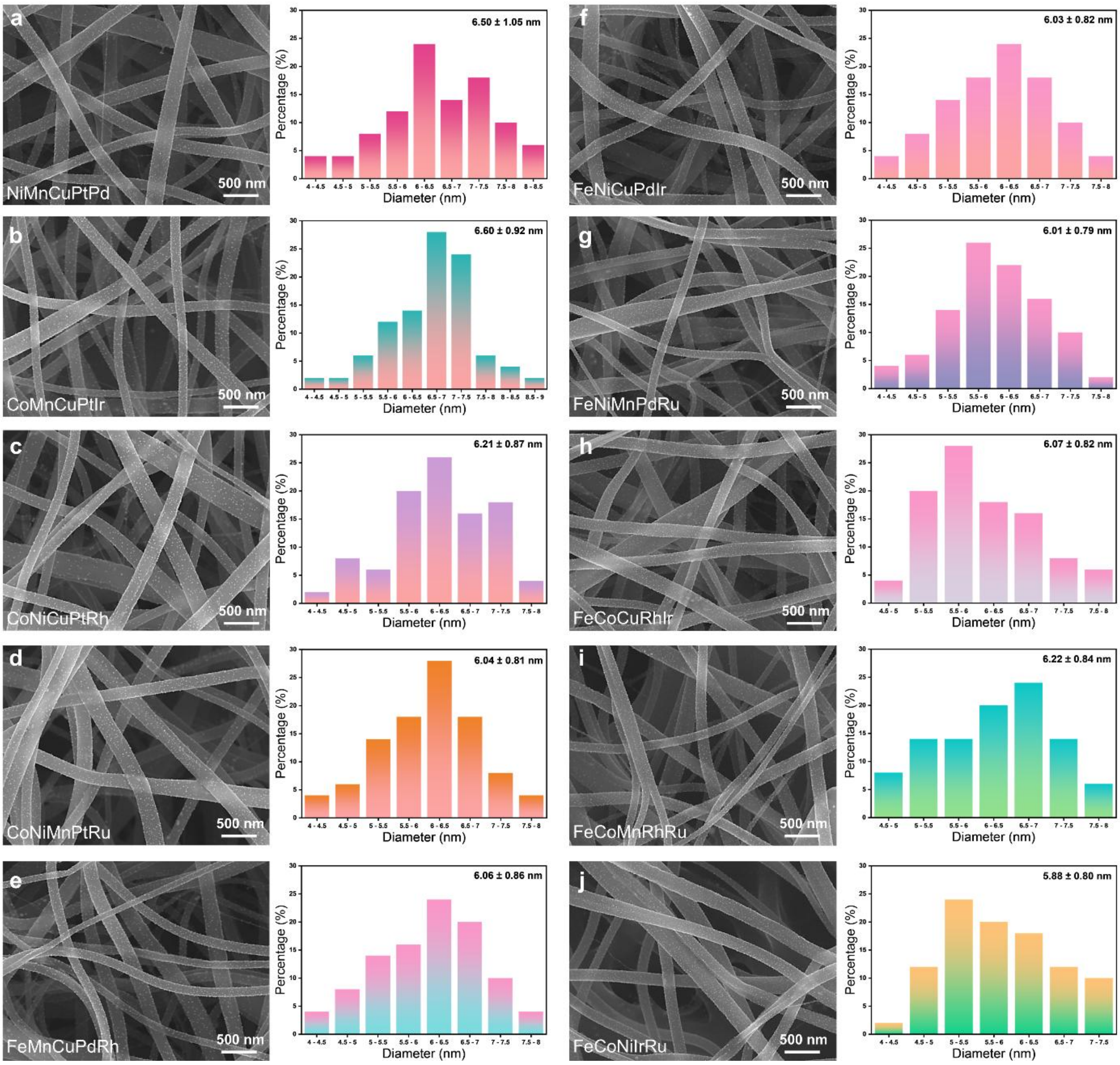

Fig. S17. SEM images of HEAs@CA-CNFs with ten different element combinations, along with corresponding particle size distribution histograms. The results indicate a high loading density of HEA nanoparticles with generally uniform particle sizes.

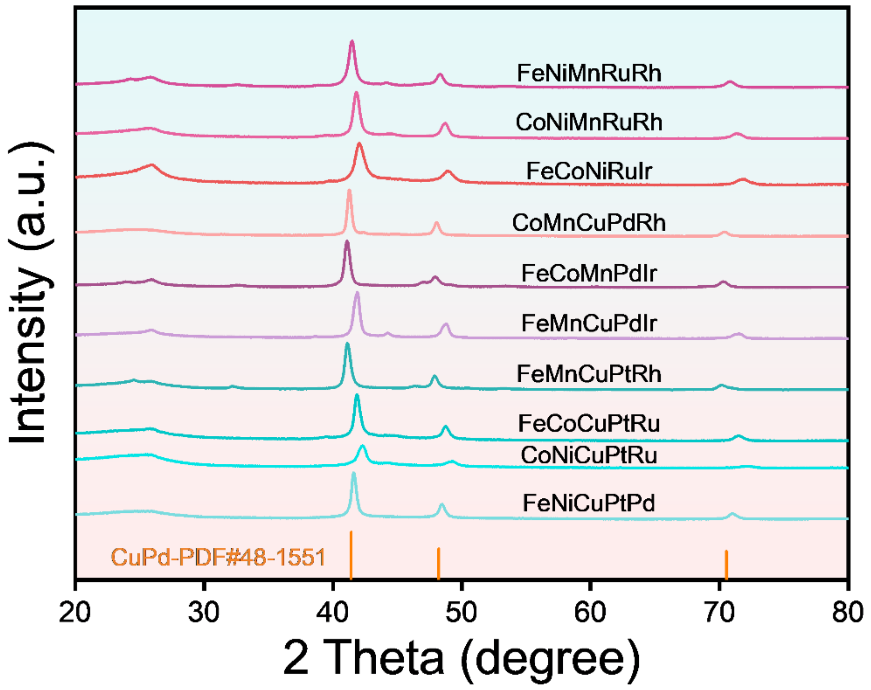

Fig. S18. XRD patterns of HEAs@CA-CNFs with ten different element combinations, showing that all samples exhibit a single-phase FCC structure.

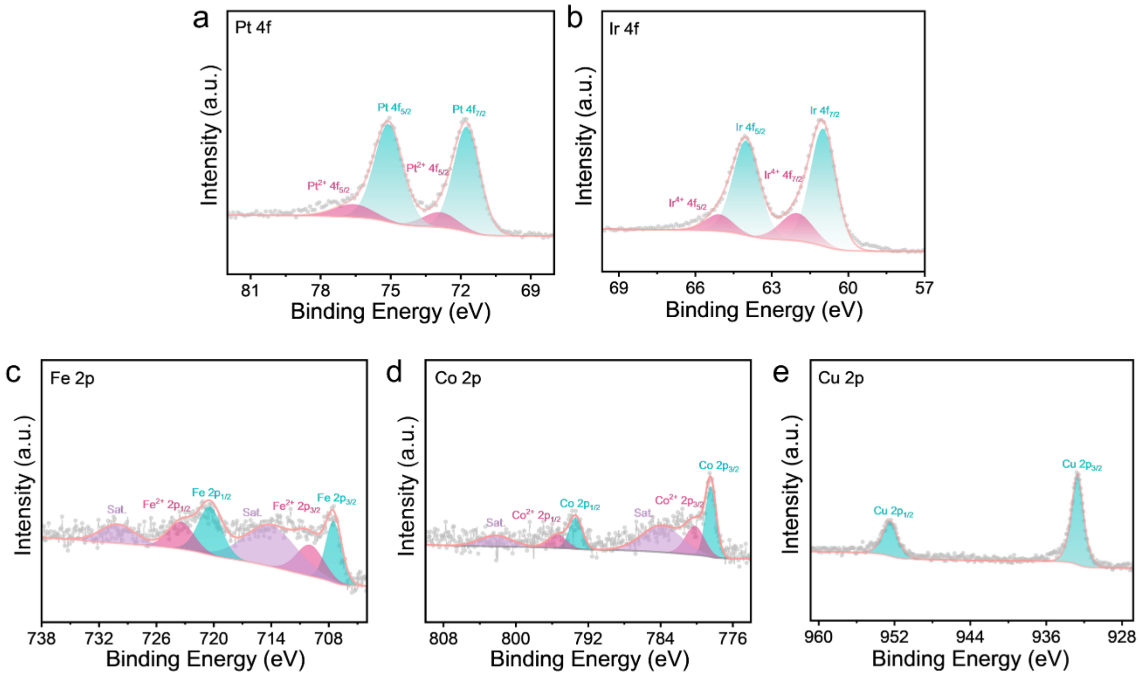

Fig. S19. XPS analysis of FeCoCuPtIr@CA-CNFs. High-resolution spectra for (a) Pt 4f, (b) Ir 4f, (c) Fe 2p, (d) Co 2p, and (e) Cu 2p.

It is worth noting that the entire synthesis process was conducted under an inert gas atmosphere using argon protection. The resulting FeCoCuPtIr@CA-CNFs samples were stored in an argon-filled glovebox prior to physical characterization and electrochemical testing, ensuring that the synthesized materials remained in an unoxidized HEA state. However, unavoidable air exposure during sample preparation prior to XPS measurement led to surface oxidation. Since XPS detects only the surface composition, the presence of metal oxides observed in the high-resolution spectra reflects this surface oxidation rather than the bulk composition.

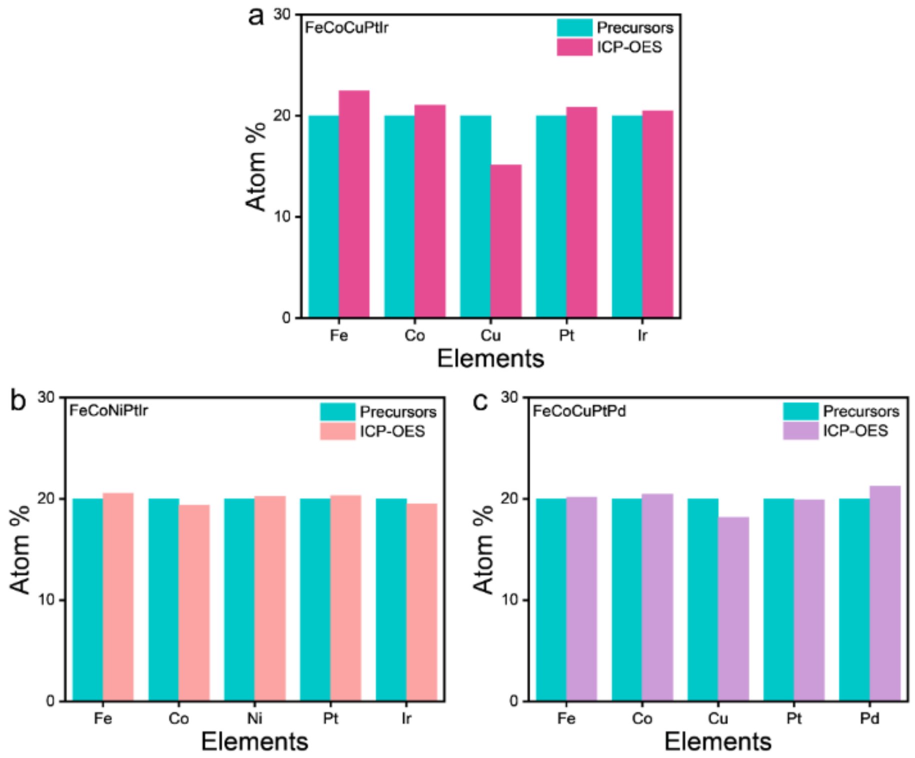

Fig. S20. ICP-OES analysis comparing the actual metal element ratios in HEAs@CA-CNFs with the intended precursor feeding ratios for (a) FeCoCuPtIr, (b) FeCoNiPtIr, and (c) FeCoCuPtPd. The results indicate that the actual element compositions closely match the target ratios, with minor deviations primarily attributed to differences in the vapor pressures of the metal elements during high-temperature processing.

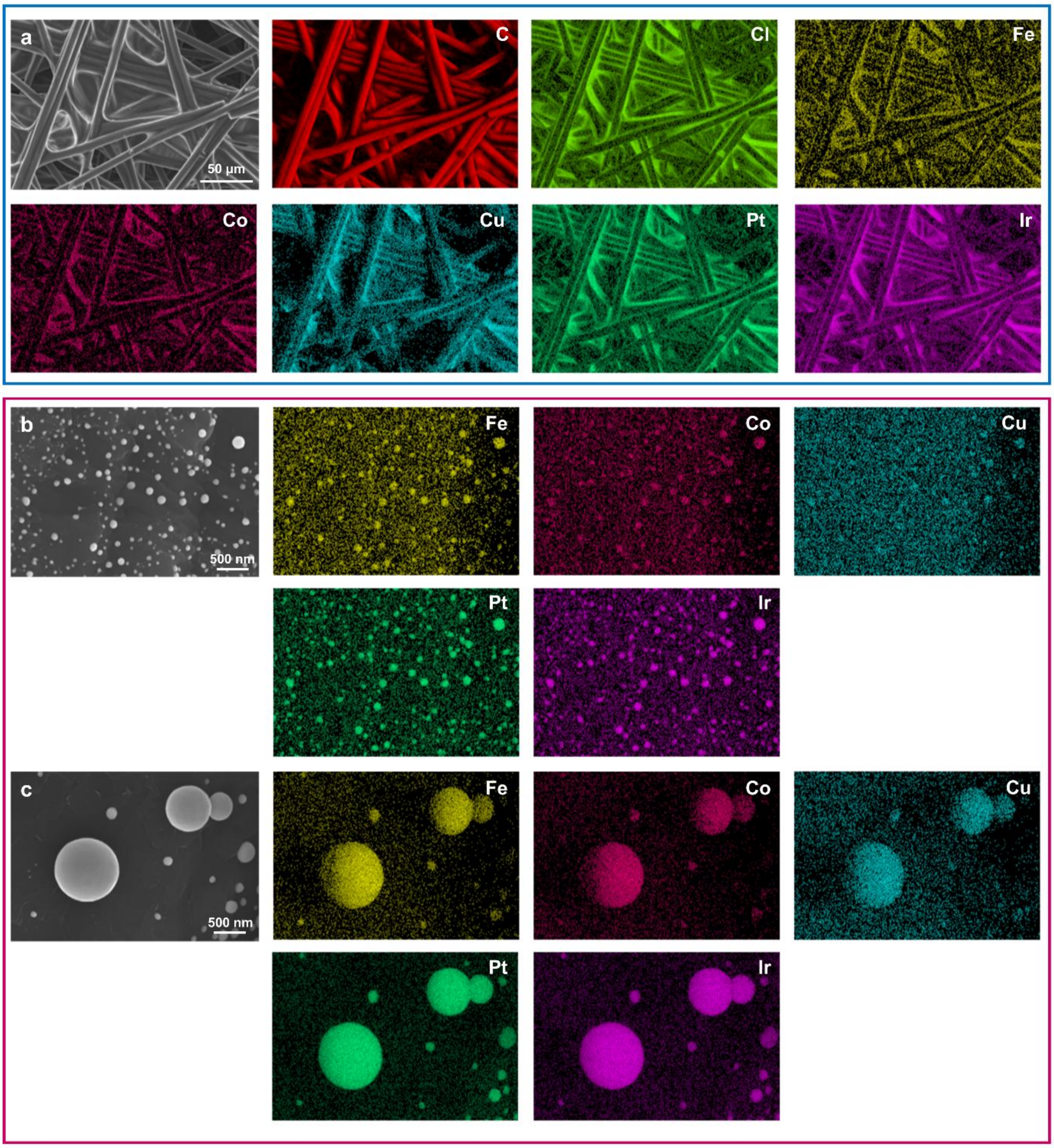

Fig. S21. Scalable synthesis validation of FeCoCuPtIr on a carbon fiber paper (CFP) support. (a) SEM image and corresponding EDS mappings of the quinary metal precursors on CFP, showing uniform distribution across the substrate. (b–c) SEM images and EDS mappings of FeCoCuPtIr@CFP: (b) high-density, size-uniform HEA nanoparticles formed under standard precursor conditions, and (c) larger HEA nanoparticles generated under high-concentration precursor conditions, with increased particle size facilitating EDS analysis.

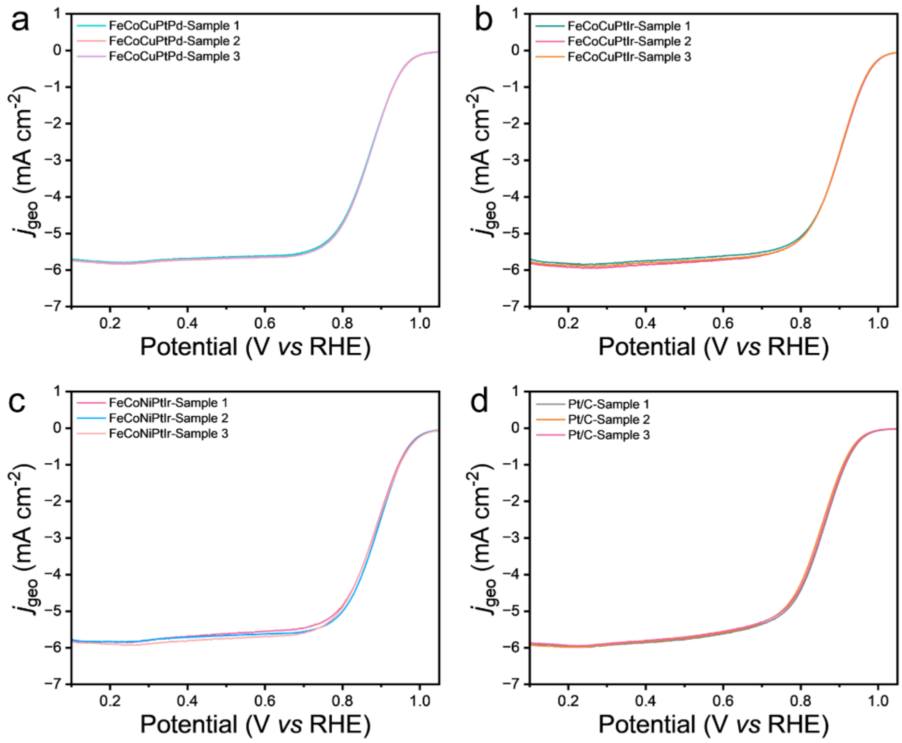

Fig. S22. Reproducibility tests of ORR-LSV curves for multiple parallelly prepared HEA samples and the Pt/C catalyst: (a) FeCoCuPtPd, (b) FeCoCuPtIr, (c) FeCoNiPtIr, and (d) Pt/C. The highly overlapping curves demonstrate the excellent stability and reliability of the synthesis and testing procedures.

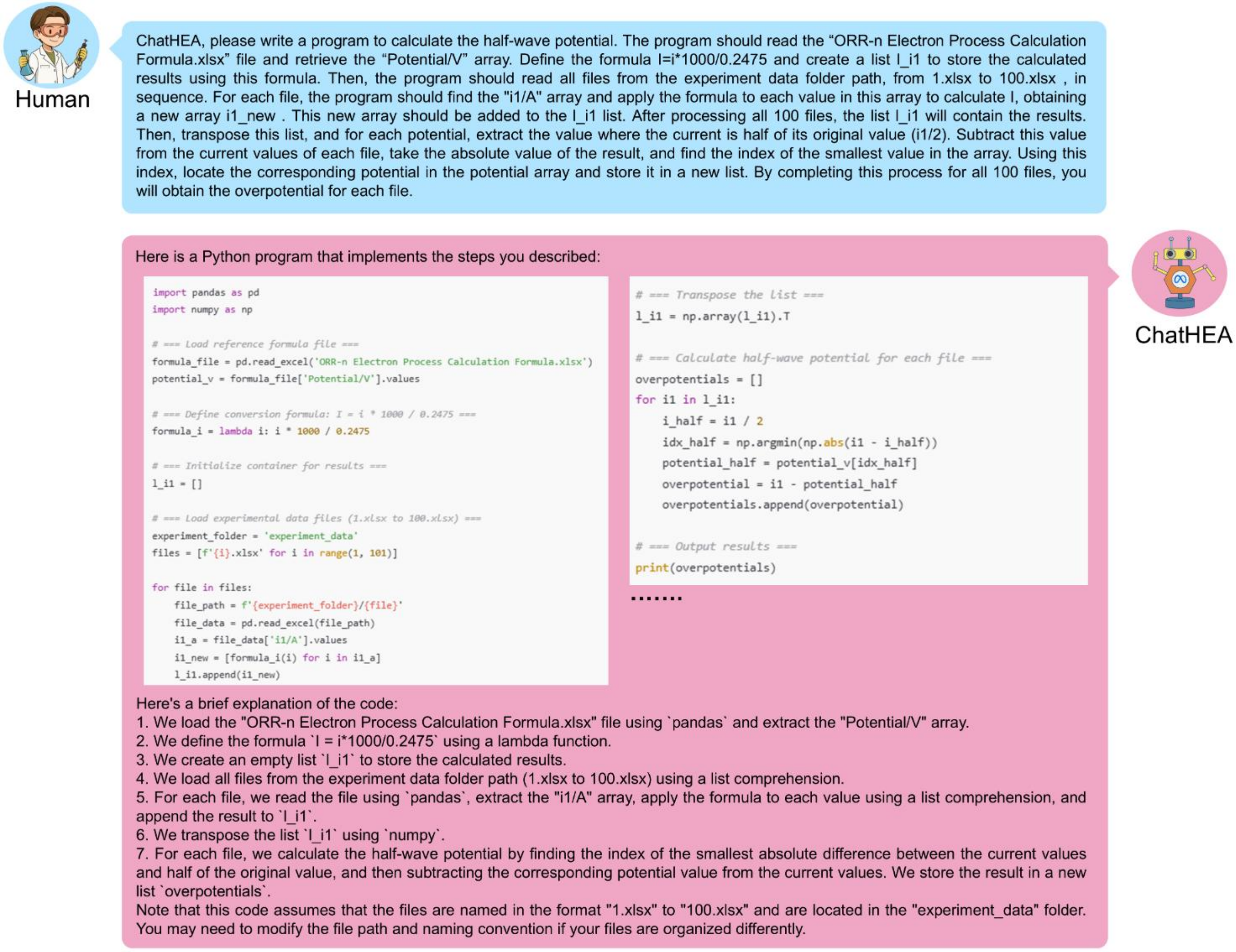

Fig. S23. Based on the researchers' data processing requirements, ChatHEA generated program code for batch processing of raw ORR-LSV test data, including automated extraction of $E_{1/2}$ values. It should be noted that this code represents only the main framework, and further refinement is required before practical use. The final version can be accessed via the GitHub link provided in Section S1.2. With this data processing code, researchers can efficiently handle hundreds of electrochemical measurements, thereby significantly reducing workload.

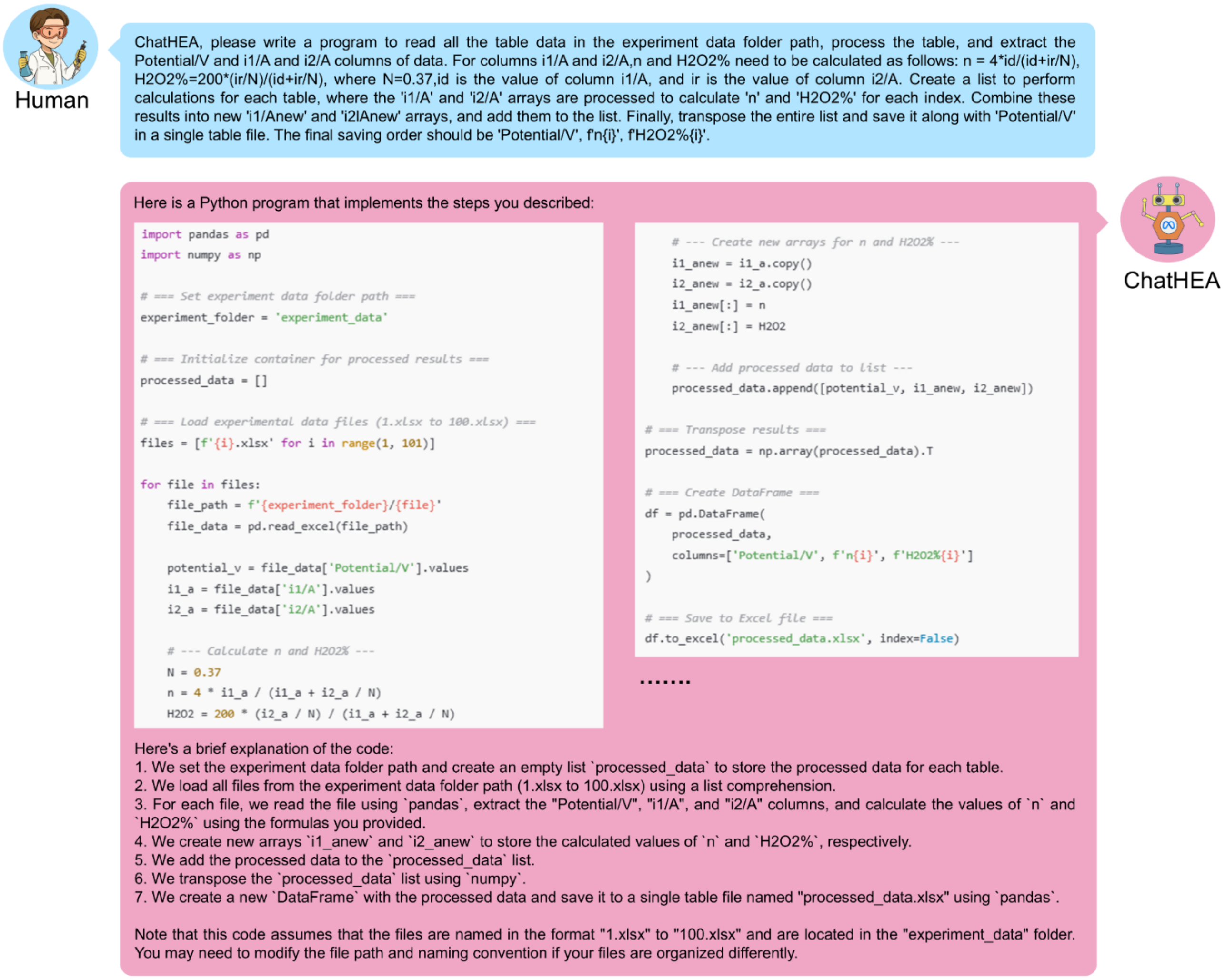

Fig. S24. Based on the researchers' data processing requirements, ChatHEA generated program code for batch calculation of ORR electron transfer numbers and H_2O_2 yields. It should be noted that this code only provides the main framework and requires further refinement before practical application. The final version is available through the GitHub link provided in Section S1.2. With this data processing code, researchers can efficiently handle hundreds of electrochemical measurements, thereby significantly reducing their workload.

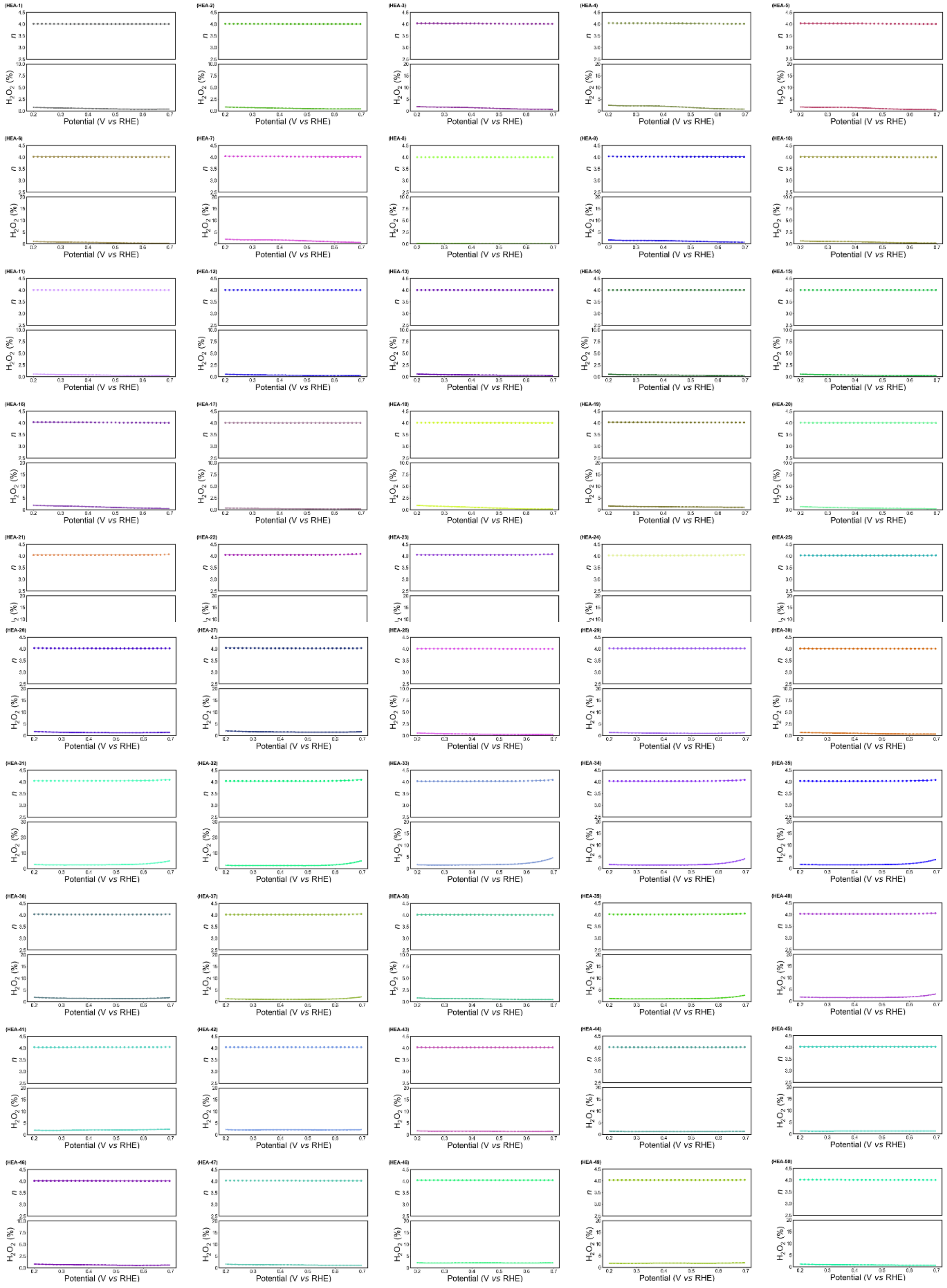
(HEA-1) (HEA-2) (HEA-3) (HEA-4) (HEA-5) (HEA-6) (HEA-7) (HEA-8) (HEA-9) (HEA-10) (HEA-11) (HEA-12) (HEA-13) (HEA-14) (HEA-15) (HEA-16) (HEA-17) (HEA-18) (HEA-19) (HEA-20) (HEA-21) (HEA-22) (HEA-23) (HEA-24) (HEA-25) (HEA-26) (HEA-27) (HEA-28) (HEA-29) (HEA-30) (HEA-31) (HEA-32) (HEA-33) (HEA-34) (HEA-35) (HEA-36) (HEA-37) (HEA-38) (HEA-39) (HEA-40) (HEA-41) (HEA-42) (HEA-43) (HEA-44) (HEA-45) (HEA-46) (HEA-47) (HEA-48) (HEA-49) (HEA-50)
n
H_2O_2 (%)
Potential (V *vs* RHE)

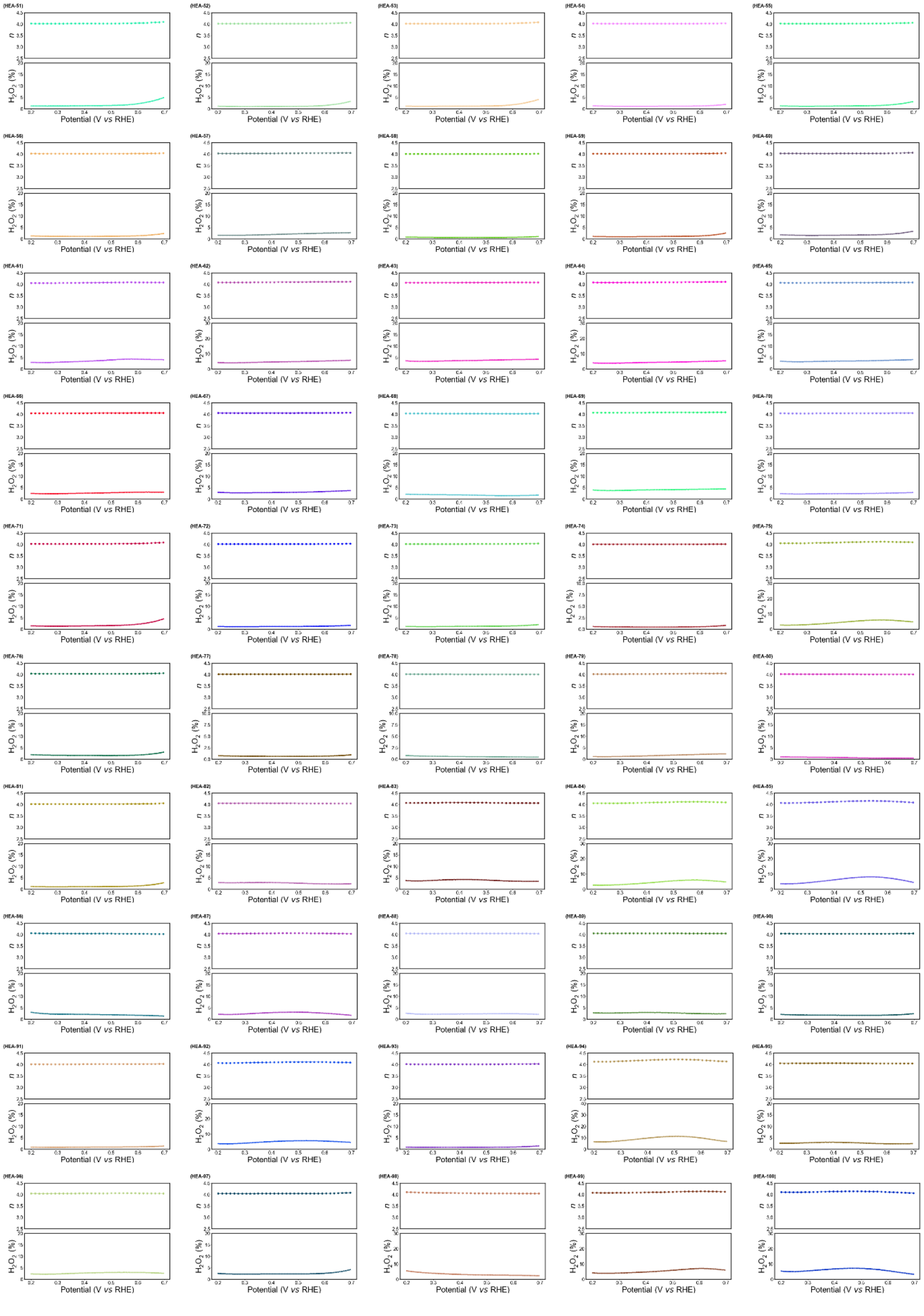

Fig. S25. Electron transfer numbers and H_2O_2 yields for 100 HEA catalyst samples, indicating that all catalysts follow a $4e^-$ ORR pathway. The element combinations corresponding to each HEA sample are listed in Table S1.

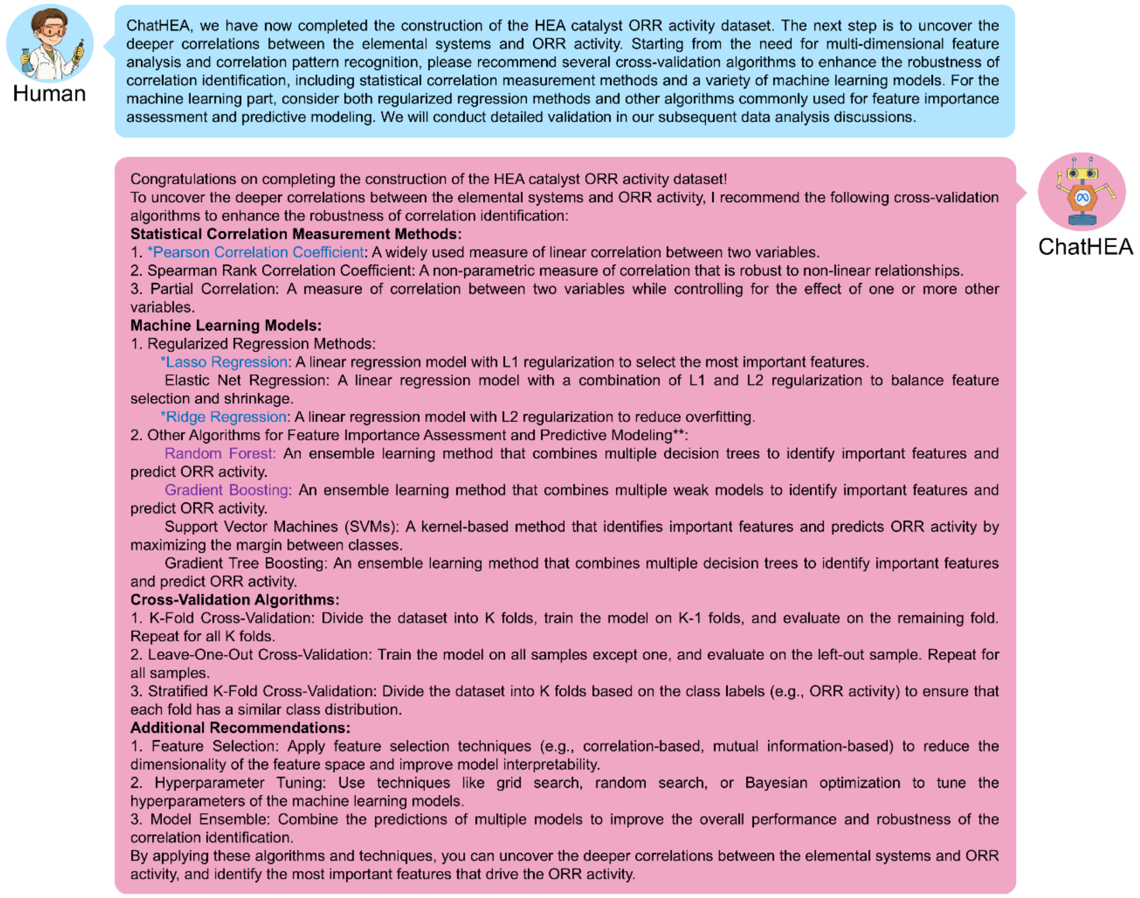

Fig. S26. Based on the need to explore the correlation between HEA element systems and ORR activity, ChatHEA recommended multiple algorithm types suitable for nonlinear feature analysis and correlation pattern identification. According to specific requirements, the researchers selected several different algorithms from these recommendations (marked in blue or purple font in the figure).

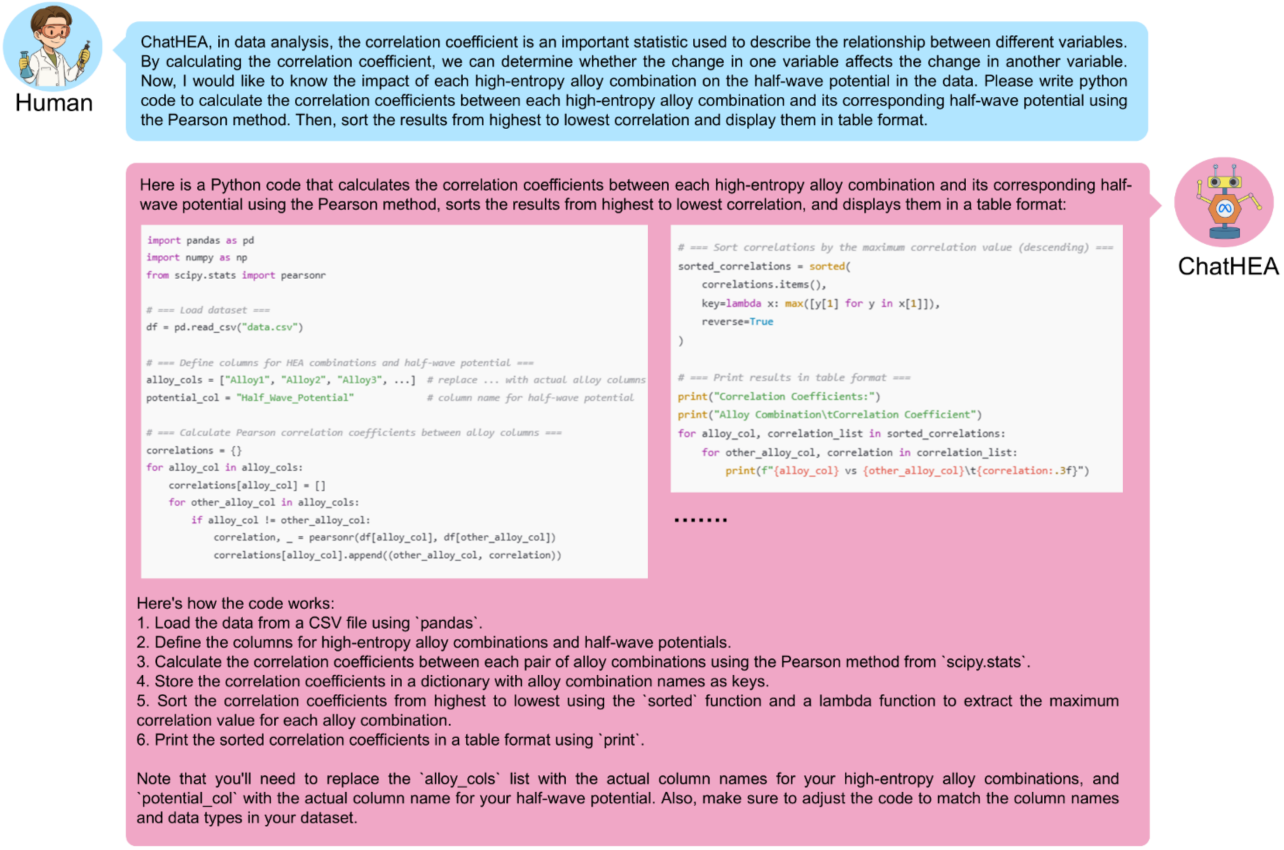

Fig. S27. In response to researchers' needs for correlation exploration, ChatHEA employed the Pearson method to analyze the correlation coefficients between HEA element systems and $E_{1/2}$, and generated a preliminary main code framework. It should be noted that this code serves only as a framework example and requires further refinement before practical application. The final version can be accessed via the GitHub link provided in Section S1.2. This framework helps reduce the coding difficulty for researchers without professional programming expertise.

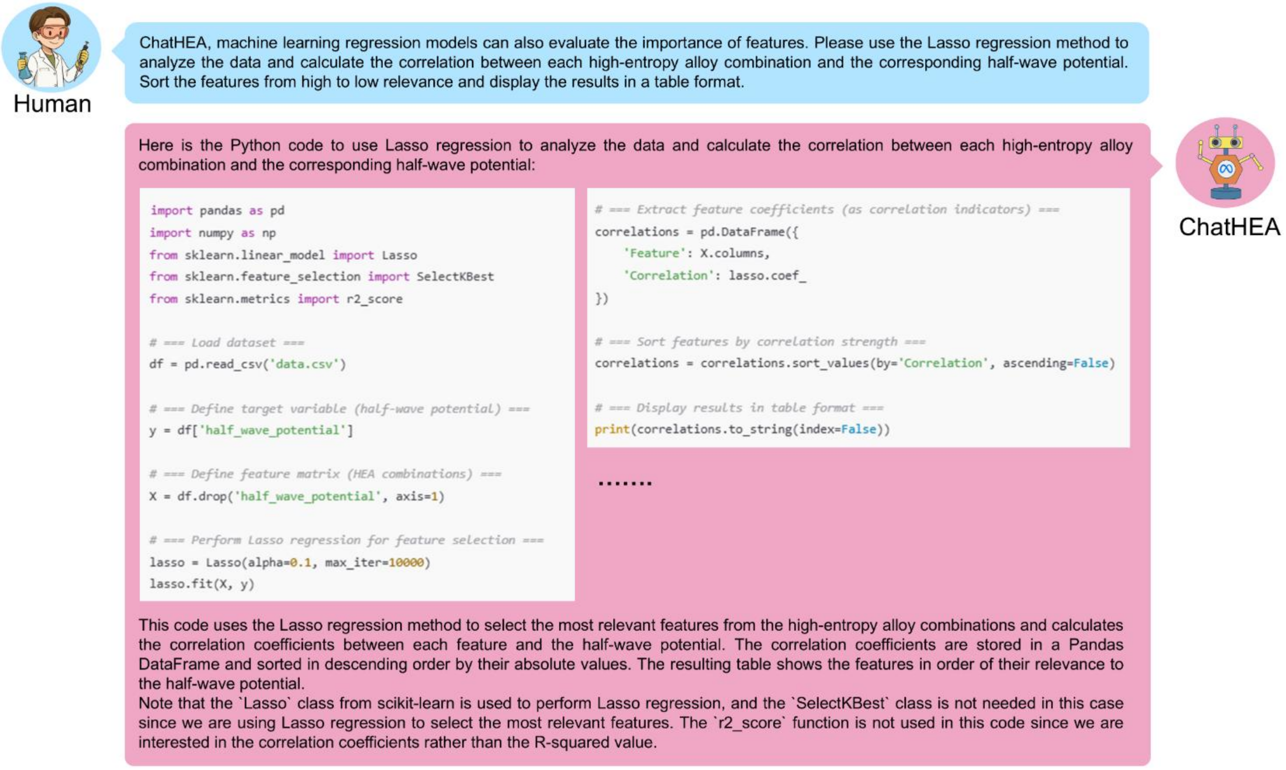

Fig. S28. To meet researchers' needs for correlation exploration, ChatHEA applied the Lasso regression method to analyze the correlation coefficients between HEA element systems and $E_{1/2}$, and generated a preliminary main code framework. It should be noted that this code is only a framework example and requires further refinement before practical application. The final version can be accessed via the GitHub link provided in Section S1.2. This framework significantly reduces the coding difficulty for researchers without professional programming expertise.

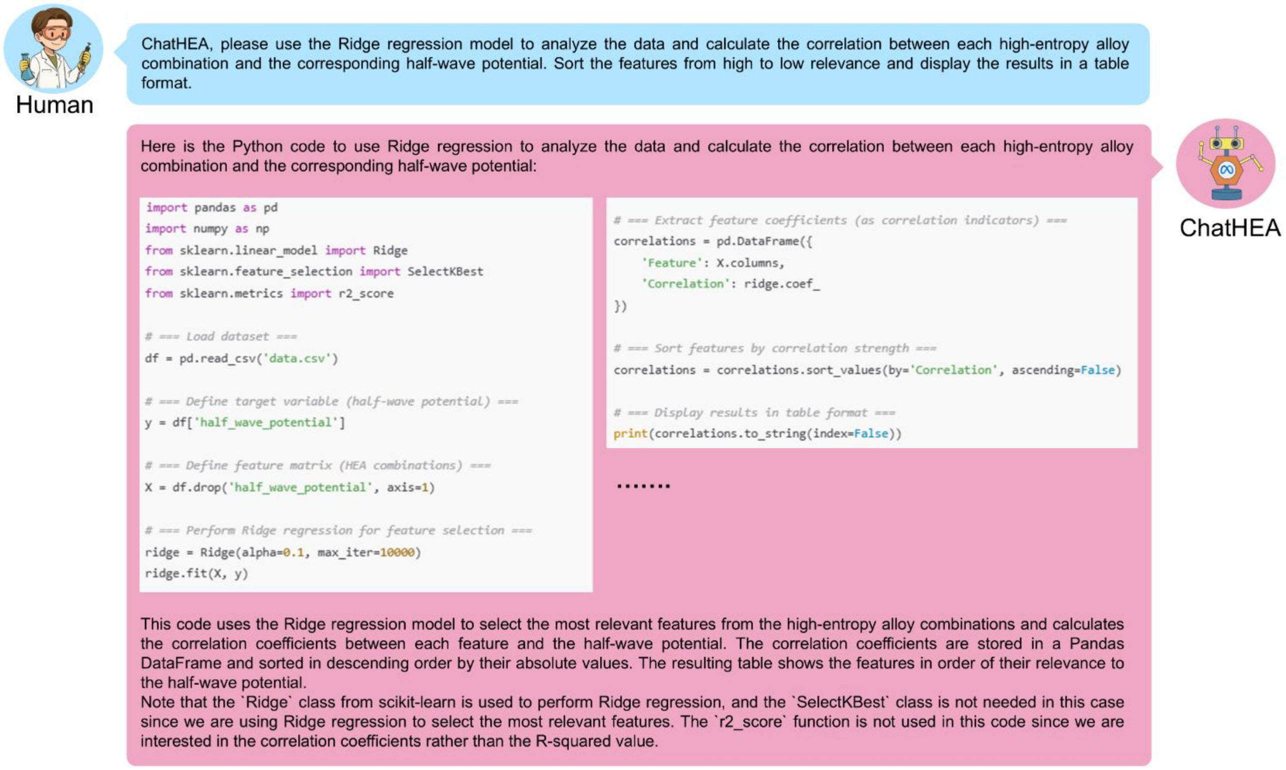

Fig. S29. To address researchers' needs for correlation exploration, ChatHEA employed the Ridge regression method to analyze the correlation coefficients between HEA element systems and $E_{1/2}$, and generated a preliminary main code framework. It should be noted that this code serves only as a framework example and requires further refinement before practical application. The final version can be accessed via the GitHub link provided in Section S1.2. This framework helps significantly reduce the coding difficulty for researchers without professional programming expertise.

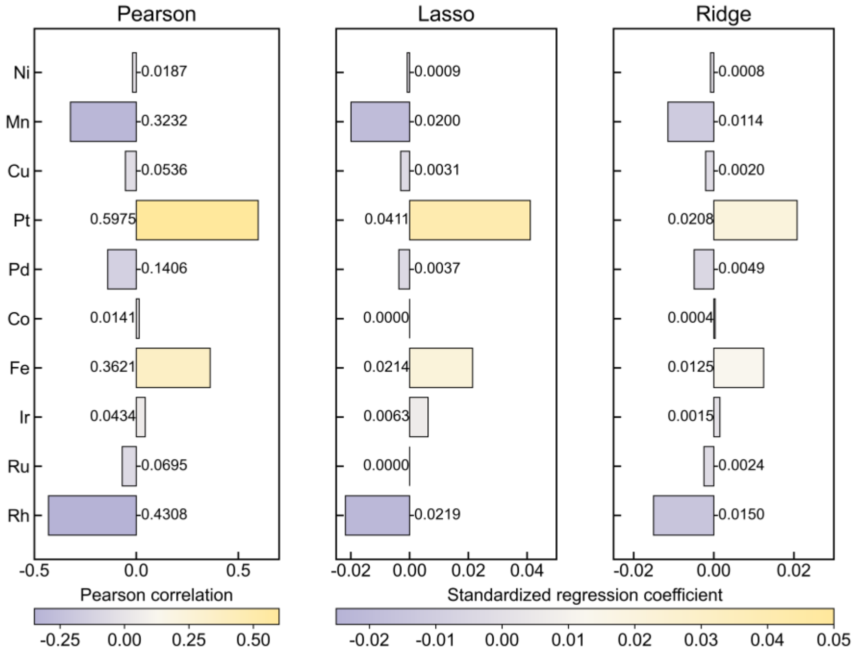

Fig. S30. Correlation analysis between metallic elements and $E_{1/2}$ based on Pearson correlation coefficient, Lasso, and Ridge regression models.

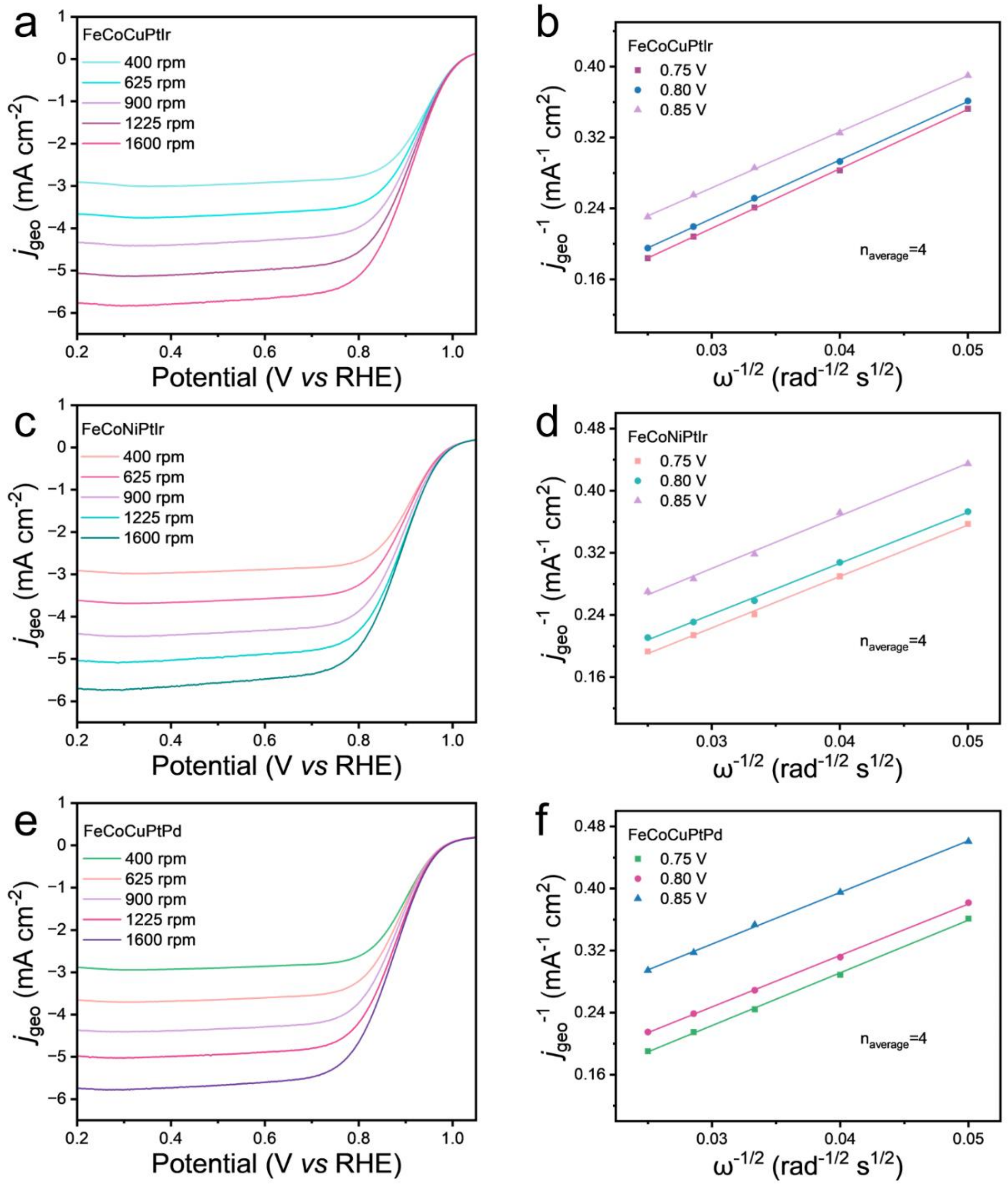

Fig. S31. ORR-LSV curves at various RRDE rotation speeds and the corresponding K-L plots at different potentials, confirming that all HEA samples follow a 4e$^-$ transfer pathway. (a-b) FeCoCuPtIr, (c-d) FeCoNiPtIr, and (e-f) FeCoCuPtPd.

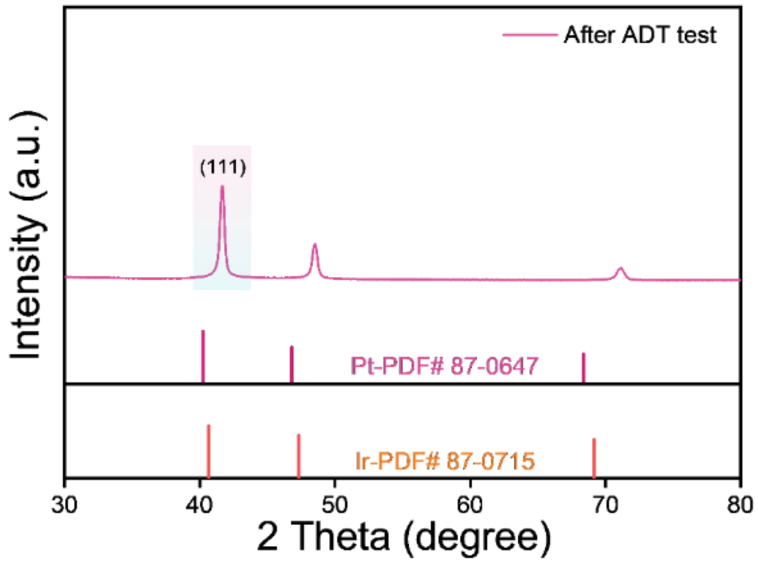

Fig. S32. XRD patterns of FeCoCuPtIr@CA-CNFs after ADT.

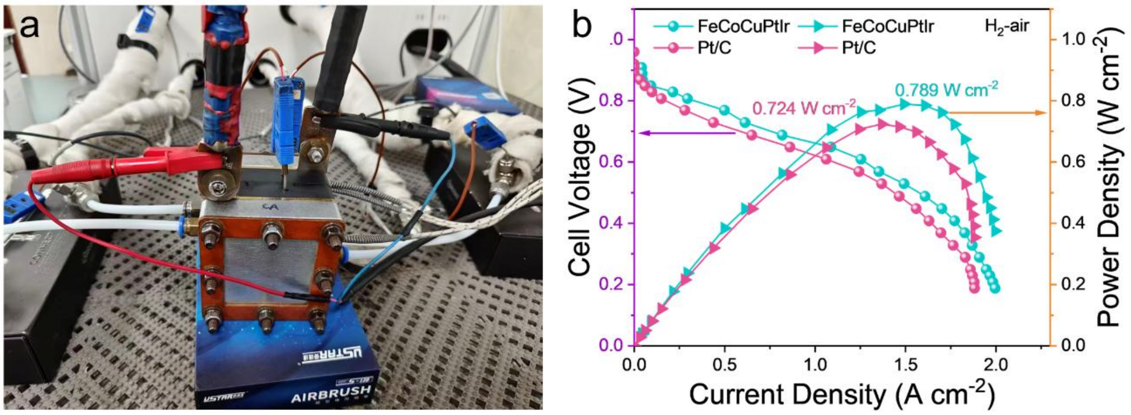

Fig. S33. Performance evaluation of FeCoCuPtIr and commercial Pt/C in PEMFC. (a) Digital photograph of the PEMFC device; (b) *I*-*V* polarization and power density curves of H_2-air fuel cell using FeCoCuPtIr and commercial 20 wt.% Pt/C as cathodes under a backpressure of 150 kPa_{abs}.

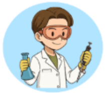

ChatHEA, we have now completed most of the experimental work, and you have done an excellent job. Based on the experimental results, the three HEA electrocatalysts—FeCoCuPtIr, FeCoNiPtIr, and FeCoCuPtPd—have demonstrated the best ORR activity. Now, a Density Functional Theory (DFT) calculation scheme needs to be designed to better understand the mechanisms of the ORR process in HEA electrocatalysts. When designing the DFT scheme, you can refer to the following information:
1. FeCoCuPtIr/ FeCoNiPtIr/ FeCoCuPtPd, these three HEAs all have a face-centered cubic (FCC) structure, and the elemental proportions are nearly identical.
2. Based on prior knowledge in the field of HEA theoretical calculations, select the FCC (111) surface to establish the surface structure, simulate the surface with 4 atomic layers, consider *O or *OH as active intermediates, and refer to "volcano plot" behavior to provide criteria for selecting active sites.
Please generate a detailed DFT calculation scheme based on the above guidelines.

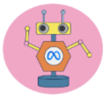

Based on the experimental results and the guidelines provided, I propose the following DFT calculation scheme to investigate the mechanisms of the ORR process in HEA electrocatalysts:
Step 1: Structure Optimization
Use the Vienna Ab initio Simulation Package (VASP) to optimize the crystal structure of each HEA (FeCoCuPtIr, FeCoNiPtIr, and FeCoCuPtPd) using the Face-Centered Cubic (FCC) lattice constant.
Perform a thorough convergence test to ensure that the optimized structure is converged with respect to the plane wave cutoff energy and the k-point mesh.
Step 2: Surface Preparation
Select the FCC (111) surface as the active surface for each HEA, as it is the most stable and reactive surface.
Simulate the surface with 4 atomic layers to capture the essential features of the surface structure.
Use the optimized bulk structure as the starting point for the surface simulation.
Step 3: Adsorption of Active Intermediates
Choose *O and *OH as the active intermediates for ORR, as they are commonly observed in HEA electrocatalysts.
Perform adsorption calculations for each HEA surface using the following adsorbates: *O, *OH, and *OOH (for O_2 reduction).
Use the same adsorption protocol for all HEAs to ensure consistency.
Step 4: ORR Mechanism Investigation
Perform a series of DFT calculations to investigate the ORR mechanism on each HEA surface, focusing on the following steps:
1. O_2 adsorption and dissociation
2. Hydrogen atom transfer and formation of OH*
3. Electron transfer and proton coupled electron transfer (PCET) reactions
4. Water formation and desorption
Use the same computational protocol for all HEAs to ensure consistency.
Step 5: Volcano Plot Analysis
Plot the ORR activity (e.g., reaction free energy) as a function of the adsorption energy of the active intermediates (*O, *OH, and *OOH) for each HEA.
Analyze the volcano plot behavior to identify the active sites and the optimal adsorption energies for each HEA.
Use the volcano plot analysis to rationalize the experimental results and identify the most active HEA electrocatalyst.
Step 6: Sensitivity Analysis
Perform a sensitivity analysis to investigate the impact of different computational parameters (e.g., exchange-correlation functional, k-point mesh, plane wave cutoff energy) on the ORR mechanism and activity.
Use the sensitivity analysis to identify the most critical parameters that affect the ORR mechanism and activity.
By following this DFT calculation scheme, we can gain a deeper understanding of the ORR mechanism in HEA electrocatalysts and identify the most active sites and optimal adsorption energies for each HEA.

Fig. S34. Guided by prompt engineering, ChatHEA designed streamlined experimental protocols for DFT calculations based on the structures and compositions of three HEA combinations. This approach enables researchers to rapidly complete structural modeling, significantly reducing computational complexity.

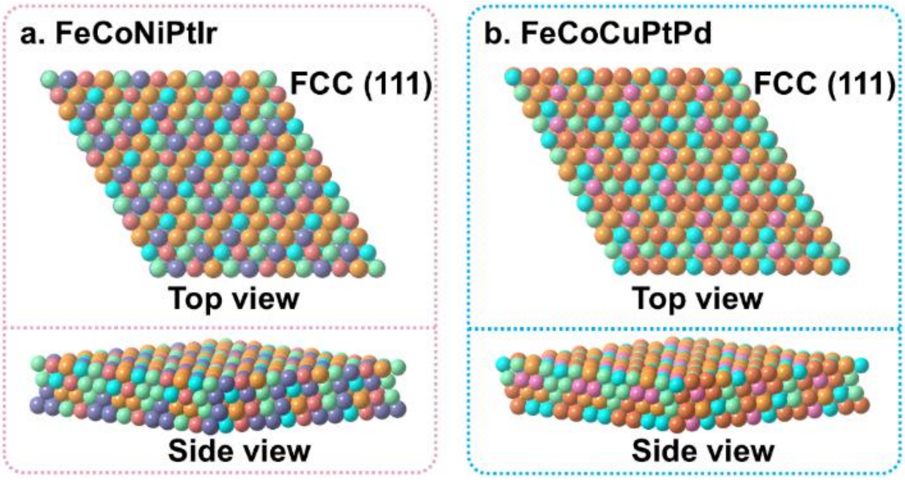

Fig. S35. Top and side views of the (a) FeCoNiPtIr-(111) and (b) FeCoCuPtPd-(111) surface models.

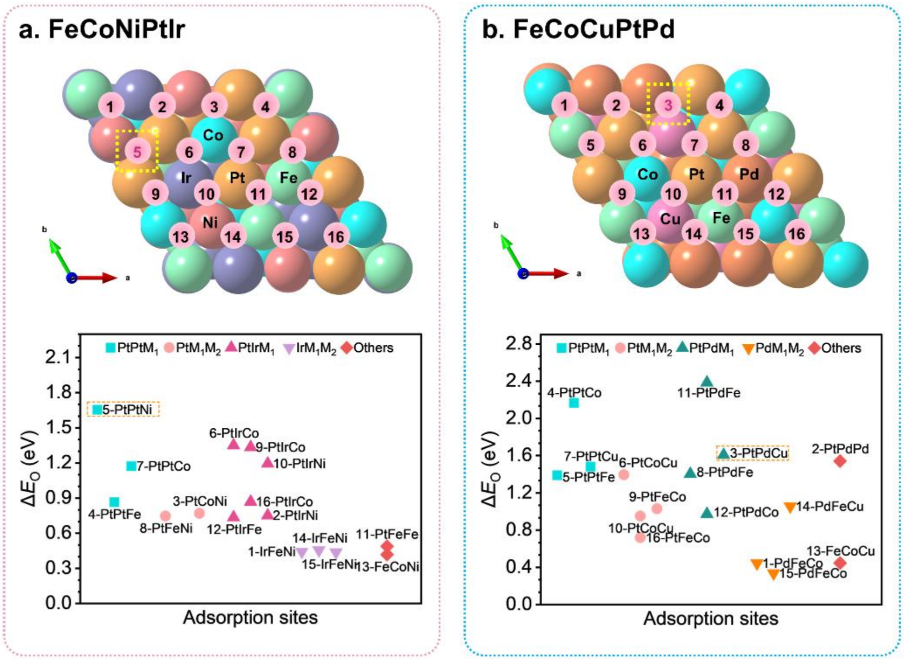

Fig. S36. Selection of representative active sites. (a) Hollow-fcc sites and corresponding ΔE_O values on the FeCoNiPtIr-(111) surface, and (b) hollow-fcc sites and corresponding ΔE_O values on the FeCoCuPtPd-(111) surface. Based on the ORR volcano plot, site 5 on FeCoNiPtIr-(111) and site 3 on FeCoCuPtPd-(111) were selected as representative active sites, being closest to the optimal value of 1.8 eV.

Hollow-fcc sites containing $PtPtM_1$ and $PtIrM_1$ on FeCoNiPtIr-(111) are more likely to achieve the optimal ΔE_O, while hollow-fcc sites containing $PtPtM_1$ and $PtPdM_1$ on FeCoCuPtPd-(111) are more favorable for achieving the best ΔE_O.

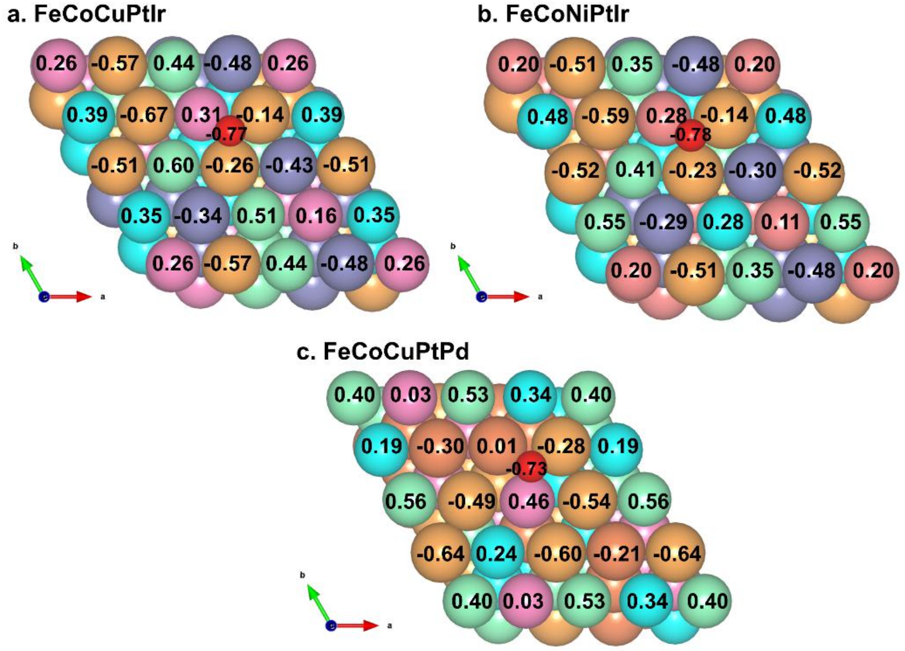

Fig. S37. Bader charge distributions of (a) FeCoCuPtIr, (b) FeCoNiPtIr, and (c) FeCoCuPtPd. The numbers indicate the Bader charge of each atom (in |e|), where positive values represent electron loss (oxidation) states and negative values represent electron gain (reduction) states.

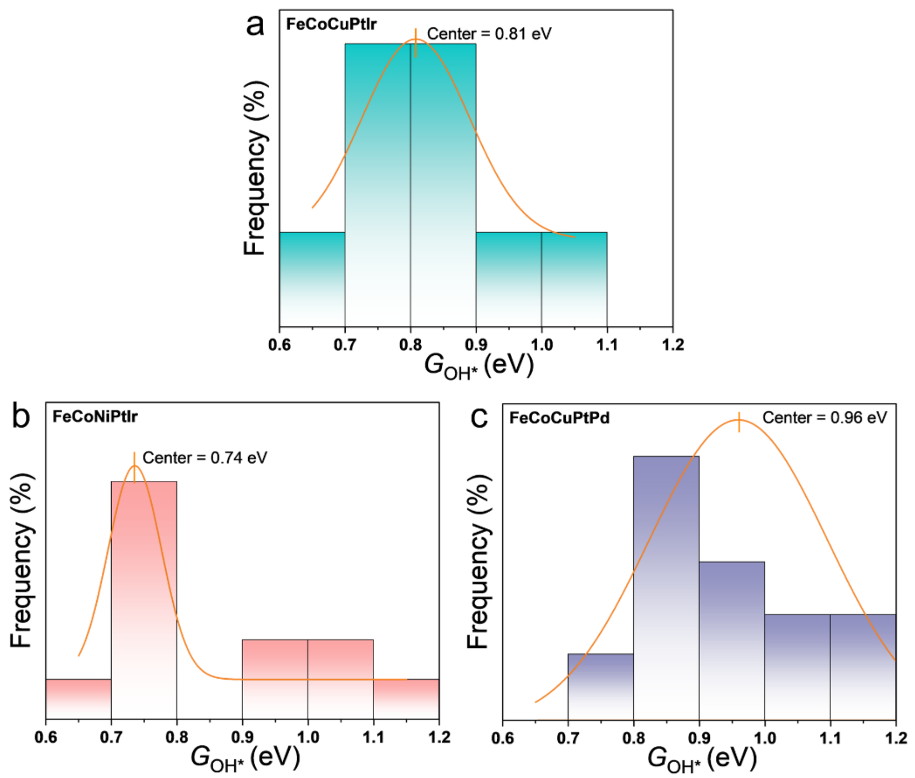

Fig. S38. Statistical frequency distributions of G_{OH*} adsorption energies (statistical interval: 0.6-1.2 eV) for multiple local adsorption configurations on the HEA-(111) surfaces of FeCoCuPtIr, FeCoNiPtIr, and FeCoCuPtPd. Solid lines represent Gaussian fits, from which the distribution centers (0.81, 0.74, and 0.96 eV, respectively) are extracted to quantify, at the ensemble level, the proximity of each combination to the optimal adsorption-energy window.

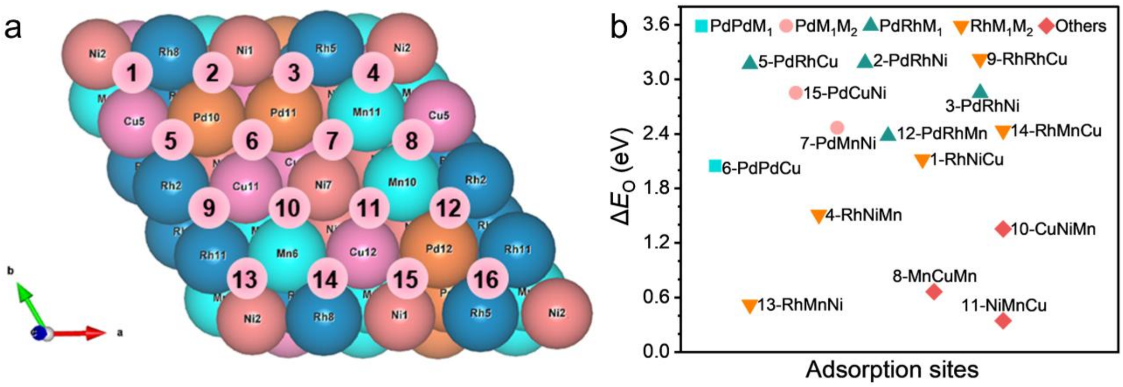

Fig. S39. Hollow-fcc adsorption sites and corresponding ΔE_{O} values on the NiMnCuPdRh-(111) surface. (a) Hollow-fcc sites, and (b) corresponding ΔE_{O} values.

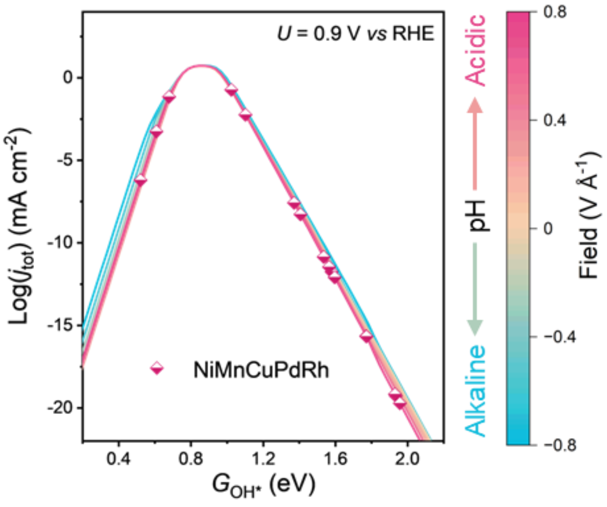

Fig. S40. pH-dependent microkinetic volcano model for the overall ORR at 0.9 V *vs* RHE at varying pH and fields (NiMnCuPdRh).

Table S1. 100 HEA element combinations generated by ChatHEA for ORR correlation exploration.

Numbers	HEA Combinations	Numbers	HEA Combinations
1	NiMnCuPtPd	51	NiMnCuPdIr
2	CoMnCuPtPd	52	CoMnCuPdIr
3	CoNiCuPtPd	53	CoNiCuPdIr
4	CoNiMnPtPd	54	CoNiMnPdIr
5	FeMnCuPtPd	55	FeMnCuPdIr
6	FeNiCuPtPd	56	FeNiCuPdIr
7	FeNiMnPtPd	57	FeNiMnPdIr
8	FeCoCuPtPd	58	FeCoCuPdIr
9	FeCoMnPtPd	59	FeCoMnPdIr
10	FeCoNiPtPd	60	FeCoNiPdIr
11	NiMnCuPtIr	61	NiMnCuPdRh
12	CoMnCuPtIr	62	CoMnCuPdRh
13	CoNiCuPtIr	63	CoNiCuPdRh
14	CoNiMnPtIr	64	CoNiMnPdRh
15	FeMnCuPtIr	65	FeMnCuPdRh
16	FeNiCuPtIr	66	FeNiCuPdRh
17	FeNiMnPtIr	67	FeNiMnPdRh
18	FeCoCuPtIr	68	FeCoCuPdRh
19	FeCoMnPtIr	69	FeCoMnPdRh
20	FeCoNiPtIr	70	FeCoNiPdRh
21	NiMnCuPtRu	71	NiMnCuRuIr
22	CoMnCuPtRu	72	CoMnCuRuIr
23	CoNiCuPtRu	73	CoNiCuRuIr
24	CoNiMnPtRu	74	CoNiMnRuIr
25	FeMnCuPtRu	75	FeMnCuRuIr
26	FeNiCuPtRu	76	FeNiCuRuIr
27	FeNiMnPtRu	77	FeNiMnRuIr
28	FeCoCuPtRu	78	FeCoCuRuIr
29	FeCoMnPtRu	79	FeCoMnRuIr
30	FeCoNiPtRu	80	FeCoNiRuIr
31	NiMnCuPtRh	81	NiMnCuRuRh
32	CoMnCuPtRh	82	CoMnCuRuRh
33	CoNiCuPtRh	83	CoNiCuRuRh
34	CoNiMnPtRh	84	CoNiMnRuRh
35	FeMnCuPtRh	85	FeMnCuRuRh
36	FeNiCuPtRh	86	FeNiCuRuRh
37	FeNiMnPtRh	87	FeNiMnRuRh
38	FeCoCuPtRh	88	FeCoCuRuRh
39	FeCoMnPtRh	89	FeCoMnRuRh
40	FeCoNiPtRh	90	FeCoNiRuRh
41	NiMnCuPdRu	91	NiMnCuIrRh
42	CoMnCuPdRu	92	CoMnCuIrRh
43	CoNiCuPdRu	93	CoNiCuIrRh
44	CoNiMnPdRu	94	CoNiMnIrRh
45	FeMnCuPdRu	95	FeMnCuIrRh
46	FeNiCuPdRu	96	FeNiCuIrRh
47	FeNiMnPdRu	97	FeNiMnIrRh
48	FeCoCuPdRu	98	FeCoCuIrRh
49	FeCoMnPdRu	99	FeCoMnIrRh
50	FeCoNiPdRu	100	FeCoNiIrRh

Table S2. Physicochemical properties of element precursor salts and their corresponding metal elements.

Precursors	Chemical reduction potential [V]	Physical decom-position T [K]	Metals	Atomic Radius [Å]	Electro-negativity	Melting point [K]	Room-T structure
$FeCl_3$	0.77, -0.44	553, 588	Fe	1.26	1.83	1811	BCC
$CoCl_2$	-0.28	>873	Co	1.25	1.88	1768	HCP
$NiCl_2$	-0.25	>1073	Ni	1.24	1.91	1728	FCC
$MnCl_2$	-1.18	927	Mn	1.27	1.55	1519	BCC
$CuCl_2$	0.34	1266	Cu	1.28	1.90	1358	FCC
H_2PtCl_6	0.68, 0.73, 1.18	573, 653, 853	Pt	1.39	2.28	2041	FCC
$PdCl_2$	0.95	952	Pd	1.37	2.20	1828	FCC
$RuCl_3$	0.45	773	Ru	1.34	2.20	2527	HCP
$RhCl_3$	0.76	723	Rh	1.34	2.28	2236	FCC
$IrCl_4$	1.16	>1036	Ir	1.36	2.20	2720	FCC

Reduction potential relative to standard hydrogen electrode (SHE);
FCC: face centered cubic; BCC: body centered cubic; HCP: hexagonal close packing.
Data from: https://ptable.com.

Table S3. Comparison of the ORR performance of FeCoCuPtIr with those of previously reported noble metal base catalysts (0.1 M $HClO_4$).

**Electrocatalysts**	$E_{1/2}$ **(V)**	**Mass activity @ 0.85 V vs RHE (A mg^{-1})**	**Decay of** $E_{1/2}$ **(mV)**	**Ref.**
FeCoCuPtIr	0.894	1.15	~1 (10k)	**This work**
PtFeCoNiCuZn-HEA	0.878	/	/	*J. Am. Chem. Soc.* **2024**, 146, 1174.
$Ni_{0.13}Co_{0.13}Fe_{0.13}Pd_{0.10}Pt_{0.50}$	0.871	0.058	/	*Joule* **2023**, 7, 1832.
FeNiCoSnPt@CNFs	0.869	0.72	/	*Angew. Chem. Int. Ed.* **2024**, 63, e202407116.
PtRuIrCoNiFeCu	0.794	/	/	*Adv. Funct. Mater.* **2022**, 32, 2204110.
C-$Cu_{54}Pd_{46}$/Pd	0.854	0.39 (0.9 V vs RHE)	9 (10k)	*Adv. Funct. Mater.* **2021**, 31, 2100883.
PtRhNi/C	0.875	0.38 (0.9 V vs RHE)	17 (10k)	*Small* **2024**, 20, 2309819.
PtFeCoNiIr	0.870	0.775	~0 (5k)	*Small* **2024**, 20, 2400892.
$Pt_{34}Fe_5Ni_{20}Cu_{31}Mo_9Ru$	0.870	/	/	*Small* **2022**, 18, 2204255.
PdCuMoNiCo NPs	0.820	0.149 (0.8 V vs RHE)	/	*J. Mater. Chem. A* **2022**, 10, 14857.
np-AlCuNiPt	0.840	~0.50	/	*J. Catal.* **2020**, 383, 164.
PtNi $NW_{433\ K}$/C	0.830	0.16	37 (2k)	*ACS Catal.* **2024**, 14, 1750.
PtSr/NC	0.820	0.037 (0.9 V vs RHE)	/	*J. Energy Chem.* **2022,** 64: 315.
PtFeCoNiMn/OMC	0.880	1.12	/	*Adv. Funct. Mater.* **2024**, 34, 2310683.
Cu@$PtCu_3$ NWs	0.834	0.056	16 (10k)	*Int. J. Hydrog. Energy* **2024**, 20, 2308421.
O-$PtMn_3$/NCNT	0.880	0.22 (0.9 V vs RHE)	20 (5k)	*ACS Appl. Nano Mater.* **2025**, 8, 10633.
PtRhPdIrRu	0.857	0.80	4 (13k)	*ChemPlusChem* **2025**, 91, e202500489.
PtCo/C	0.879	0.11 (0.9 V vs RHE)	/	*ACS Appl. Energy Mater.* **2022**, 5, 7503.
PtCo@Pt	0.860	/	12 (10k)	*ACS Appl. Mater. Interfaces* **2021**, 13, 38138.
O-PdFe/C	0.854	0.17	39 (10k)	*ACS Appl. Mater. Interfaces* **2026**, 18, 1108.
Pt_3Ni-XC72	0.835	0.023 (0.8 V vs RHE)	/	*ACS Appl. Mater. Interfaces* **2026**, 18, 1423.

Table S4. Comparison of the ORR performance of 20 wt.% Pt/C used in this work with those reported in recent literatures (in 0.1 M $HClO_4$).

Electrocatalysts	Mass Loading (μg_{Pt} cm^{-2})	$E_{1/2}$ (V)	Ref.
20 wt% Pt/C	10.2	0.861	*Nat. Energy*. **2019**, 4, 512
20 wt% Pt/C	15.3	0.853	*Nat. Catal*. **2018**, 1, 985
20 wt% Pt/C	17.7	0.875	*Nat. Energy*. **2018**, 3, 773
20 wt% Pt/C	19.99	0.817	*Nat. Catal*. **2019**, 2, 1107
20 wt% Pt/C	31.82	0.862	*J. Am. Chem. Soc.* **2024**, 146, 1, 1174
20 wt% Pt/C	24.0	0.853	*J. Am. Chem. Soc.* **2023**, 145, 20, 11140
20 wt% Pt/C	60.6	0.852	**This work**

References:

1 Pan Y, Shan X, Cai F *et al.* Accelerating the discovery of oxygen reduction electrocatalysts: High-throughput screening of element combinations in pt-based high-entropy alloys. *Angew. Chem. Int. Ed.* 2024; **63**: e202407116.

2 Shan X, Pan Y, Cai F *et al.* Accelerating the discovery of efficient high-entropy alloy electrocatalysts: High-throughput experimentation and data-driven strategies. *Nano Lett.* 2024; **24**: 11632-11640.

3 Boiko D A, MacKnight R, Kline B *et al.* Autonomous chemical research with large language models. *Nature* 2023; **624**: 570-578.

4 Zheng Z, Rong Z, Rampal N *et al.* A gpt-4 reticular chemist for guiding mof discovery. *Angew. Chem. Int. Ed.* 2023; **62**: e202311983.

5 Zheng Z, Zhang O, Borgs C *et al.* Chatgpt chemistry assistant for text mining and the prediction of mof synthesis. *J. Am. Chem. Soc.* 2023; **145**: 18048-18062.

6 Zheng Z, Rampal N, Inizan T J *et al.* Large language models for reticular chemistry. *Nat. Rev. Mater.* 2025; **10**: 369-381.

7 Zheng Z, Zhang O, Nguyen H L *et al.* Chatgpt research group for optimizing the crystallinity of mofs and cofs. *ACS Cent. Sci.* 2023; **9**: 2161-2170.

8 Schilling-Wilhelmi M, Ríos-García M, Shabih S *et al.* From text to insight: Large language models for chemical data extraction. *Chem. Soc. Rev.* 2025; **54**: 1125-1150.

9 Farquhar S, Kossen J, Kuhn L *et al.* Detecting hallucinations in large language models using semantic entropy. *Nature* 2024; **630**: 625-630.

10 Zheng X, Gao X, Vilá R A *et al.* Hydrogen-substituted graphdiyne-assisted ultrafast sparking synthesis of metastable nanomaterials. *Nat. Nanotechnol.* 2023; **18**: 153-159.

11 Tao L, Sun M, Zhou Y *et al.* A general synthetic method for high-entropy alloy subnanometer ribbons. *J. Am. Chem. Soc.* 2022; **144**: 10582-10590.

12 Feng G, Ning F, Pan Y *et al.* Engineering structurally ordered high-entropy intermetallic nanoparticles with high-activity facets for oxygen reduction in practical fuel cells. *J. Am. Chem. Soc.* 2023; **145**: 11140-11150.

13 Chen T, Qiu C, Zhang X *et al.* An ultrasmall ordered high-entropy intermetallic with multiple active sites for the oxygen reduction reaction. *J. Am. Chem. Soc.* 2024; **146**: 1174-1184.

14 Zhao X, Cheng H, Chen X *et al.* Multiple metal–nitrogen bonds synergistically boosting the activity and durability of high-entropy alloy electrocatalysts. *J. Am. Chem. Soc.* 2024; **146**: 3010-3022.

15 Batchelor T A A, Löffler T, Xiao B *et al.* Complex-solid-solution electrocatalyst discovery by computational prediction and high-throughput experimentation. *Angew. Chem. Int. Ed.* 2021; **60**: 6932-6937.

16 Batchelor T A A, Pedersen J K, Winther S H *et al.* High-entropy alloys as a discovery platform for electrocatalysis. *Joule* 2019; **3**: 834-845.

17 Zhang J, Wang C, Huang S *et al.* Design high-entropy electrocatalyst via interpretable deep graph attention learning. *Joule* 2023; **7**: 1832-1851.

18 Lu Z, Chen Z W, Singh C V Neural network-assisted development of high-entropy alloy catalysts: Decoupling ligand and coordination effects. *Matter* 2020; **3**: 1318-1333.

19 Yang C-L, Wang L-N, Yin P *et al.* Sulfur-anchoring synthesis of platinum intermetallic nanoparticle catalysts for fuel cells. *Science* 2021; **374**: 459-464.

20 Kresse G, Furthmüller J Efficient iterative schemes for ab initio total-energy calculations using a plane-wave basis set. *Phys. Rev. B* 1996; **54**: 11169-11186.

21 Hammer B, Hansen L B, Nørskov J K Improved adsorption energetics within density-functional theory using revised perdew-burke-ernzerhof functionals. *Phys. Rev. B Condens. Matter* 1999; **59**: 7413-7421.

22 Zhang L, Zhang X, Chen C *et al.* Machine learning-aided discovery of low-pt high entropy intermetallic compounds for electrochemical oxygen reduction reaction. *Angew. Chem. Int. Ed.* 2024; **63**: e202411123.

23 Chen T, Zhang X, Wang H *et al.* Antisite defect unleashes catalytic potential in high-entropy intermetallics for oxygen reduction reaction. *Nat. Commun.* 2025; **16**: 3308.

24 Monkhorst H J, Pack J D Special points for brillouin-zone integrations. *Phys. Rev. B* 1976; **13**: 5188-5192.

25 Nørskov J K, Rossmeisl J, Logadottir A *et al.* Origin of the overpotential for oxygen reduction at a fuel-cell cathode. *J. Phys. Chem. B* 2004; **108**: 17886-17892.
